# Oligomers of cyclopentadithiophene-vinylene in aromatic and quinoidal versions and redox species with intermediate forms†
†Electronic supplementary information (ESI) available: Full experimental and spectroscopic data. CCDC 1570290. For ESI and crystallographic data in CIF or other electronic format see DOI: 10.1039/c7sc02756g


**DOI:** 10.1039/c7sc02756g

**Published:** 2017-09-27

**Authors:** Paula Mayorga Burrezo, Rocío Domínguez, José L. Zafra, Ted M. Pappenfus, Pilar de la Cruz, Lorena Welte, Daron E. Janzen, Juan T. López Navarrete, Fernando Langa, Juan Casado

**Affiliations:** a Department of Physical Chemistry , University of Málaga , Campus de Teatinos s/n , Málaga , 29071 , Spain . Email: casado@uma.es; b Department of Molecular Nanoscience and Organic Materials , Institut de Ciència de Materials de Barcelona (ICMAB)/CIBER-BBN , Campus Universitari de Bellaterra , E-08193 , Cerdanyola , Barcelona , Spain; c University of Castilla-La Mancha , Institute of Nanoscience , Nanotechnology and Molecular Materials (INAMOL) , Campus de la Fábrica de Armas , 45071-Toledo , Spain; d Division of Science and Mathematics , University of Minnesota , Morris , Minnesota 56267 , USA; e Department of Chemistry and Biochemistry , St. Catherine University , St. Paul , Minnesota 55105 , USA

## Abstract

A new series of π-conjugated oligomers based on the 4,4 dihexyl-4*H*-cyclopenta[2,1-*b*:3,4-*b*′]dithiophene vinylene repeating unit has been prepared and characterized by X-ray, electrochemical, spectroscopic (UV-Vis absorption, emission and Raman) and density functional theory methods. The oligomers in their neutral, oxidized and reduced forms have been investigated. The neutral compounds show a longer mean conjugation length than oligothiophenes and oligothiophene-vinylenes and display very rich redox chemistry with the stabilization of polycationic states of which the radical cations and dications are strong NIR absorbers, the latter displaying singlet diradicaloid character. An interesting complementarity between the sequence of aromatic-quinoidal structural segments in the radical cations and dications has been described and interpreted. Two derivatives with the 4,4 dihexyl-4*H*-cyclopenta[2,1-*b*:3,4-*b*′]dithiophene vinylene unit, disubstituted either with electron donor, bis(triaryl amino) groups, or acceptors bis(dicyano-methylene) caps enforcing a quinoidal structure in the dithiophene-vinylene bridge, have been also synthesized and characterized. The radical cation of the triarylamine compound and the radical anion of the tetracyano compound similarly display hole and electron charge localization, or confinement, in the nitrogen and dicyano surrounding parts, or class II mixed valence systems, while their dication and dianion species, conversely, are open-shell diradical (*i.e.*, polaron pair) and closed-shell (*i.e.*, bipolaron), respectively. The preparation of these new π-conjugated oligomers gives way to the realization of compounds with new electronic properties and unique structures potentially exploitable in organic electronics.

## Abstract 2

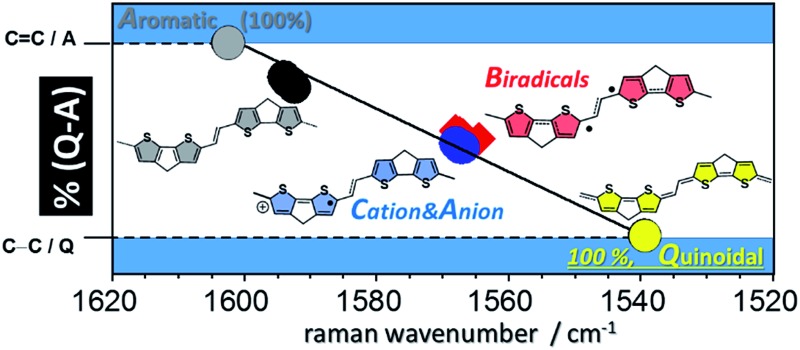
Quinoidal-*vs.*-aromatic synergy: Cyclopentadithiophene-vinylene oligomers with different sizes, in different oxidation states and as pro-aromatic quinoidals are studied considering balanced contributions of aromatic and quinoidal forms.

## Introduction

Oligothiophenes (OTs) represent the prototypical molecules where, due to the weak aromaticity of thiophene, π-electron conjugation is sizeable and very appropriate for the implementation as semiconductor materials in optoelectronic applications.^1^ A further modulation/improvement of π-conjugation of OTs has been achieved by intercalation with different functionalities as, for example, with vinylenes or oligothiophene-vinylenes (*i.e.*, TVs).^2^

In the field of organic electronics, where new materials with new/enhanced opto-electronic properties for device applications are desired, we here explore another combination of the thiophene/vinylene couple in which a 4,4-dihexyl-4H-cyclopenta[2,1-*b*:3,4-*b*′]dithiophene (**CPDT**) unit replaces the thiophene in OTVs to form **CPDTV** oligomers.^3^–^6^

The **CPDT** group has been extensively used in the construction of conducting polymers due to the excellent π-electron donor ability, ease of preparation and functionalization, large solubility by incorporation of alkyl chains at the 4-position without interference in the π-system, and complete planarization of the 12 e^–^ fused π-dithiophene.^3^–^6^ The **CPDT** unit is a versatile building block because of its electronic behavior duality: on one hand, the two *cis*-connected thiophenes allows **CPDT** to behave as an electron donor and hence it has been used in π-electron donor–acceptor conjugated copolymers.^5^**CPDT**-based donor–acceptor polymers have been successfully implemented in numerous device applications, such as polymeric field-effect transistors and photovoltaics.^6^ On the other hand, the central cyclopentadienyl moiety can act as an electron acceptor group, where different types of substitutions on the 4-position of the **CPDT** unit has been explored in order to enhance the electron-withdrawing character.^6^

**Scheme 1:**
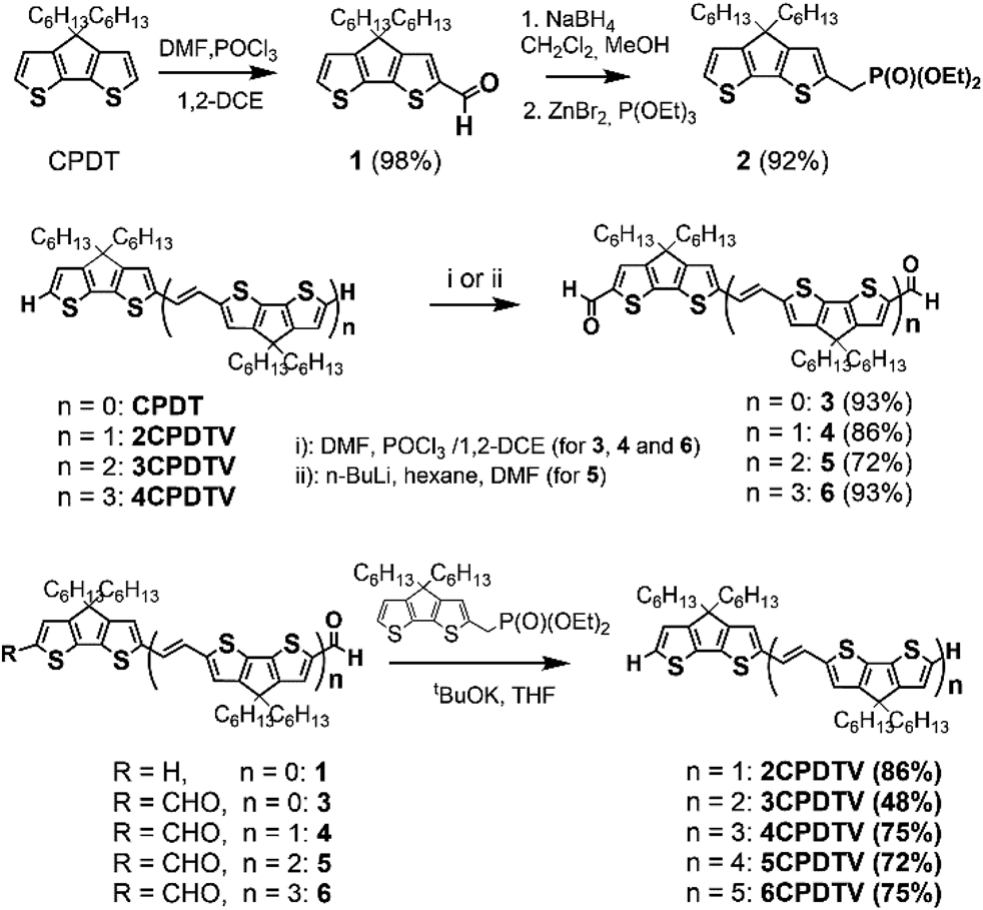
Synthesis for ***n*CPDTV** oligomers with *n* = 1–6.

The field of organic electronics partially lacks the existence of reliable structure-property-function relationships on which *a priori* chemical designs of improved molecules and substrates could be based. Therefore, it is important to investigate the optoelectronic and structural properties of new oligomers as a function of oligomer length, (*i.e.*, the oligomer approach),^7^ which can serve as the first step in analyzing structure–property connections for the development of new organic semiconductors. This paper utilizes this approach and outlines the investigation of the electronic and structural properties of π-conjugated compounds based on the **CPDT** unit alternating with double bonds to create a new oligomeric series from a dimer to a hexamer (*i.e.*, ***n*CPDTV** with *n* = 1–6 in Scheme 1). The study is based on the X-ray solid state, electrochemical, optical (absorption and emission) and vibrational Raman characterization of their properties as a function of the oligomer size and in their neutral and charged states. The study is completed with the analysis of two versions of **CPDTV**, either substituted with electron-donor groups where the **CPDTV** core keeps a thieno-aromatic structure or with electron-acceptor groups where, conversely, the **CPDTV** core displays a thieno-quinoidal shape.

## Results and discussion

### Synthesis of ***n*CPDTV**s

The synthesis of ***n*CPDTV** oligomers was accomplished by iterative Vilsmeier bisformylation and Horner–Wadsworth–Emmons double olefination according to Scheme 1. Phosphonate derivative **2** was prepared in 92% yield by reduction of monoaldehyde **1** with sodium borohydride followed by zinc bromide-mediated Michaelis–Arbuzov reaction. Bisaldehydes **3–6** were obtained by double Vilsmeier formylation in good yields (86–93%) except for compound **5**. In this case the synthesis was performed in 72% yield by using *n*-BuLi and DMF (see ESI†). Finally, ***n*CPDTVs** were synthesized in good yields by means of Horner–Wadsworth–Emmons reactions in the presence of potassium tert-butoxide as the base. Purification was performed using column chromatography and then recrystallization from hexane/methanol to provide the final oligomers with high purity. The synthetic details and characterization data are collected in the ESI file (see Sections 1–3, Fig. S1–S46†). All oligomers exhibited excellent thermal stability with decomposition temperatures around 400 °C (see Fig. S47†)

### Unsubstituted ***n*CPDTVs**: optical and structural properties in the neutral state

**Fig. 1:**
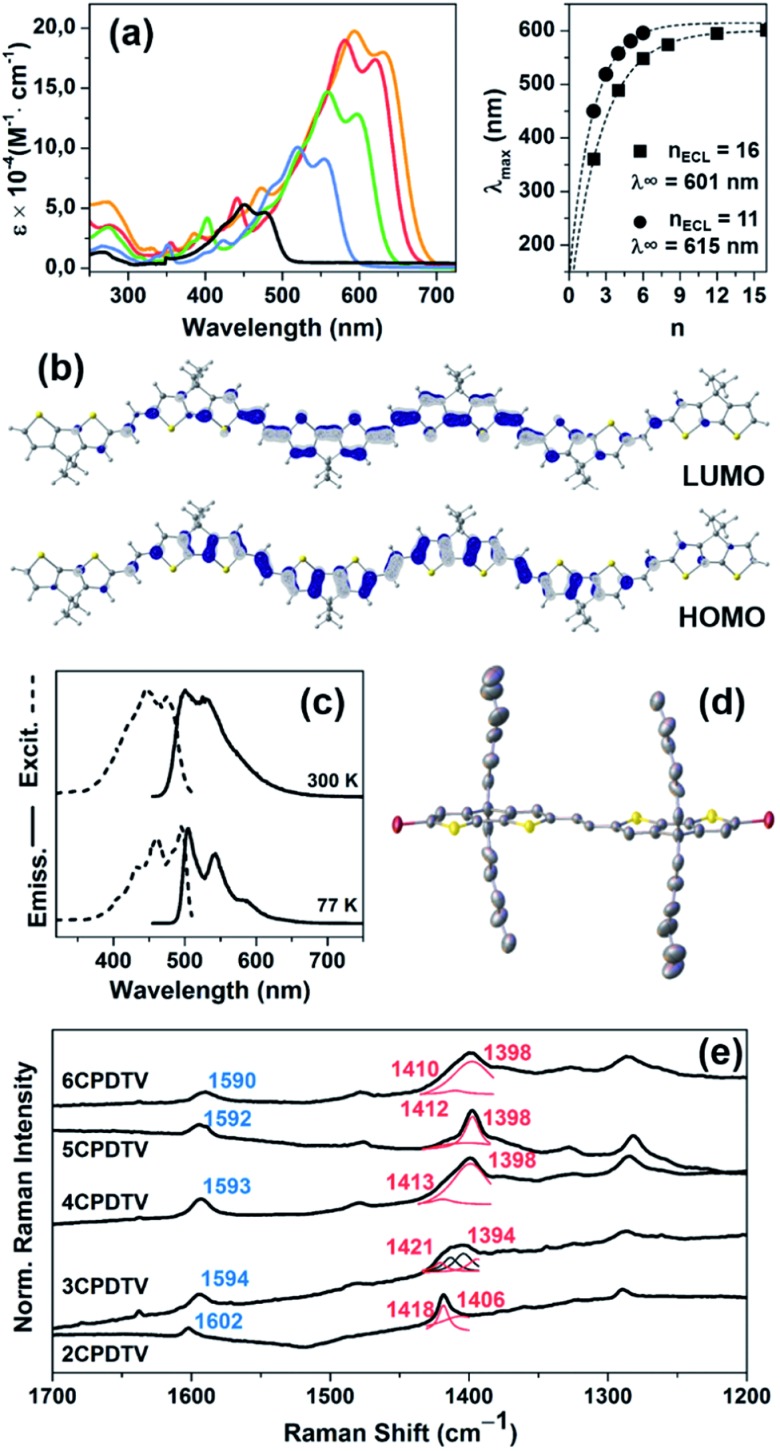
(a) Left: UV-Vis absorption spectra of ***n*CPDTV** in CH_2_Cl_2_. Right: Meier's plot fitting for ***n*CPDTV** (circles) and ***n*TV** series (squares) and their effective conjugation length (ECL) and the absorption maximum of the infinite chain, (*λ*_∞_). (b) DFT/B3LYP/6-31G** Frontier molecular orbital for **6CPDTV**. (c) Excitation (dashed lines) and emission (solid lines) spectra of **2CPDTV** at 77 K and room temperature. (d) X-ray structure of **2CPDTV-Br** (50% thermal ellipsoids). (e) Solid-state Raman (*λ* = 785 nm) spectra of ***n*CPDTV** recorded at room temperature. *ν*(C=C)_vinyl_ in blue, *ν*(C=C)_CPDT_ in red. The frequencies have been assigned by deconvolution.

Fig. 1(a) displays the electronic absorption spectra of the ***n*CPDTV** compounds in solution. These spectra show broad bands with clear vibronic structure, which progressively red-shift nearly 150 nm from **2CPDTV** to **6CPDTV**. Simultaneously, absorption coefficients increase in the series with increasing oligomer size. This set of wavelength peak maxima can be adjusted to a Meier's plot in order to estimate the effective conjugation length^8^ (*n*_ECL_, Fig. 1(a)) from which values of *n*_ECL_ = 11 units and a *λ*_∞_ = 615 nm are obtained – indicating that the *λ*_max_ = 594 nm in the hexamer is already close to the values in the ideal infinite polymer. The *n*_ECL_ = 11 can be compared with that in OTV oligomers where *n*_ECL_ = 16,2*a* which reflects the larger π-conjugation of the **CPDTV** unit (14-π e^–^ in **CPDTV** unit compared to and 8-π e^–^ in TV, or 154 and 128 electrons, respectively). This longest wavelength absorption band is due to a one-electron excitation from the HOMO to the LUMO (Fig. 1(b)). Whereas the electron density is fully delocalized over the entire backbone in smaller oligomers, this is concentrated in the middle for the longer ones without extension into the terminal rings. This aspect would justify the saturation behavior of *λ*_max_ for **6CPDTV**.

An emission study of **2CPDTV** has been carried out by measuring the fluorescence spectra in solution at room temperature and at liquid nitrogen temperature (Fig. 1(c)). The emission spectrum at room temperature is mirror-like with the absorption indicating that both originate from the same S_0_ → S_1_ excitation (S_1_ → S_0_ emission). The small Stokes shift is an indication of the small conformational reorganization upon excitation, as it is expected for a molecule with only two degrees of dihedral rotation. At 77 K, the vibronic structure is better resolved and the Stokes shift has almost vanished due to minimization of motion around the vinylene bridge. In this context, the X-ray structure of **2CPDTV-Br** (*i.e.*, **2CPDTV** with two terminal bromine atoms) has been resolved and is shown in Fig. 1(d) (Fig. S48 and S49 and Table S1†). The structure shows a highly planar π-backbone with a crystallographically symmetry-imposed torsion angle of 180° around the E-isomeric vinyl linkage, and a narrow range of Csp^2^–Csp^2^ bond lengths (1.328(5)–1.455(5) Å) consistent with a high degree of π-conjugation. In addition, nearly orthogonal orientations of the hexyl side chains effectively prevent π-stacking in the solid-state.

The Raman spectra of π-conjugated molecules provide interesting insights of the π-electron delocalization in the ground electronic state.^9^ These spectra for the five molecules in solid state are displayed in Fig. 1(e) as well. Two important bands are noticed: one around 1600 cm^–1^ and another one around 1400 cm^–1^. The 1600 cm^–1^ band is associated with the stretching vibration of the central C=C, or *ν*(C=C), which displays a progressive frequency downshift of 12 cm^–1^ from **2CPDTV** → **6CPDTV** (8 cm^–1^ on **2CPDTV** → **3CPDTV**) due to a progressive enlargement of the C=C vinylene bonds upon increasing the chain length. This is a clear fingerprint of the increasing of π-electron conjugation. The most intense Raman band of the spectra appears around 1400 cm^–1^ and correspond to a *ν*(C=C) of the thiophene rings of the **CPDT** unit. This band also experiences a frequency downshift with increasing oligomer size and this trend is consistent with other π-conjugated systems (see Fig. S50 and S51†).

### AFM morphology

**Fig. 2:**
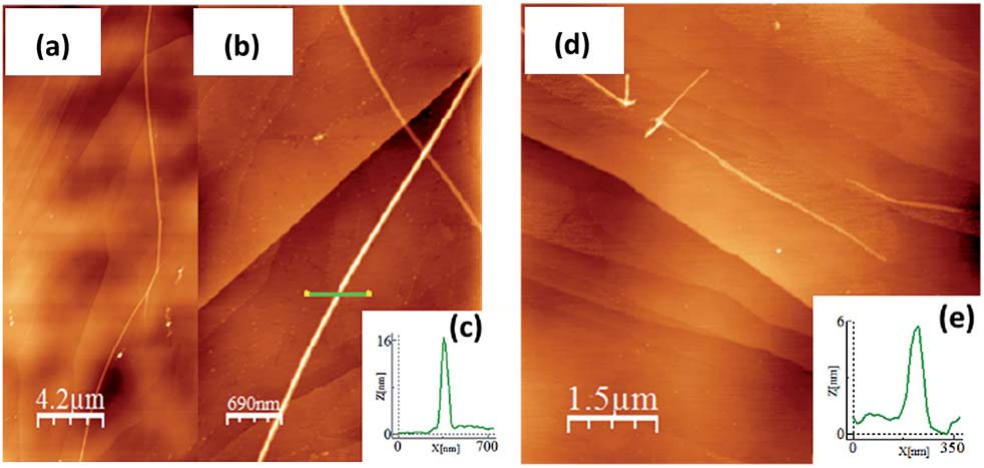
(a) AFM topographic image of **2CPDTV** on HOPG. (b) An image and a zoom (c) of the fiber structure and its height profile are respectively shown. The length and height of the different ***n*CPDTV** change on surface. (d) Topographic image of **4CPDTV** on HOPG shows the formation of fibers with a lower length and height (e) than the observed in **2CPDTV**.

A study of the surface behavior of the different ***n*CPDTVs** oligomers was performed using AFM. For these experiments CH_2_Cl_2_ solutions of the **2–6CPDTV** oligomers were drop-casted on HOPG. In all cases, the AFM topography images show the formation of long chains with heterogeneous height (Fig. 2, S52 and S53†).

For most of the ***n*CPDTV** oligomers, the formation of fibers was not observed when cast on other substrates like mica or SiO_2_. Only **5CPDTV** shows the formation of micrometric (height and length) fibers on mica. This self-assembly is driven by π–π intermolecular interactions and by the interplay with the HOPG. In this case the nearly co-planar structure of the π-conjugated core offers a large available π-surface which promotes the formation of these long fibers.

### Unsubstituted ***n*CPDTVs**: optical and structural properties in the oxidized state

Table 1 summarizes the oxidation potential values of the ***n*CPDTVs** (Fig. S54–S63†). While in **2CPDTV** and **3CPDTV** the formation of the radical cation and dication occurs in separated processes, in **4CPDTV** and longer molecules the two anodic waves coalesce in one 2-electron process to yield the dication. The potential for these processes progressively decreases with the increasing molecular length indicative of enlarging π-conjugation. For **5CPDTV** and **6CPDTV**, higher oxidation states are also accessible.

**Table 1:** Electrochemical data (V *vs.* Fc+/Fc) for the oxidation processes of the ***n*CPDTV** series measured by OSWV in *o*-DCB/CH3CN (4 : 1) solutions with 0.1 M TBAClO_4_

***n*CPDTV**	Eox1	Eox2	Eox3	Eox4	Eox5	Eox6
**2**	0.120	0.290	—	—	—	—
**3**	–0.020	0.074	0.69	0.95	—	—
**4**	–0.070^*a*^	0.42	1.23	—	—	—
**5**	–0.10^*a*^	0.22	0.31	0.73	1.01	1.30
**6**	–0.12^*a*^	0.16	0.51	0.70	1.18	1.30

^*a*^First and second monoelectronic oxidation processes could not be distinguished.

### Radical cations

**Fig. 3:**
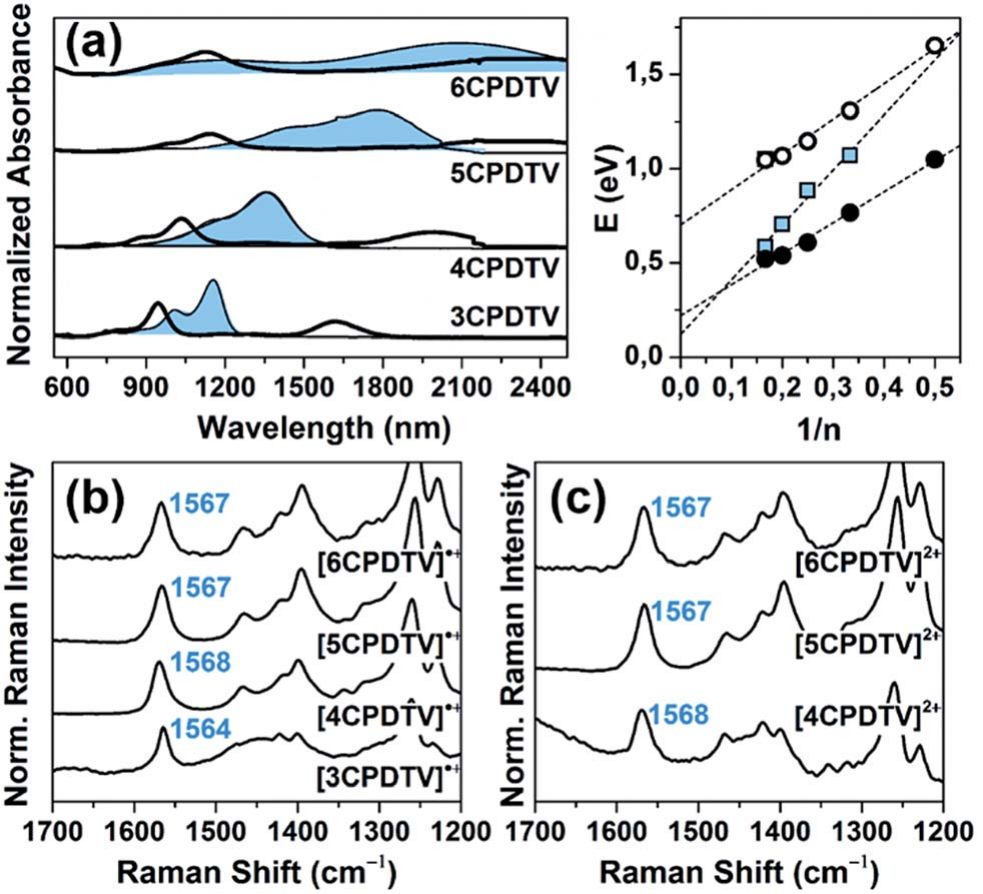
(a) Spectroelectrochemically obtained UV-Vis-NIR absorption spectra of the radical cations and dications (shaded bands) of ***n*CPDTV** compounds (*n* = 3–6) in CH_2_Cl_2_ solutions, together with the plot of the absorption maxima (in eV) *versus* the reciprocal number of **CPDTV** units with their linear regression fitting for **[*n*CPDTV]˙^+^**: SOMO → LUMO transition (white circles, *y* = 1.87*x* + 0.70), HOMO → SOMO transition (black circles, *y* = 1.65*x* + 0.22) and **[*n*CPDTV]^2+^** (blue squares, *y* = 2.91*x* + 0.12). (b) FT-Raman spectra (*λ* = 1064 nm) of **[*n*CPDTV]˙^+^** (*n* = 3–6). (c) FT-Raman spectra (*λ* = 1064 nm) of **[*n*CPDTV]^2+^** (*n* = 4–6), in both cases: *ν*(C=C)_vinyl_ in blue.

The oxidation processes have been analyzed by *in situ* UV-Vis-NIR absorption spectroelectrochemistry and the spectra for the radical cations of all compounds are presented in Fig. 3(a). These spectra are typical of open-shell cations with two well- defined absorption bands in the Vis-NIR region (see Section 9 of ESI and Fig. S64–S68 and Tables S2–S6† for further details). For instance, in **3CPDTV˙^+^**, the band at 948 nm is due to the transition from the SOMO to the LUMO of the open-shell system while that at 1618 nm corresponds to the excitation from the HOMO to the SOMO (see last Section). The *λ*_max_ of these absorptions are represented in Fig. 3(a) as a function of 1/*n* where both bands fit linearly, indicating that their spectral properties (*e.g.*, wavelength, intensity, *etc.*) are the result of the effective extension of the π-electron structure in the radical cation state along the increasing molecular platforms (not the whole structure) when enlarging the molecular size.

The Raman spectra of the ***n*CPDTV˙^+^** (*n* = 3–6, that of *n* = 2 could not be recorded) are shown in Fig. 3(b) (see Fig. S69† for the main vibrational eigenvectors) which are characterized by a new frequency position for the vinylene *ν*(C=C) band, appearing by nearly 30 cm^–1^ lower than in the neutral case. This is a clear indication that the first one-electron oxidation greatly affects the central vinylene, due to the partial C=C → C–C transformation, or weakening, of the double bond. Changes in the frequencies of the thiophene bands are much smaller than those in the vinylene stretching band. Nonetheless, by progressing to the radical cations of the longer oligomers, the frequency of the vinylene Raman band upshifts from 1564 to 1568 cm^–1^ on **3CPDTV˙^+^** → **4CPDTV˙^+^** due to the further dissemination of the polaronic structural defect in a larger π-conjugated system. This effect reaches a plateau on **5CPDTV˙^+^**/**6CPDTV˙^+^** for which the deformation is almost constant.

### Dications

Additional one-electron oxidation of the radical cation produces the progressive disappearance of the pair of radical cation bands and the continuous rise of a strong absorption at wavelengths in between the two of the radical cations. The wavelength position of the *λ*_max_*versus* 1/*n* (Fig. 3(a)) also follows a linear correlation, meaning that the dication is delocalized over larger and larger molecular dimensions. A greater slope than in the radical cations is due, in part, to the electrostatic repulsion between the charges of the same sign which pushes these further apart and contributes to more accentuated delocalization of the defects. These dication absorption bands have a high energy vibronic component which is present in all spectra and also in those of the radical cations. This reveals the stiffening of the backbone upon oxidation and is consistent with the rigidification in the vinylene bridges, as a result of the C–C=C–C → C=C–C=C evolution.

In addition, a new band appears at higher energies together with the strongest one just described. This is clearly seen in the case of **6CPDTV^2+^** where, as well as the strongest band at 2121 nm, another medium intensity band at 1178 nm is detected. This double-band pattern in the dications of oligothiophenes has been associated with the presence of polaron-pair defects, where the dication, instead of being embedded in one single structural bipolaron deformation, prefers to generate two radical cation structural defects by putting away the two charges.^10^,^11^ In order to be effective, having long molecular π-platforms, that largely minimize the repulsion, is required. This is mainly seen in oligothiophenes longer than octamers.^10^,^11^ Clearly, **6CPDTV^2+^**, thanks to its size and structure, is a candidate to stabilize the dicationic structure in a polaron-pair format rather than in a bipolaron, which is preferentially the case of the smaller oligomers, such as **3CPDTV^2+^**.

The Raman spectra of the ***n*CPDTV^2+^** series (*n* = 4–6) are displayed in Fig. 3(c). The diagnostic vinylene *ν*(C=C) band appears at 1568 cm^–1^ in **4CPDTV^2+^** and displays the same frequency than the radical cation (**4CPDTV˙^+^** at 1568 cm^–1^) suggesting that the perturbation of the central vinylene is similar in the dication than in the cation. This is in consonance with the repulsion effect between the charges, which pushes these towards the terminal rings. In the two longer dications, **5CPDTV^2+^** and **6CPDTV^2+^**, the vinylene *ν*(C=C) bands are at almost the same values than in **4CPDTV^2+^** (1567 cm^–1^ in both cases). The invariance of these Raman frequencies, among dications and also from the radical cations to dications of the longer oligomers, indicates that in the monovalent species, the structural alteration affects the central part of the molecule while in the divalent species these structural modifications do not happen in the center but are placed at the terminal rings leaving the middle site with a partial affectation (similar to the radical cation). This spectroscopic-structural interpretation is in agreement with the formation of a polaron-pair structure in longer dications. In short, since the polaron-pair is composed of two separated radical cations, it is expected that these show similarities with the radical cations *per se*.^11^

**Fig. 4:**
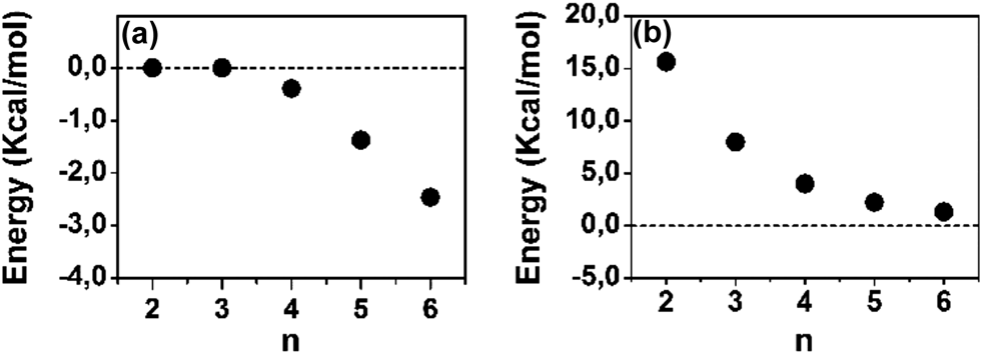
DFT (U)B3LYP/6-31G** energy differences for **[*n*CPDTV]^2+^**: (a) singlet open-shell minus the singlet closed-shell energies and (b) triplet minus singlet open-shell energies.

A polaron-pair charge defect can be described as an open-shell diradical dication and can be compared, by quantum chemistry, to the bipolaron charge defect, which corresponds only to one structural deformation and therefore it is a closed-shell system. Thus, we have calculated at the DFT/(U)B3LYP/6-31G** level the energy difference between the closed- and open-shell structures, both as singlet states, as a function of the oligomer size for the dications and these differences, Δ*E*_OC_, are represented in Fig. 4(a). We observe that for **2CPDTV^2+^** and **3CPDTV^2+^**, Δ*E*_OC_ = 0 kcal mol^–1^, indicating that the stable form is the closed-shell configuration, or bipolaron structure. From **4CPDTV^2+^**, Δ*E*_OC_ starts to be negative suggesting the preference for the open-shell form or polaron-pair – a preference that is more accentuated in the longer compounds.

For these dications, we have also calculated the difference between the singlet ground electronic state and the first energy lying triplet. Following the tendency of Δ*E*_OC_, the triplet state is progressively getting closer to the singlet ground electronic state due to the transformation of this into an open-shell diradical. The nature and consequences on the closed-shell to open-shell transition from the data provided by quantum chemical calculations in correlation with the experimental spectroscopic data in the case of **6CPDTV^2+^** is now discussed in more detail.

In Table 2 the canonical forms representing the closed- and open-shell structures (of **6CPDTV˙^+^** and of **6CPDTV^2+^** from the B3LYP/6-31G** optimized structures) are represented which respectively depict a full quinoidal structure in the middle thiophenes for the radical cation and a pseudo-aromatic form for these also in the middle for the dication. In the dication, a full quinoidal structure can be formulated for the thiophene ring (closed-shell structure). However, this is unstable at the UB3LYP/6-31G** level and evolves by the rupture of one bond of this quinoidal structure giving way to the diradical species stabilized by the aromaticity recovery in the central thiophenes (aromatization of this central part is the driving force for the closed- to open-shell transition).^12^–^14^ In order to probe this, we have calculated the NICS values for the rings of these oxidized species of **6CPDTV **(summarized in Table 2). We see that the NICS values for the neutral species vary between –7.0 and –6.0 ppm, therefore characteristics of the thiophene aromatic rings. The formation of the radical cation produces the transformation of the central rings into quinoidal such as seen from their NICS values of –5.5 ppm. The dication, however, discloses the recovery of the NICS values in the aromatic interval around –6.0 ppm, in agreement with the formation of an open-shell diradical, which aromatizes the central part of the molecule between the radicaloid centers.

**Table 2:** DFT (U)B3LYP/6-311+G(2d,p) NICS (1) *zz* values of neutral, radical cation, and dication of **6CPDTV** as well as the chemical structure with the numbered rings, followed by the most representative canonical forms for the neutral, radical cation and dication of **6CPDTV** (substituents are omitted)

Ring	**6CPDTV**	**6CPDTV˙^+^**	**6CPDTV^2+^**
**6**	–7.983	–7.953	–8.0539
**5**	–6.184	–6.128	–6.1503
**4**	–6.141	–5.792	–5.7485
**3**	–6.093	–5.707	–5.7141
**2**	–6.058	–5.508	–6.0197
**1**	–6.043	–5.482	–6.1368
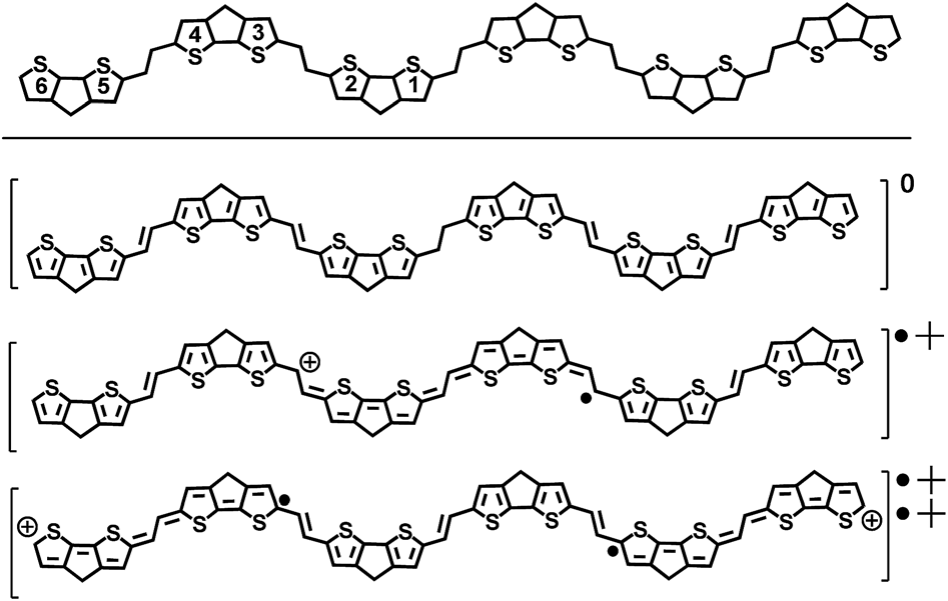			

### Aromatic **2CPDTV** with bis(triphenylamine) substitution

**Scheme 2:**
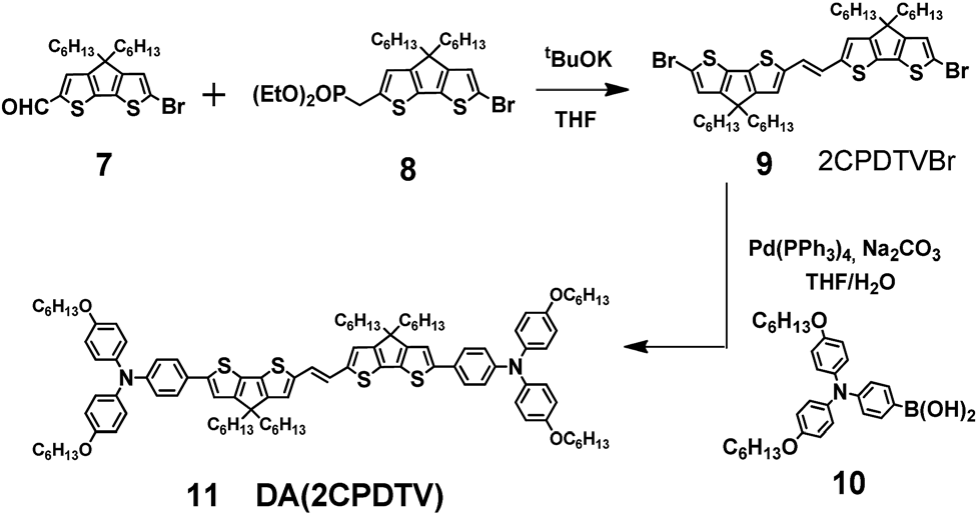
Synthesis of **DA(2CPDTV)**.

Scheme 2 depicts the chemical structure of the aromatic derivative, **DA(2CPDTV)**, disubstituted with triphenylamine groups at the terminal positions of the cyclopentadithiophenes to study the role of the ***n*CPDTV** bridge upon charge transfer from the donor amine groups. Compound **9** was prepared in 75% yield by the Horner–Wadsworth–Emmons reaction between **7** and **8**.^15^ Next, the Suzuki–Miyaura cross-coupling reaction between **9** and **10** afforded **DA(2CPDTV)** in 66% yield (see Section 2 of ESI file for further details†).

**Fig. 5:**
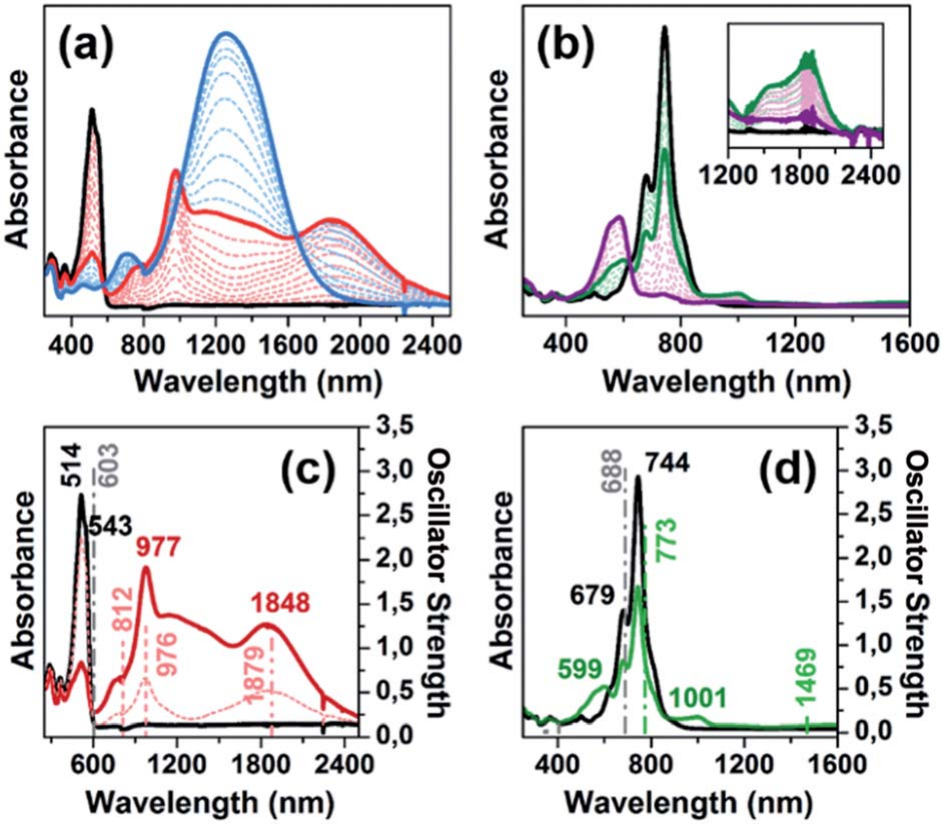
Top: UV-Vis-NIR absorption spectra of: (a) **DA(2CPDTV)** electrochemically oxidized species; and (b) **Q(2CPDTV)** electrochemically reduced species. Bottom: UV-Vis-NIR absorption spectra of: (c) neutral and radical cation of **DA(2CPDTV)** and (d) neutral and radical anion of **Q(2CPDTV)** together with their oscillator strengths computed at the TD(U)DFT levels. Color codes are neutral in black, radical cation in red, dication in blue, radical anion in green and dianion in purple.

One-electron oxidation of **DA(2CPDTV)** yields the radical cation, **DA(2CPDTV)˙^+^**, which is characterized by the appearance of two very intense bands at the expense of the bands of the neutral at 514 nm (Fig. 5(a)). These two new bands are largely red-shifted to 977 and 1848 nm compared with the radical cations of **2CPDTV˙^+^** and **3CPDTV˙^+^** and very close to those of **4CPDTV˙^+^** indicating the role of the triphenylamines in extending π-conjugation.

The lowest-energy lying band at 1848 nm is predicted by TDDFT/(U)B3LP/6-31G* in **DA(2CPDTV)˙^+^** at 1879 nm emerging from a HOMO → SOMO excitation where the HOMO is mainly placed at the terminal phenyl amino rings, whereas the SOMO shifts the electron density mostly on the center of the dithiophene-vinylene core. The large red-shift on **2CPDTV˙^+^** → **DA(2CPDTV)˙^+^** is caused by the charge resonance of the positive charge of the radical cation between the two nitrogens of the triphenyl amines through the **2CPDTV** bridge during the photoinduced absorption. However, in **2CPDTV˙^+^** the charge and its corresponding excitations are placed in the center of the molecule. This band is the so-called intramolecular charge transfer band or ICT band. From its spectrum, one can infer about the type of mixed valence system involved, either class II or class III. For class III, the band might show a vibronic component and a cut-off on the low-energy side, while class II systems exhibit Gaussian-shape bands without fine structure.^16^,^17^ From this point of view, **DA(2CPDTV)˙^+^** could be described as a class II mixed valence compound where the positive charge in the ground electronic state is mainly residing in one nitrogen partially stabilized towards the bridge.

**Scheme 3:**
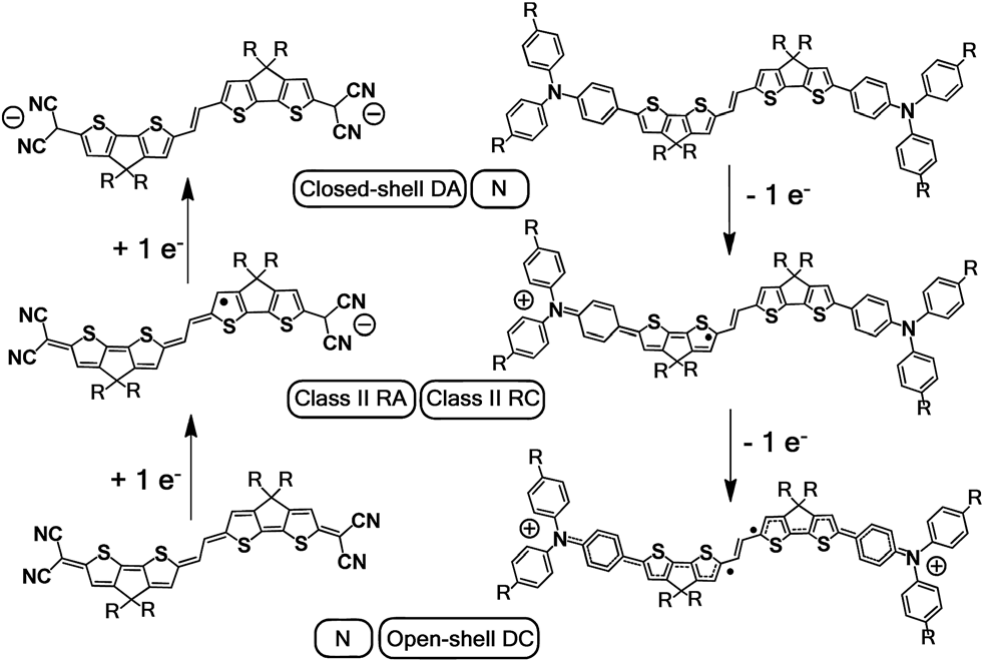
Charged species involved in the oxidation and reduction processes of **DA(2CPDTV)** and **Q(2CPDTV)**. DA: dianion, N: neutral, RA: radical anion, RC: radical cation, DC: dication.

Post-oxidation of **DA(2CPDTV)˙^+^** yields the dication species, **DA(2CPDTV)^2+^**, whose electronic absorption spectrum in Fig. 5(a) is featured by a strong band at 1254 nm which is particularly broad and is accompanied by a new band at 704 nm. These two spectral properties highlight the resemblance of this spectrum with that of **6CPDTV^2+^**, due to an open-shell polaron-pair dication. In this case, the great resonance stabilization of one charge in each nitrogen atom, together with the aromatization of the central dithiophenes, might justify the formation of this diradical species in this rather small compound^18^–^20^ (see Scheme 3).

### Quinoidal **2CPDTV** with bis(dicyanoquinodimethane) substitution

**Scheme 4:**
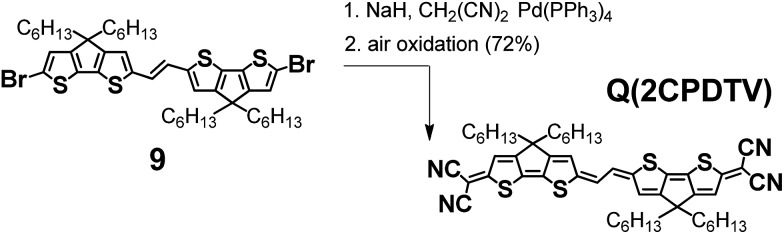
Synthesis of **Q(2CPDTV)**.

Scheme 4 depicts the chemical structure of the quinoidal derivative of ***n*CPDTV**, **Q(2CPDTV)**, together with its synthesis (see Section 2 of ESI file for further details†). Introduction of the dicyanomethylene moiety to **2CPDTVBr** and conversion to its quinoidal form **Q(2CPDTV)** was accomplished using the well-established procedure: (i) Pd-catalyzed Takahashi coupling^21^ of the malononitrile anion with the aromatic halide; (ii) further deprotonation with NaH to afford a dianion intermediate; and (iii) oxidation of the dianion for conversion of the aromatic oligomer to the quinoidal form. For the final step, air oxidation was employed which led to the formation of **Q(2CPDTV)** in good yield.

Oligomers of this type are of interest as tetracyanoquinodimethanes oligothiophenes are being extensively exploited in the field of organic conjugated molecules.^22^–^25^

**Fig. 6:**
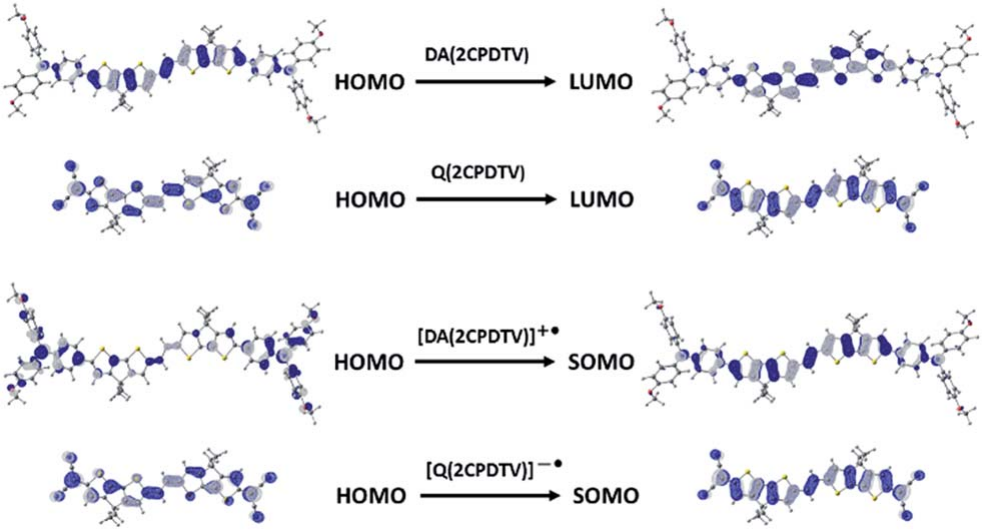
Wavefunction topologies of the relevant orbitals involved in the lowest-energy lying bands of the optical spectra of the neutral (top) and of the radical cation and radical anion (bottom) of **DA(2CPDTV)** and **Q(2CPDTV)** respectively.

Fig. 6 displays the electrochemical response and optical absorption spectrum of **Q(2CPDTV)** in the neutral state; the absorption spectra of the two relevant reduced states by spectroelectrochemistry are shown in Fig. 5(b) and (d). The cyclic voltammetry of **Q(2CPDTV)**, in contrast with **2CPDTV**, shows a two-electron reduction at –0.12 V and two one-electron oxidations displaced at higher anodic potential in comparison to the aromatic analogues, at 1.10 and 1.38 V. Neutral **Q(2CPDTV)** shows one very strong absorption band at 744 nm with a vibronic satellite at 679 nm (ref. 26) (Fig. 5(b)). This electronic absorption spectrum is clearly similar to that of the dications of ***n*CPDTV** which exhibit the same pattern of bands (for instance in **3CPDTV^2+^**has a strong peak at 1157 nm and a satellite at 1012 nm) although in the case of the charged species the absorptions are displaced at longer wavelengths indicative of the smaller HOMO–LUMO gaps. This is in accordance with a well defined closed-shell thienoquinoidal structure for **Q(2CPDTV)** whereas the dications display a transition between closed to open-shell structure such as discussed before.

The absorption spectra of **Q(2CPDTV)** as a function of the temperature in solution are shown in Fig. 6(b). Upon cooling, the progressive appearance of a new band with peak maxima at 918 nm (satellite at 798 nm) is displayed. This appears through a well resolved isosbestic point from the spectrum at room temperature, revealing the different nature of the new low temperature species of **Q(2CPDTV)**.^27^ This behavior is distinctively different from that of the aromatic ***n*CPDTV** and has been also described in other quinoidal oligothiophenes resulting from J-aggregates.^28^ If aggregation takes place, it certainly overpasses the severe steric crowding by the hexyl groups at the dithiophene units revealing that the planar π-surface of the molecules promotes efficient intermolecular contacts such as in the case of the formation of fibers in the neutral forms in HOPG surfaces by AFM.

**Fig. 7:**
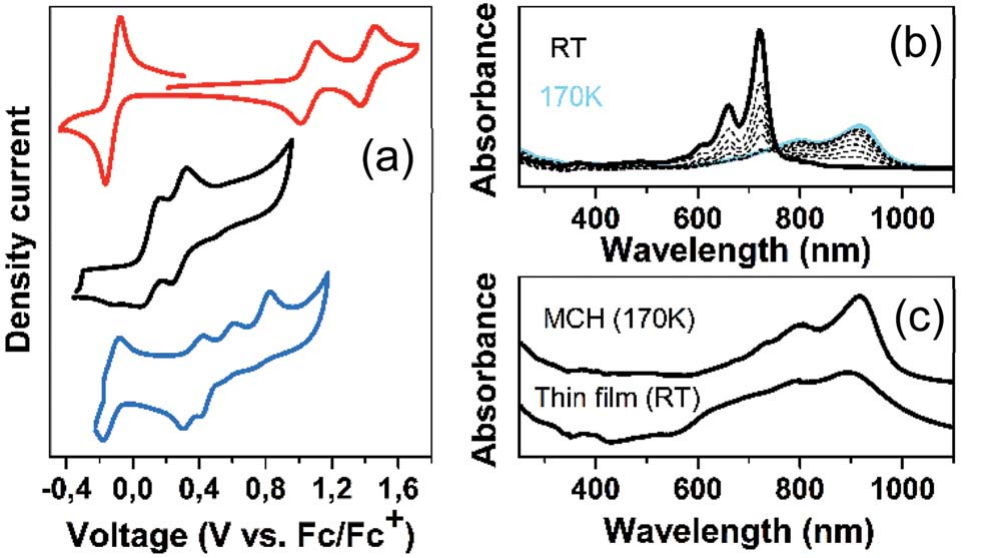
(a) Cyclic voltammetries of **2CPDTV** (black), **Q(2CPDTV)** (red) and **DA(2CPDTV)** (blue) in CH_2_Cl_2_ solution at room temperature. (b) Electronic absorption spectra of **Q(2CPDTV)** in methylcyclohexane (MCH) as a function of the temperature; (c) electronic absorption spectrum in solid state of **Q(2CPDTV)** by drop cast at room temperature together with that in solution at 170 K.

UV-Vis-NIR absorption spectroelectrochemical experiments of **Q(2CPDTV)** have been carried out at reducing potentials. One-electron reduction gives rise to the progressive disappearance of the neutral band and the emergence of two new weak features at 1001 and 1889 nm (vibronic shoulder at 1585 nm). TDDFT/(U)B3LP/6-31G* of **Q(2CPDTV)˙^–^** (Fig. 5(d)) allows the assignment of these absorptions and, in particular, the very weak band at 1889 nm is predicted at 1469 nm and is due to a double HOMO → SOMO + SOMO → LUMO promotion involving orbitals that in all cases extend or affect the whole molecule (*i.e.*, external dicyano-methylene and the central core). Furthermore, this band is predicted to have a very low oscillator strength which is consistent with its weak absorbance in the experiment. The lowest-energy absorption band of **Q(2CPDTV)˙^–^** resembles very much the ICT band of **DA(2CPDTV)˙^+^** either in their wavelength positions (1848 and 1889 nm respectively) or in their shapes (both are Gaussian type bands), indicating that the radical anion is also a class II mixed valence compound in which the additional negative charge resides in the dicyano-methylene partially invading the bridge (see Scheme 3). Both bands are completely different regarding the whole absorbance: while the cation band is strong, in the anion is very weak and is probably caused by the different electron density pattern of the involved orbitals. In **DA(2CPDTV)˙^+^**, the HOMO → SOMO excitation represents an electron density displacement from the external sites to the internal core, producing a large variation of the dipolar momentum (and of the transition momentum). In **Q(2CPDTV)˙^–^**, the same excitation invokes a reorganization of the electron density in the whole molecule with a much smaller impact in the variation of the dipolar momentum of the transition (see Fig. 7 for the orbital topologies). Post-reduction of **Q(2CPDTV)˙^–^** gives rise to the dianion species which is characterized by a band at 586 nm and is typical of those of the aromatic ***n*CPDTV** revealing the full aromatization of the bithiophene moieties upon double charging (Fig. 5(b) and (d)).

**Fig. 8:**
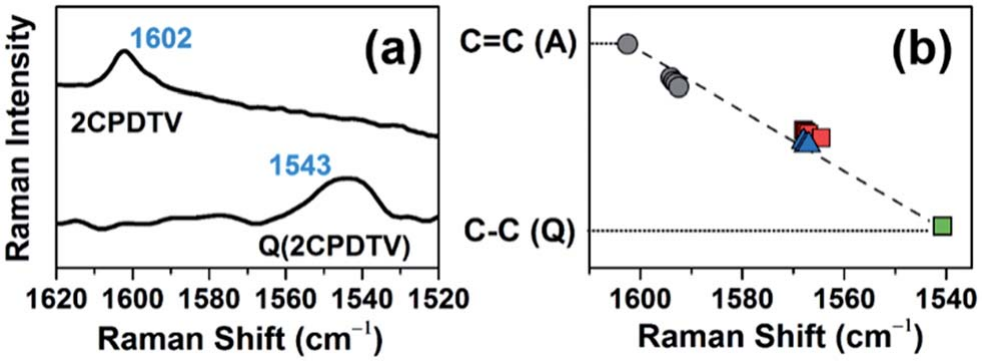
(a) Raman spectra of neutral **Q(2CPDTV)** and **2CPDTV**. (b) Linear fit of all Raman wavenumbers of the *ν*(C=C) bands of the neutral (black filled circles) and of the oxidized ***n*CPDTV** compounds, blue triangles for the radical cations and red squares for the dications. **Q(2CPDTV)** is shown as a green square. A: aromatic, Q: quinoidal.

The Raman spectrum of **Q(2CPDTV)** in Fig. 8(a) is typical of a bipolaronic form with a quinoid structure for the thiophene and an inversion of the pattern from double to single in the central CC bond. This is reflected in its vibrational Raman spectrum with the vinylene *ν*(C=C) band at 1543 cm^–1^, largely downshifted relative to the values in ***n*CPDTV** at 1590 cm^–1^, as a result of the weakening of the vinylene bond upon tetracyano substitution (Fig. S69†). Therefore, the *ν*(C=C) frequency at 1602 cm^–1^ in neutral **2CPDTV** and at 1543 cm^–1^ in **Q(2CPDTV)** might represent the limits of a thienoquinoidization scale of the **CPDTV** π-conjugated structure, the former being the frequency of the full aromatic structure (0% quinoidal, A in Fig. 8(b)) and the latter that of the full thienoquinoidal form (100% quinoidal, Q in Fig. 8(b)). In this representation, the *ν*(C=C) bands at 1564 cm^–1^ for **3CPDTV˙^+^** yields a quinoidization extension of 64% in line with the aromatic → quinoidal transitional character of the radical cations. For the dications, the quinoidal percentage is almost identical (band at 1568 cm^–1^ in **4CPDTV^2+^** results in 57% quinoidal) although the structure is not the same since the structural alteration is distributed in a polaron-pair form. Fig. 8(b) shows the wavenumber position in the quinoidal scale deduced indirectly by the *ν*(C=C) frequency values positions regarding the values in neutral **Q(CPDTV2)** and **2CPDTV**. These observations show how the radical cations and dications both occupy a middle region between the full aromatic and full quinoidal points revealing the transitional character of their structures. Nevertheless, the neutral oligomers logically reside in a part of the quinoidal scale, closer to the aromatic fingerprint.

## Conclusions

A new series of π-conjugated oligomers based on the 4,4′ dihexyl-4*H*-cyclopenta[2,1-*b*:3,4-*b*′]dithiophene vinylene repeating units has been prepared and characterized by spectroscopic and modelling methods. The neutral compounds display sizeable π-conjugation with increasing molecular size and a longer mean conjugation length than their oligothiophene and oligothiophene vinylenes homologues. Consequently the new compounds show rich redox chemistry with the stabilization of polycationic states of which the radical cations and dications are strong NIR absorbers – the latter displaying singlet diradicaloid states due to the gaining or recovery of aromaticity in the thiophene rings from the unstable quinoidal bipolaronic structures. Radical cations are described as having a quinoidal structure placed in the molecular center and flanked by two aromatic structures in the terminal parts. Conversely, the dications, as a result of their diradical character, display the reversed trend with aromatic segments in the molecular center surrounded by two quinoidal external moieties.

To better investigate the potential charge transport properties as channels for either positive (*i.e.*, hole) or negative (*i.e.*, electron) transport, two more derivatives were prepared. The **CPDTV** cores were substituted, either with electron donor triphenyl amino groups or with bis(dicyano-methylene) caps, enforcing a quinoidal structure in the dithiophene-vinylene bridge. The positive charge of the radical cation of the triarylamine compound is localized in the amine groups, and behave as class II mixed valence systems. The radical anion of the tetracyano compound, meanwhile has the same confinement effect of the charge in the environment of the electron accepting groups. The dication and dianion of these two compounds are open-shell biradical or polaron pair and closed-shell or bipolaronic structures respectively.

The rich redox chemistry using these bridging groups, together with the adequate substitution of these cores, produce π-conjugated compounds with a variety of structures and properties. They can be exploited to function as materials for organic electronics but these materials are also of importance to gain insights into the intricate electronic structure of highly π-electron conjugated molecules.

## Conflicts of interest

There are no conflicts to declare.

## Supplementary Material

Supplementary information

Crystal structure data
