# Supplementary material for: Oligomers of cyclopentadithiophene-vinylene in aromatic and quinoidal versions and redox species with intermediate forms
Source: Chem Sci. 2017 Sep 27;8(12):8106–14. doi: 10.1039/c7sc02756g (PMC5855126; doi:10.1039/c7sc02756g)
Supplement: Supplementary file 1 [file SC-008-C7SC02756G-s001.pdf]

Electronic Supplementary Material (ESI) for Chemical Science.
This journal is © The Royal Society of Chemistry 2017

Electronic supplementary information for Oligomers of Cyclopentadithiophene-Vinylene in Aromatic and Quinoidal Versions and Redox Species with Intermediate Forms

Paula Mayorga Burrezo[1,2†], Rocío Domínguez[3†], José L. Zafra[1], Ted M. Pappenfus[4], Pilar de la Cruz[3], Lorena Welte[3], Daron E. Janzen[5], Juan T. López Navarrete[1], Fernando Langa*[,3], Juan Casado*[,1]

[1]Department of Physical Chemistry, University of Málaga, Campus de Teatinos s/n, Málaga, 29071, Spain

[2] Current adress: Department of Molecular Nanoscience and Organic Materials, Institut de Ciència de Materials de Barcelona (ICMAB-CSIC), Campus Universitari de Bellaterra, E-08193, Cerdanyola, Barcelona, Spain.

[3]University of Castilla-La Mancha, Institute of Nanoscience, Nanotechnology and Molecular Materials (INAMOL), Campus de la Fábrica de Armas, 45071-Toledo, Spain

[4]Division of Science and Mathematics, University of Minnesota, Morris, Minnesota 56267, United States

[5]Department of Chemistry and Biochemistry, St. Catherine University, St. Paul, Minnesota 55105, United States

Author Information: † These authors contributed equally to this work.

Corresponding Author: Fernando.Langa@uclm.es; casado@uma.es

Content

1. General remarks: Materials and Methods 2
2. Synthetic details and analytical data 4
3. ^{1}H NMR, ^{13}C NMR, MS and IR spectra 12
4. Thermogravimetric analyses (TGA) 35
5. X-ray Crystallography of 9 (2CPDTV-Br) 36
6. Theoretical analysis of Raman spectra of nCPDTV. 39
7. Atomic Force Microscopy Morphology 40
8. Ciclic Voltammetry (CV) and Osteryoung Square Wave Voltammetry (OSWV) spectra 42
9. Generation of positive charged species in nCPDTV 47
10. Theoretical evolution of ν(C=C)vinyl in nCPDTV-T charged species. 50

1. General remarks: Materials and Methods

Synthetic procedures were carried out under inert argon atmosphere and in dry solvent unless otherwise noted. All reagents and solvents were reagent grade and were used without further purification. Chromatographic purifications were performed using silica gel 60 SDS (particle size 0.040-0.063 mm). Analytical thin-layer chromatography was performed using Merck TLC silica gel 60 F254. 1H NMR spectra were obtained on Bruker TopSpin AV-400 (400 MHz) spectrometer. Chemical shifts are reported in parts per million (ppm) relative to the solvent residual peak (CDCl3, 7.27 ppm). 13C NMR chemical shifts are reported relative to the solvent residual peak (CDCl3, 77.00 ppm). UV-Vis measurements were carried out on a Shimadzu UV 3600 spectrophotometer. For extinction coefficient determination, solutions of different concentration were prepared in CH2Cl2, HPLC grade, with absorption between 0.1-1 of absorbance using a 1 cm UV cuvette. Mass spectra (MALDI-TOF) were recorded on a VOYAGER DETM STR mass spectrometer using dithranol as matrix. Melting points are uncorrected.

Electrochemical experiments have been conducted in o-DCB/acetonitrile (4:1) at room temperature by using 0.1 M tetrabutyl ammonium perchlorate, Bu4NClO4, as the supporting electrolyte. A glassy carbon was used as the working electrode and a Pt wire as the auxiliary electrode against a pseudo-reference electrode of Ag/0.001M AgNO3 0.1M Bu4NClO4. All redox potentials provided are referred to the Fc/Fc+ pair. In situ UV-Vis-NIR spectroelectrochemical studies were conducted on the Cary 5000 spectrophotometer. A C3 epsilon potentiostat from BASi was used for the electrolysis using a thin layer cell from a demountable omni cell from Specac. In this cell a three electrodes system was coupled to conduct in situ spectroelectrochemistry. A Pt gauze was used as the working electrode, a Pt wire was used as the counter electrode, and an Ag wire was used as the pseudo-reference electrode. The spectra were collected a constant potential electrolysis and the potentials were changed in interval of 15 mV. The electrochemical medium used was 0.1 M TBA-PF6 in fresh distilled CH2Cl2, at room temperature with sample concentrations of 10^{-3} M.

Oxidized species were generated in CH2Cl2 solutions by progressive addition of FeCl3. UV-Vis-NIR spectrochemical measuremens were conducted in the Cary 5000 spectrophotometer whereas Raman measurements are taken with the spectrometers described below.

Thermogravimetric analyses were performed using a TGA/DSC Linea Excellent instrument by Mettler-Toledo and collected under inert atmosphere of nitrogen with a scan rate of 10ºC min^{-1}. The weight changes were recorded as a function of temperature. Differential scanning calorimetry analyses were performed by a DSC Linea Excellent instrument by Mettler-Toledo, and collected under inert atmosphere of nitrogen at a heating rate of 20 K/min and a cooling rate of 10 K/min.

AFM images were recorded in tapping mode and in air conditions using a Multimode 8 system with a NanoScope V controller from Bruker. RTESP-300 Bruker cantilevers with a *resonance frequency* of *300 kHz* and a nominal force constant of 40 Nm-1 were used. The samples investigated by AFM were prepared by drop-casting on HOPG and mica substrates of 20 µL of 10-6 mg/mL dichloromethane bis-pyrazolinofullerene solutions.

Absorption and emission spectroscopy. Absorption spectra were recorded with a Cary 5000 spectrophotometer from Varian operating in a maximal 175–3300 nm range. Emission and excitation spectra were measured using a spectrofluorometer from Edinburgh Analytical Instrument (FLS920P) equipped with a pulsed xenon flash-lamp, Xe900, of 400 mW. 2Me-THF solutions were prepared to obtain the spectra at different temperatures from room conditions to 100 K in a cryostat OPTISTAT from Oxford instruments.

The 1064 nm FT–Raman spectra were obtained with an FT–Raman accessory kit (FRA/106–S) of a Bruker Equinox 55 FT–IR interferometer. A continuous–wave Nd–YAG laser working at 1064 nm was employed for excitation. A germanium detector operating at liquid nitrogen temperature was used. Raman scattering radiation was collected in a back–scattering configuration with a standard spectral resolution of 4 cm-1. 1000–3000 scans were averaged for each spectrum. The spectra with excitation wavelengths at 532, 633 and 785 nm were collected by using the 1×1 camera of a Bruker Senterra Raman microscope equipped with a CCD camera operating at -50 ºC by averaging spectra during 50 minutes with a resolution of 3–5 cm-1.

Quantum-chemical calculations were done in the framework of the density functional theory as implemented in the Gaussian´09 package.[1] Simulations were performed in the gas-phase. The B3LYP exchange-correlational functional and the 6-31G** basis set were used in all calculations.[2] [3]30,31 Neutral were treated as closed-shell (RB3LYP

[1] Gaussian 09, M. J. Frisch, *Gaussian 09, Revision A.02*, Gaussian, Inc.: Wallingford CT, 2009.
[2] Becke, A. D. *J. Chem. Phys.* **1993**, *98*, 5648−5652.

procedure); while for radical cations, open-shell systems optimizations were carried out using wave functions spin unrestricted (UB3LYP) for radical-cations. Calculations for dication singlet states were performed using spin-restricted and spin-unrestricted wave functions. Spin-unrestricted singlet states were calculated using the broken symmetry DFT method with the "guess=mix" keyword to generate an appropriate guess for the UDFT calculations. Compounds were first calculated in neutral state as close-shell singlets at the spin-restricted (B3LYP) level, and then were re-optimized as open-shell singlet using a spin-unrestricted broken-symmetry (UB3LYP (BS)) function and as triplet at the spin-unrestricted configuration (UB3LYP). Hexyls substitutions were replaced by methyls to reduce the computational cost. The time-dependent DFT (TD-DFT) approach has been used for the evaluation of, at a minimum, the fifteen lowest-energy vertical electronicexcited states.[4] NICS values have been calculated according to the method of Schleyer.[5]

2. Synthetic details and analytical data

Synthesis of 2:

Under inert atmosphere, 4,4-dihexyl-4*H*-cyclopenta[2,1-*b*:3,4-*b'*]dithiophene-2-carbaldehyde (**1**)[6] (0.98 g, 2.52 mmol) was dissolved in 15 mL of CH_2Cl_2 and 15 mL of MeOH, and $NaBH_4$ (0.19 g, 5.04 mmol) was added. The reaction mixture was stirred 90 min at room temperature. Afterwards, the reaction mixture was poured in water and the organic phase was extracted with dichloromethane. The solid obtained after evaporate the solvent was dissolved in 12 mL of freshly distilled triethylphosphite under Ar atmosphere. Then, $ZnBr_2$ (0.68 g, 3.03 mmol) was added and the reaction solution was stirred over night at room temperature. The crude reaction was quenched by pouring into an ice-water/HCl mixture and the organic layer was extracted with dichloromethane. After removing the solvent under vacuum, the solid was purified by chromatography column (silica gel, hexane:ethyl acetate, 1:1) to yield **2** as a brown oil (1.15 g, 2.32 mmol, 92% yield).

^{1}H-NMR (400 MHz, $CDCl_3$) δ/ppm: 7.12 (d, 1H, ^3J = 4.7 Hz), 6.90 (d, 1H, ^3J = 4.7 Hz), 6.85 (d, 1H, ^{4}J = 3.4 Hz), 4.11-4.03 (m, 4H), 3.38 (d, 2H, ^2J = 20.8 Hz), 1.79 (t, 4H, ^3J =

[3] Francl, M. M.; Pietro, W. J.; Hehre, W. J.; Binkley, J. S.; Gordon, M. S.; Defrees, D. J. J.; Pople, A. *J. Chem. Phys.* **1982**, *77*, 3654–3665.

[4] Runge, E.; Gross, E. K. U. *Phys. Rev. Lett.* **1984**, *52*, 997–1000.

[5] Schleyer, P. v. R.; Maerker, C.; Dransfeld, A.; Jiao, H.; Hommes, N. J. R. v. E. *J. Am. Chem. Soc.* **1996**, *118*, 6317–6318.

[6] Li, R.; Liu, J.; Cai, N.; Zhang, M.; Wang, P. *J. Phys. Chem. B* **2010**, *114*, 4461-4464.

8.1 Hz), 1.27 (t, 6H, ^3J = 7.0 Hz), 1.18-1.10 (m, 12H), 0.91 (m, 4H), 0.82-0.78 (m, 6H); ^{13}C-NMR (100 MHz, $CDCl_3$) δ/ppm: 157.6, 157.5, 157.4, 157.3, 136.5, 136.4, 135.6, 135.5, 131.8, 131.7, 124.3, 122.2, 122.1, 121.5, 62.5, 62.4, 53.6, 37.8, 31.7, 29.7, 28.2, 24.5, 22.6, 16.5, 16.4, 14.0.

Synthesis of 3:[7]

4,4-dihexyl-4*H*-cyclopenta[2,1-*b*:3,4-*b'*]dithiophene (850 mg, 2.45 mmol) reacted with 1.89 mL of DMF (24.5 mmol) and 2.24 mL of $POCl_3$ (24.5 mmol) in 50 mL of dry 1,2-DCE at reflux to afford, after purification by chromatography column in silica gel and hexane:chloroform 2:3 mix as eluent, the pure product **3** as orange solid in 93% of yield (920 mg, 2.28 mmol).
^{1}H-NMR (400 MHz, $CDCl_3$) δ/ppm: 9.91 (s, 2H), 7.63 (s, 2H), 1.93-1.89 (m, 4H), 1.25 (m, 4H), 1.21-1.15 (m, 8H), 0.94 (m, 4H), 0.83-0.80 (m, 6H).

Synthesis of 4:

Under argon atmosphere, to a solution of **2CPDTV** (500 mg, 0.70 mmol) in dry 1,2-DCE (80 mL) was added dry DMF (305.7 mg, 4.18 mmol) and $POCl_3$ (641.4 mg, 4.18 mmol), dropwise. The reaction mixture was stirred overnight at room temperature. Then, the mixture was poured into a saturated sodium acetate aqueous solution and stirred for 1 h. The organic phase was extracted with chloroform, dried over $MgSO_4$ and the solvent was evaporated in vacuum. The solid was purified by chromatography column (silica gel, hexane:$CHCl_3$, 2:3). Compound **4** was obtained as a red solid in 80% of yield (496 mg, 0.64 mmol).
^{1}H-NMR (400 MHz, $CDCl_3$) δ/ppm: 9.84 (s, 2H), 7.56 (s, 2H), 7.09 (s, 2H), 6.96 (s, 2H), 1.90-1.84 (m, 8H), 1.22-1.15(m, 24H), 0.97-0.94(m, 8H), 0.84-0.80 (m, 12H); ^{13}C-NMR (100 MHz, $CDCl_3$) δ/ppm: 182.5, 163.0, 158.3, 147.5, 147.3, 143.6, 135.0, 129.8, 122.0, 121.0, 54.0, 37.7, 31.6, 29.6, 24.6, 22.6, 14.0; FT-IR (KBr) υ/cm^{-1}: 2951, 2928, 2854, 1645, 1496, 1394, 1321, 1225, 1130, 704; UV-Vis (CH_2Cl_2) λ_{max}/nm (log ε): 500 (4.86), 533 (4.82); MS (*m/z*) (MALDI-TOF): calculated $C_{46}H_{60}O_2S_4$: 773.23; found: 772.68 (M $^+$); Melting point: 169 ºC.

Synthesis of 5:

[7] Sorohhov, G.; Yi, C.; Grätzel, M.; Decurtins, S.; Liu, S. –X. *Beilstein J. Org. Chem.* **2015**, *11*, 1052-1059.

Under Ar atmosphere, 0.28 mL of *n*-BuLi (2.5 M solution in hexane, 0.70 mmol) was slowly added to a solution of **3CPDTV** (345 mg, 0.32 mmol) in 80 mL of dry THF at -78ºC. The reaction was stirred for 1 h at -10ºC, cooling again to -78ºC. DMF (0.08 mL, 1.09 mmol) was added, and the reaction mixture was stirred for 2 h at -78ºC and overnight at room temperature. Crude product was quenched with water and extracted with diethyl ether. The organic phase was dried over $MgSO_4$, the solvent was evaporated under vacuum and the obtained solid was purified by chromatography column (silica gel, hexane:chloroform 1:4). Compound **5** was obtained as dark red solid in 72% of yield (260 mg, 0.23 mmol).

^{1}H-NMR (400 MHz, $CDCl_3$) δ/ppm: 9.83 (s, 2H), 7.55 (s, 2H), 7.11 (d, 2H, ^3J = 15.6 Hz), 7.01 (d, 2H, ^{3}J = 15.6 Hz), 6.93 (s, 2H), 6.92 (s, 2H), 1.88-1.86 (m, 12H), 1.26-1.16 (m, 36H), 0.97 (m, 12H), 0.85-0.81 (m, 18H); ^{13}C-NMR (100 MHz, $CDCl_3$) δ/ppm: 182.4, 163.2, 159.3, 158.0, 148.1, 147.8, 143.5, 143.2, 136.6, 134.2, 129.8, 122.7, 121.3, 120.1, 119.8, 53.9, 53.8, 37.8, 37.7, 31.7, 31.6, 29.7, 29.6, 24.6, 22.7, 22.6, 14.1, 14.0; FT-IR (KBr) υ/cm^{-1}: 2954, 2926, 2852, 1655, 1496, 1396, 1223, 1128, 926; UV-Vis (CH_2Cl_2) λ_{max}/nm (log ε): 557 (5.00), 588 (4.97); MS (*m/z*) (MALDI-TOF): calculated $C_{69}H_{90}O_2S_6$: 1142.53; found: 1143.20 (M^{+}). Melting point: 145 ºC.

Synthesis of 6:

Under argon atmosphere, to a solution of **4CPDTV** (300 mg, 0.21 mmol) in dry 1,2-DCE (40 mL) at 0ºC, dry DMF (135.3 mg, 1.85 mmol) and $POCl_3$ (189.2 mg, 1.23 mmol) were added dropwise. The reaction mixture was stirred at 50ºC. After consumption of the starting material (14 h, monitored by TLC), the reaction mixture was cooled to r.t. and stirred in a saturated sodium acetate aqueous solution for 1h. The organic phase was extracted with chloroform and dried over $MgSO_4$. After removing the solvent under reduced pressure, the crude product was purified by chromatography column (silica gel, hexane:$CHCl_3$, 1:4). Compound **6** was obtained as a navy blue solid in 93% of yield (290.1 mg, 0.19 mmol).

^{1}H-NMR (400 MHz, $CDCl_3$) δ/ppm: 9.83 (s, 2H), 7.55 (s, 2H), 7.11 (d, 2H, ^{3}J = 15.6 Hz), 7.02-6.98 (m, 4H), 6.92 (d, 4H, ^{3}J = 4.7 Hz), 6.88 (s, 2H), 1.88-1.84 (m, 16H), 1.26-1.17 (m, 48H), 0.98-0.97 (m, 16H), 0.85-0.82 (m, 24H); ^{13}C-NMR (100 MHz, $CDCl_3$) δ/ppm: 182.4, 163.2, 159.4, 159.0, 157.9, 148.2, 147.9, 144.2, 143.1, 143.0, 136.9, 135.7, 134.1, 129.8, 122.8, 121.4, 120.6, 120.4, 120.0, 119.6, 53.9, 53.8, 37.8, 37.7, 31.7, 31.6, 29.7, 29.6, 24.6, 22.7, 22.6, 14.1, 14.0; FT-IR (KBr) υ/cm^{-1}: 2953, 2926, 2852, 1655, 1496,

1394, 1223, 1128, 926; UV-Vis (CH_2Cl_2) λ_{max}/nm (log ε): 585 (5.14); MS (*m/z*) (MALDI-TOF): calculated $C_{92}H_{120}O_2S_8$: 1514.46; found: 1513.19 (M^+). Melting point: 194 ºC.

Synthesis of 8:

Monoaldehyde **7**[2] (500 mg, 1.10 mmol) reacted with $NaBH_4$ (83.4 mg, 2.20 mmol) in 40 mL of dichloromethane and 40 mL of methanol. After 90 min., the reaction mix was extracted with chloroform, the organic phase was dried over $MgSO_4$ and the solvent was evaporated under reduced pressure. The isolated alcohol was converted into the corresponding phosphonate derivative **8** by a Michaelis-Arbuzov reaction. The corresponding alcohol (451 mg, 0.99 mmol) was dissolved in 15 mL of freshly distilled $P(OEt)_3$ under argon atmosphere, and $ZnBr_2$ (268 mg, 1.19 mmol) was slowly added. The reaction mix was stirred overnight at room temperature. Then, it was quenched by pouring into an ice-water/HCl mixture, and the organic product was extracted with dichloromethane and dried over $MgSO_4$. After removing the solvent under reduced pressure, the residue was purified by chromatography column (silica gel, hexane:ethyl acetate, 1:1) to yield pure product **8** as brown oil in 69% of yield (391 mg, 0.68 mmol).

^{1}H-NMR (400 MHz, $CDCl_3$) δ/ppm: 6.92 (s, 1H), 6.84 (s, 1H), 4.10-4.06 (m, 4H), 3.37 (d, 2H, ^2J = 20.8 Hz), 1.76 (t, 4H, ^3J = 7.8 Hz), 1.28 (t, 6H, ^3J = 6.9 Hz), 1.20-1.12 (m, 12H), 0.90 (m, 4H), 0.84-0.80 (m, 6H); ^{13}C-NMR (100 MHz, $CDCl_3$) δ/ppm: 156.7, 156.6, 155.9, 155.8, 136.8, 135.1, 135.0, 132.4, 132.3, 124.5, 122.1, 122.0, 110.3, 62.6, 62.5, 54.4, 37.7, 31.6, 29.6, 28.2, 24.4, 22.6, 16.4, 16.3, 14.0; FT-IR (ATR) ν/cm^{-1}: 2954, 2928, 2854, 1254, 1049, 1026, 956, 794; MS (*m/z*) (MALDI-TOF): calculated $C_{26}H_{40}BrO_3PS_2$: 574.13; found: 574.21 (M^+).

Synthesis of 9 (2CPDTVBr):

To a solution of **7** (308 mg, 0.68 mmol) and **8** (391 mg, 0.68 mmol) in 45 mL of anhydrous THF under Ar atmosphere, ^{t}BuOK (305 mg, 2.72 mmol) was added. The reaction mix was stirred at room temperature, under darkness and monitored by TLC. After consumption of the monoaldehyde (about 5 h), the reaction mixture was quenched with water and extracted with diethylether. The organic phase was dried over $MgSO_4$, concentrated *in vacuo* and purified by chromatography column (silica gel, hexane). Pure product **9** was achieved in 75% of yield as orange solid (448 mg, 0.51 mmol).

^{1}H-NMR (400 MHz, THF-d_8) δ/ppm: 7.15 (s, 2H), 7.08 (s, 2H), 7.06 (s, 2H), 1.92-1.87 (m, 8H), 1.26-1.20 (m, 24H), 1.01-0.99 (m, 8H), 0.87-0.84 (m, 12H); ^{13}C-NMR (100 MHz, THF-d_8) δ/ppm:157.8, 156.9, 143.9, 137.2, 134.9, 124.9, 120.5, 120.4, 110.8, 54.3, 37.6, 31.6, 29.7, 22.5, 13.4; FT-IR (KBr) ν/cm^{-1}: 2953, 2926, 2854, 1460, 1331, 931, 829, 725, 567; UV-Vis (THF) λ_{max}/nm (log ε): 458 (4.78), 485 (4.71); MS (*m/z*) (MALDI-TOF): calculated $C_{44}H_{58}Br_2S_4$: 872.18; found: 872.40 (M^+). Melting point: 136 ºC.

Synthesis of 11 (DA(2CPDTV)):

A deoxygenated mix of **9** (50 mg, 0.057 mmol), **10**[8] (70 mg, 0.14 mmol), $Pd(PPh_3)_4$ (9 mg, 0.007 mmol) and Na_2CO_3 (49 mg, 0.46 mmol) in 4 mL of water and 15 mL of anhydrous THF was stirred 16 h at 80ºC (monitored by TLC). After cooling to room temperature, the reaction mixture was extracted from diethyl ether and the organic phase was treated with an ammonium chloride aqueous solution and brine, and dried over $MgSO_4$. The solvent was evaporated under reduced pressure and the crude product was purified by chromatography column using silica gel and hexane:chloroform 3:2 mix as eluent. Pure product **11** was achieved as red solid in 66% of yield (60 mg, 0.037 mmol).

^{1}H-NMR (400 MHz, THF-d_8) δ/ppm: 7.46 (d, 4H, ^{3}J = 8.7 Hz), 7.25 (s, 2H), 7.07-7.04 (m, 12H), 6.92-6.86 (m, 12H), 3.97 (t, 8H, ^{3}J = 6.5 Hz), 1.94 (m, 8H), 1.81-1.79 (m, 8H), 1.52 (m, 8H), 1.40-1.39 (m, 16H), 1.20 (m, 24H), 1.05 (m, 8H), 0.96 (m, 12H), 0.86-0.83 (m, 12H); ^{13}C-NMR (100 MHz, THF-d_8) δ/ppm: 157.3, 155.8, 154.2, 146.1, 143.6, 141.6, 138.7, 133.8, 125.4, 124.5, 123.6, 118.6, 113.2, 65.8, 51.9, 36.0, 30.0, 29.6, 27.9, 27.5, 23.9, 20.7, 11.4; FT-IR (ATR) ν/cm^{-1}: 2954, 2924, 2854, 1504, 1238, 822, 725; UV-Vis (CH_2Cl_2) λ_{max}/nm (log ε): 515 (4.86); MS (*m/z*) (MALDI-TOF): calculated $C_{104}H_{134}N_2O_4S_4$: 1604.45; found: 1604.33 (M+).

Synthesis of Q(2CPDTV):

In a glove box, malononitrile (66 mg, 1.0 mmol) in dry THF (1 mL) was added to a suspension of sodium hydride (60% in oil, 80 mg, 2.0 mmol) in dry THF (4 mL) and the

[8] Margalias, A.; Seintis, K.; Yigit, M. Z.; Can, M.; Sygkridou, D.; Giannetas, V.; Fakis, M.; Stathatos, E. *Dyes Pigments* **2015**, *121*, 316-327.

mixture was stirred for 30 min at rt. To this mixture was added 9 (175 mg, 0.200 mmol), Pd(PPh3)4 (23 mg, 0.020 mmol), diphenylphosphinoferrocene (dppf, 22 mg, 0.040 mmol) and an additional 3 mL of dry THF. The apparatus was removed from the glovebox and the reaction was refluxed for 10 h while purging with N2. After cooling to 0 ºC, 2 mL of THF and 5 mL aqueous HCl (1.0 M) was added. After stirring for 2 h under air, the mixture was extracted with dichloromethane (3 x 10 mL) and the extracts were combined, dried with MgSO4 and concentrated. The crude material was purified with silica gel column chromatography using the following solvents: (i) 1:1 CH2Cl2:hexanes; (ii) 2:1 CH_2Cl_2:hexanes; and (iii) CH_2Cl_2. Pure fractions were combined and concentrated to afford 121 mg (72%) of **Q(2CPDTV)** as a dark metallic green solid.

1H NMR (300 MHz, CDCl3) δ 6.91 (s, 2H), 6.71 (s, 4H), 1.85 (m, 8H), 1.20 (m, 24H), 1.01 (m, 8H), 0.85 (t, 12H). HRMS-ESI (m/z): [M]+calcd for C44H58N4S4: 842.3544; found: 842.3529.

General Procedure for the synthesis of oligomers *n*CPDTV (Horner-Wadsworth-Emmons reaction):

Under Ar atmosphere, ^{t}BuOK was added to a solution of the corresponding *bis*-aldehyde and CPDT-phosponate (**2**) in anhydrous THF, in the corresponding stoichiometric ratio. The reaction was stirred at room temperature. After consumption of the corresponding *bis*-aldehyde (monitored by TLC), the reaction mixture was quenched with water. The organic phase was extracted with diethylether and dried over $MgSO_4$. The solvent was evaporated *in vacuo* and the obtained solid was purified by chromatography column (silica gel).

2CPDTV: ^{t}BuOK (90.4 mg, 0.80 mmol) **1** (157.3 mg, 0.40 mmol), **2** (200 mg, 0.40 mmol) and 25 mL of anhydrous THF. Reaction time: overnight. Eluent: hexane. Yield: 86% (250 mg, 0.35 mmol).

^{1}H-NMR (400 MHz, $CDCl_3$) δ/ppm: 7.17 (d, 2H, ^{3}J = 4.7 Hz), 7.00 (s, 2H), 6.93 (d, 2H, ^{3}J = 4.7 Hz), 6.87 (s, 2H), 1.82 (t, 8H, ^{3}J = 8.1 Hz), 1.21-1.15 (m, 24H), 0.97-0.96 (m, 8H), 0.84-0.81 (m, 12H); ^{13}C-NMR (100 MHz, $CDCl_3$) δ/ppm: 158.5, 158.3, 143.1, 136.7, 135.4, 125.0, 121.6, 120.4, 120.3, 53.5, 37.8, 31.6, 29.7, 24.5, 22.6, 14.1; FT-IR (KBr) υ/cm^{-1}: 2953, 2926, 2854, 1466, 1408, 1375, 1136, 937, 708, 660; UV-Vis

(CH_2Cl_2) λ_{max}/nm (log ε): 450 (4.72), 474 (4.66); MS (*m/z*) (MALDI-TOF): calculated $C_{44}H_{60}S_4$: 717.21; found: 716.96 (M^+). Melting point: 162 ºC.

3CPDTV: ^tBuOK (169 mg, 1.51 mmol), **3** (75.8 mg, 0.19 mmol) and **2** (187 mg, 0.38 mmol) in 30 mL of anhydrous THF. Reaction time: 6 h. Eluent: Hexane. Yield: 48% (99 mg, 0.091 mmol).

^1H-NMR (400 MHz, $CDCl_3$) δ/ppm: 7.18 (d, 2H, ^3J = 4.7 Hz), 7.00 (s, 4H), 6.93 (d, 2H, ^3J = 4.7 Hz), 6.88 (s, 2H), 6.86 (s, 2H), 1.82-1.80 (m, 12H), 1.26-1.15 (m, 36H), 0.98 (m, 12H), 0.84-0.81(m, 18H); ^{13}C-NMR (100 MHz, $CDCl_3$) δ/ppm: 158.8, 158.6, 158.3, 143.7, 143.1, 136.7, 135.7, 135.6, 125.1, 121.6, 120.5, 120.4, 120.3, 120.2, 53.7, 53.5, 37.8, 31.6, 29.7, 24.5, 22.6, 14.1; FT-IR (KBr) υ/cm^{-1}: 2953, 2926, 2854, 1466, 1410, 1377, 926, 656; UV-Vis (CH_2Cl_2) λ_{max}/nm (log ε): 519 (5.00), 553 (4.96); MS (*m/z*) (MALDI-TOF): calculated $C_{67}H_{90}S_6$: 1087.82; found: 1086.89 (M^+). Melting point: 119 ºC.

4CPDTV: ^tBuOK (289.2 mg, 2.58 mmol), **4** (249 mg, 0.32 mmol) and **2** (320 mg, 0.64 mmol) in 45 mL of anhydrous THF. Reaction time: overnight. Eluent: hexane:$CHCl_3$ (1:1). Yield: 75% (353 mg, 0.24 mmol).

^1H-NMR (400 MHz, $CDCl_3$) δ/ppm: 7.18 (d, 2H, ^3J = 4.7 Hz), 7.01 (s, 6H), 6.94 (d, 2H ^3J = 4.7 Hz), 6.88 (s, 2H), 6.86 (s, 4H), 1.84-1.81 (m, 16H), 1.26-1.16 (m, 48H), 0.98 (m, 16H), 0.85-0.81 (m, 24H); ^{13}C-NMR (100 MHz, $CDCl_3$) δ/ppm: 158.9, 158.6, 158.4, 143.7, 143.1, 136.7, 135.7, 125.1, 121.6, 120.3, 53.7, 53.5, 37.9, 37.8, 31.7, 31.6, 29.7, 24.5, 22.7, 22.6, 14.1; FT-IR (KBr) υ/cm^{-1}: 2953, 2926, 2854, 1460, 1410, 1377, 926, 829, 656; UV-Vis (CH_2Cl_2) λ_{max}/nm (log ε): 559 (5.17), 597 (5.11); MS (*m/z*) (MALDI-TOF): calculated $C_{90}H_{120}S_8$: 1458.44; found: 1457.25 (M^+). Melting point: 135 ºC.

5CPDTV: ^tBuOK (204 mg, 1.82 mmol), **5** (260 mg, 0.23 mmol) and **2** (226 mg, 0.45 mmol) in 38 mL of anhydrous THF. Reaction time: 6h. Eluent: hexane:$CHCl_3$ (1:1). Yield: 69% (290 mg, 0.16 mmol).

^1H-NMR (400 MHz, $CDCl_3$) δ/ppm: 7.18 (s, 2H), 7.00 (s, 8H), 6.93 (s, 2H), 6.86 (s, 8H), 1.82 (s, 20H), 1.26-1.16 (m, 60H), 0.98 (s, 20H), 0.83 (s, 30H); ^{13}C-NMR (100 MHz, $CDCl_3$) δ/ppm: 158.9, 158.6, 158.4, 143.7, 143.1, 136.7, 135.8, 125.1, 121.6, 120.3, 53.7, 53.5, 37.9, 37.8, 31.7, 31.6, 29.8, 29.7, 24.5, 22.7, 22.6, 14.1; FT-IR (KBr) ν/cm^{-1}: 2953, 2926, 2852, 1460, 1412, 924, 654, 561; UV-Vis (CH_2Cl_2) λ_{max}/nm (log ε): 581 (5.28),

620 (5.24); MS (*m/z*) (MALDI-TOF): calculated $C_{113}H_{150}S_{10}$: 1826.89; found: 1826.90 (M^{+}). Melting point: 173 ºC.

<u>6CPDTV</u>: ^{t}BuOK (527.6 mg, 4.70 mmol), **6** (356 mg, 0.24 mmol) and **2** (233.5 mg, 0.47 mmol) in 60 mL of anhydrous THF. Reaction time: overnight. Eluent: hexane:$CHCl_3$ (1:1). Yield: 75% (390 mg, 0.18 mmol).

^{1}H-NMR (400 MHz, $CDCl_3$) δ/ppm: 7.18 (d, 2H, ^{3}J = 4.7 Hz), 7.01 (s, 10H), 6.93 (d, 2H, ^{3}J = 4.7 Hz), 6.88-6.87 (m, 10H), 1.82 (m, 24H), 1.26-1.16 (m, 72H), 0.98 (m, 24H), 0.84 (m, 36H); ^{13}C-NMR (100 MHz, $CDCl_3$) δ/ppm: 158.9, 158.8, 158.6, 158.4, 143.8, 143.1, 136.7, 135.8, 120.4, 120.3, 53.7, 53.5, 53.4, 37.9, 31.7, 31.0, 29.7, 24.5, 22.7, 14.1; FT-IR (KBr) υ/cm^{-1}: 2953, 2926, 2852, 1458, 1410, 1377, 1176, 1128, 924; UV-Vis (CH_2Cl_2) λ_{max}/nm (log ε): 596 (5.30), 636 (5.26); MS (*m/z*) (MALDI-TOF): calculated $C_{136}H_{180}S_{12}$: 2199.66; found: 2198.65 (M^{+}). Melting point: 210 ºC.

3. ^{1}H NMR, ^{13}C NMR, MS and IR spectra

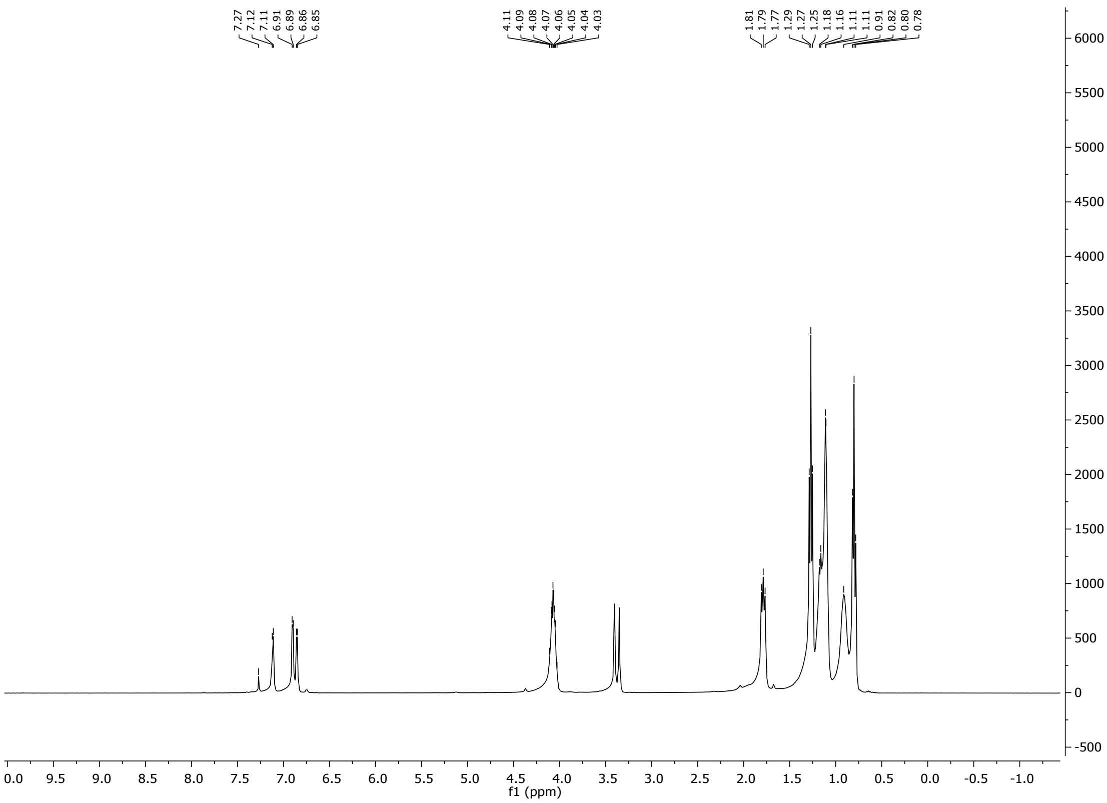

Figure S 1. 1H NMR spectrum (400 MHz, $CDCl_3$) of **2**.

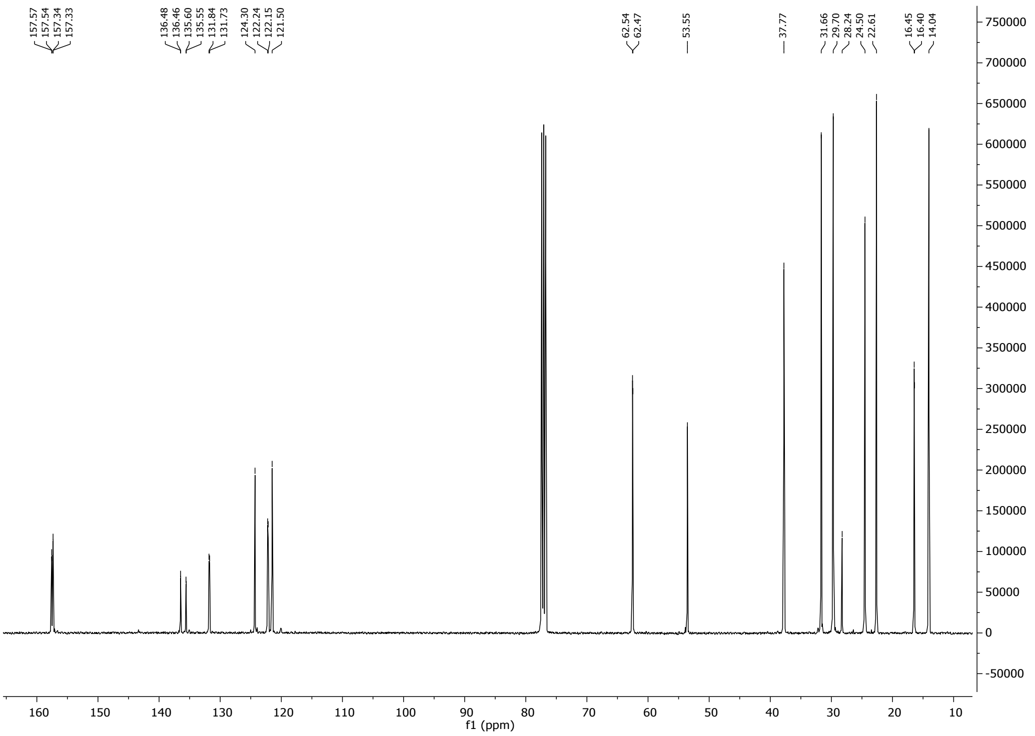

Figure S 2. ^{13}C NMR spectrum (100 MHz, $CDCl_3$) of **2.**

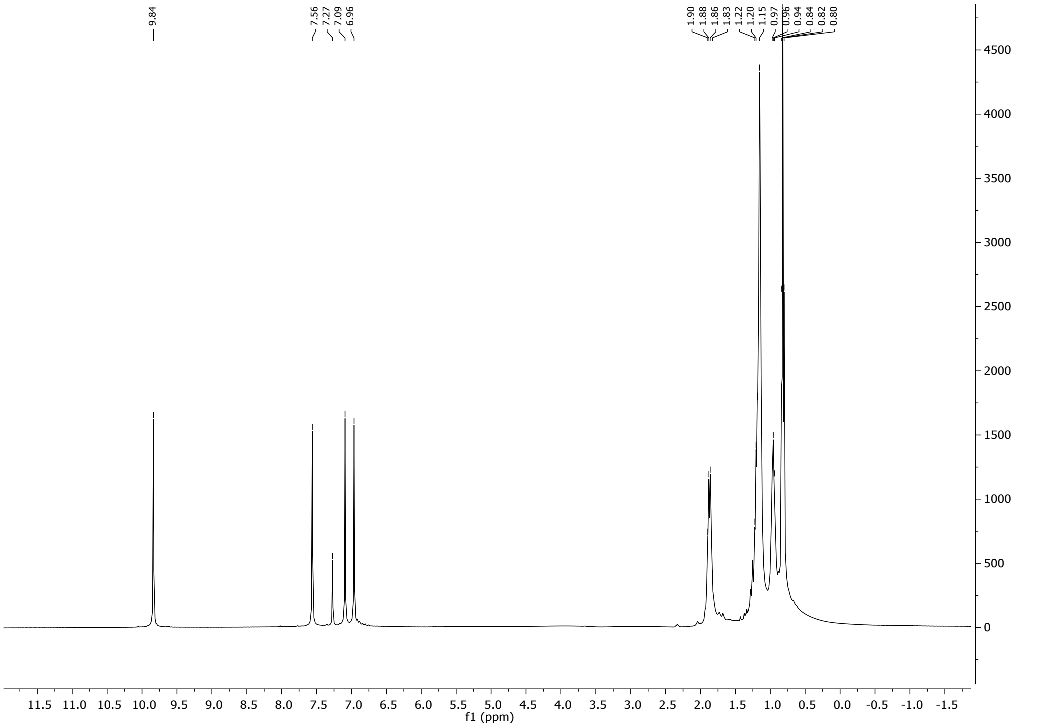

Figure S 3. ^{1}H NMR spectrum (400 MHz, $CDCl_3$) of **4**.

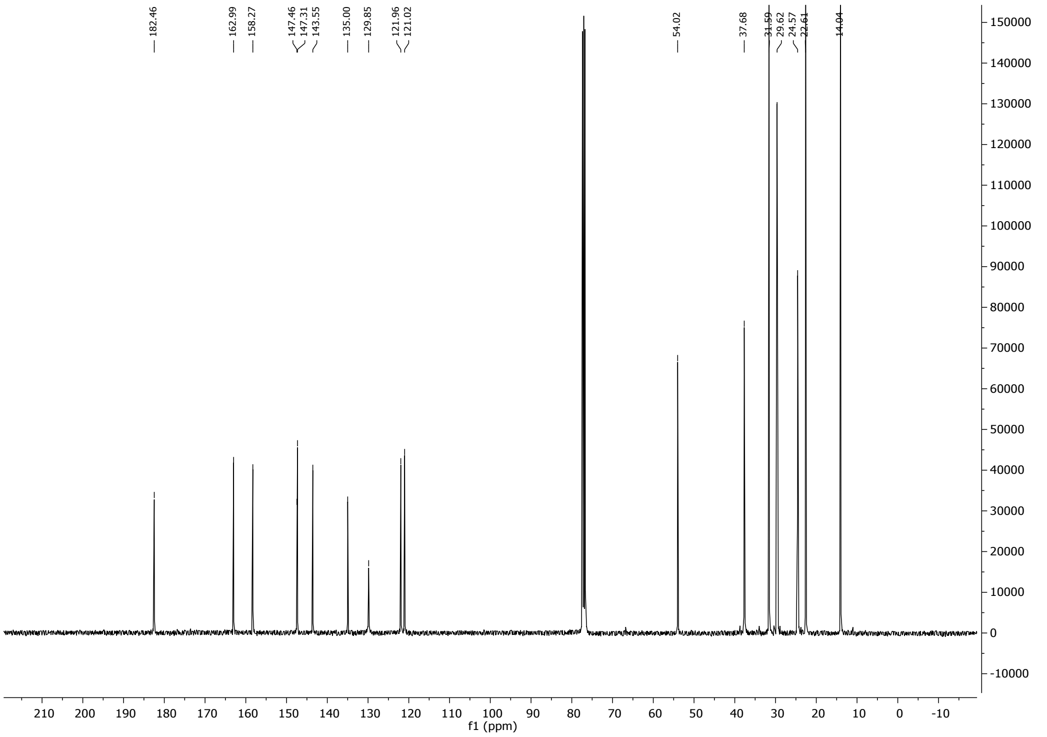

Figure S 4. ^{13}C NMR spectrum (100 MHz, $CDCl_3$) of **4.**

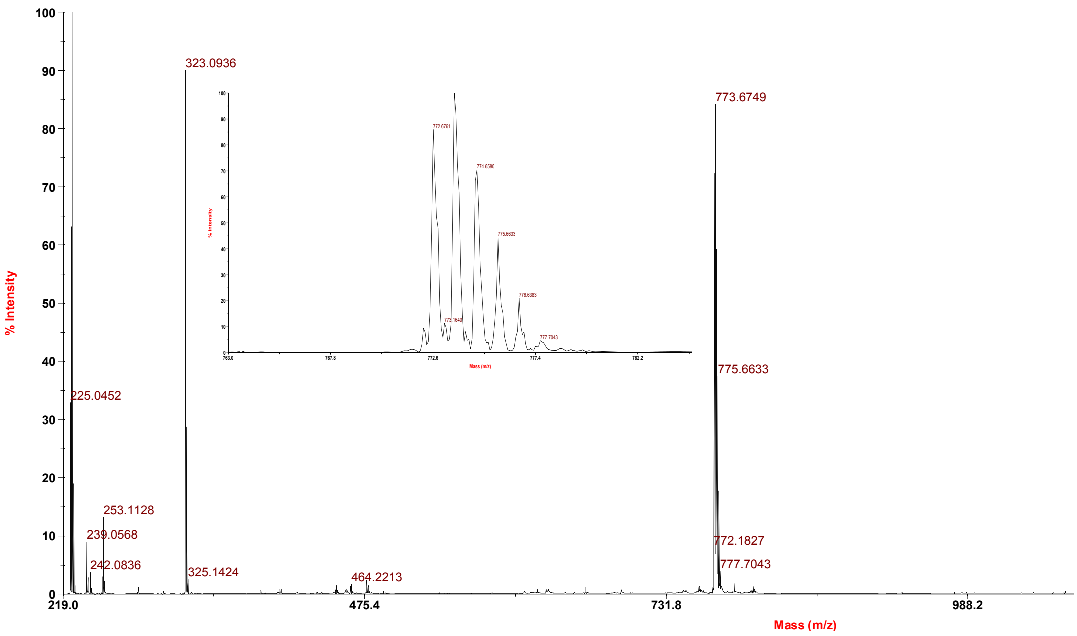

Figure S 5. MALDI-MS spectrum of **4** (Matrix: Dithranol).

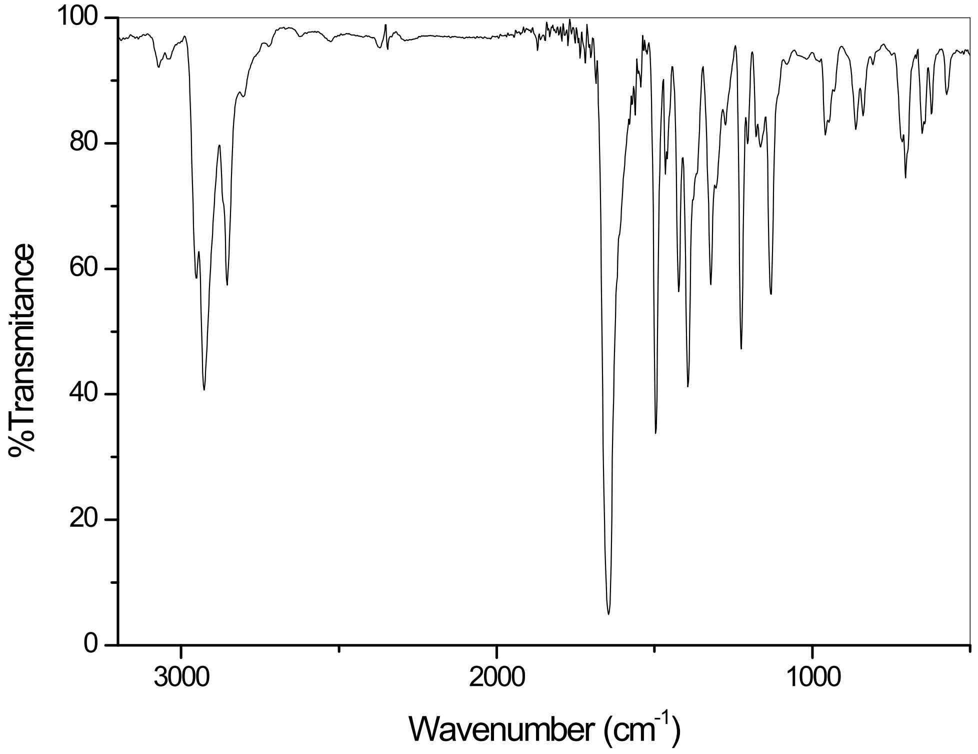

Figure S 6 FT-IR spectrum of **4**.

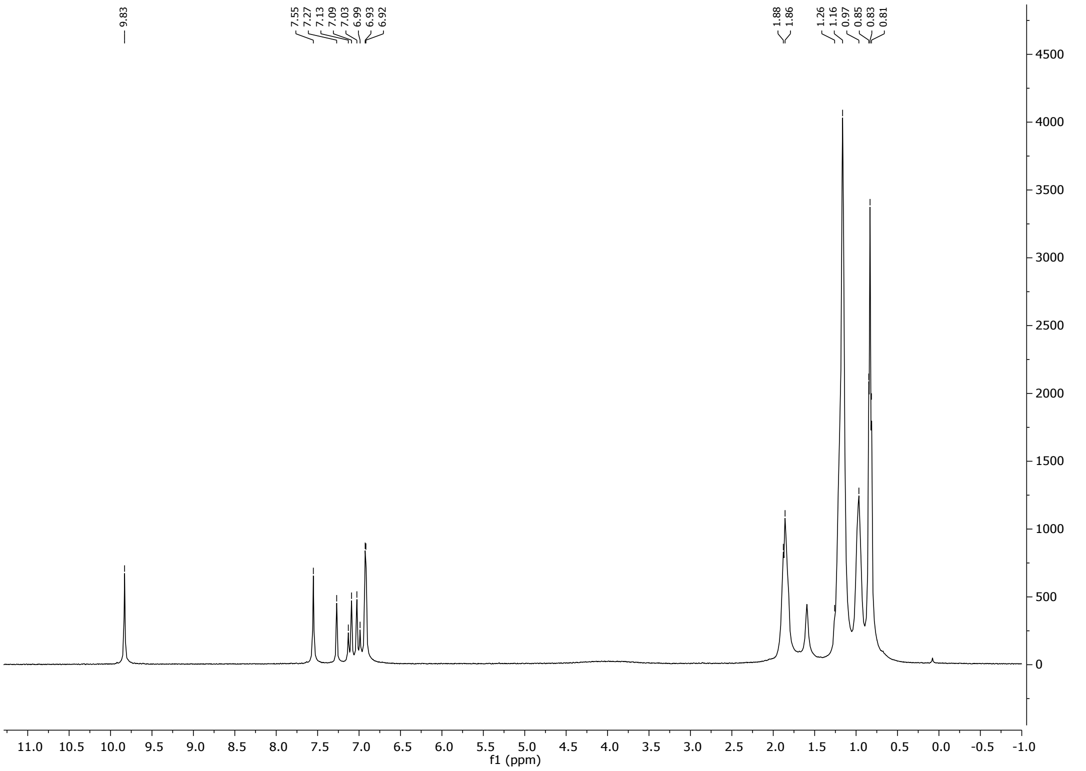

Figure S 7. ^{1}H NMR spectrum (400 MHz, $CDCl_3$) of **5**.

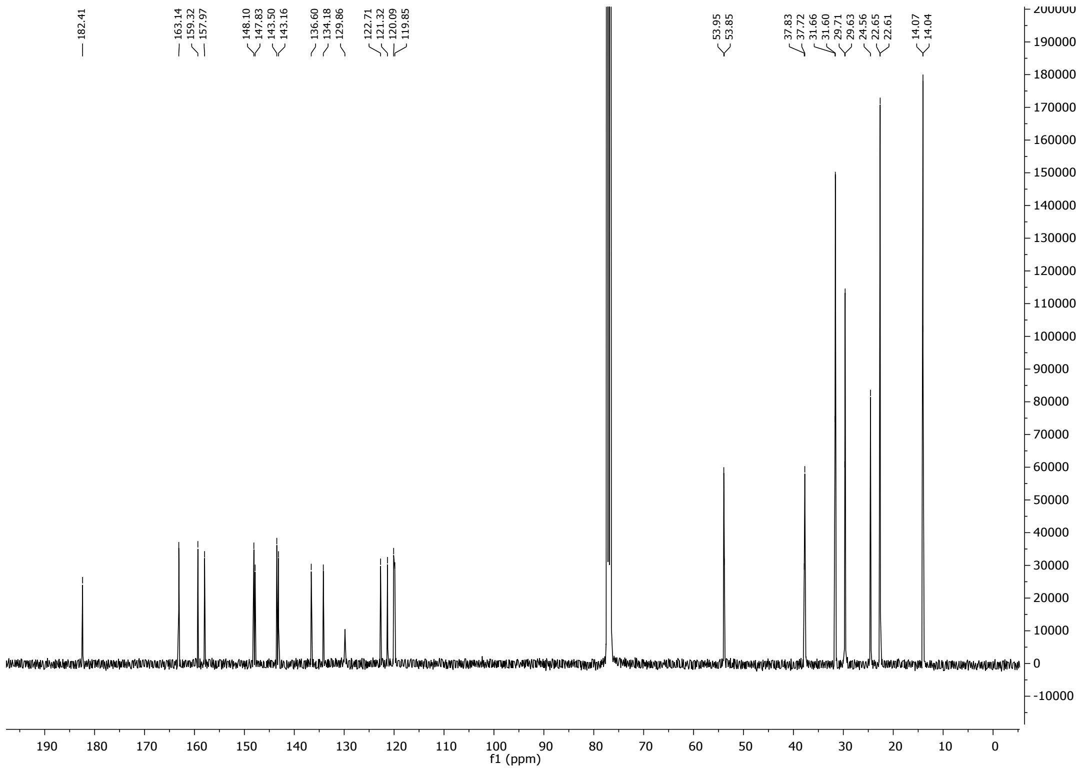

Figure S 8. ^{13}C NMR spectrum (100 MHz, $CDCl_3$) of **5.**

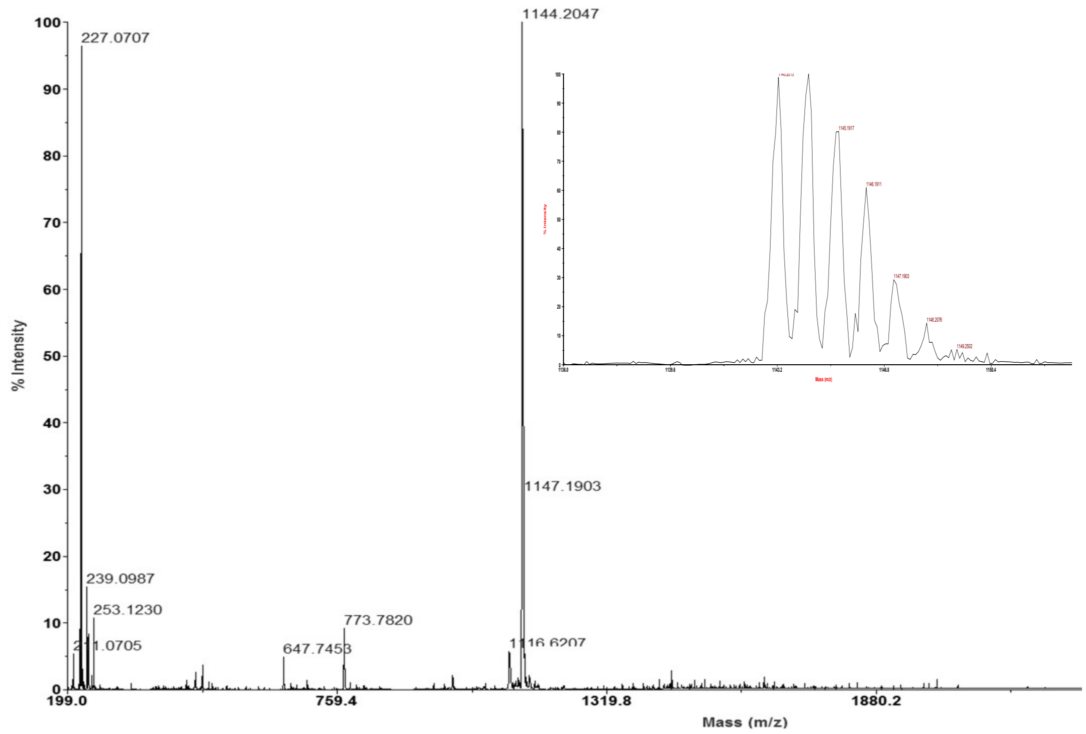

Figure S 9. MALDI-MS spectrum of **5** (Matrix: Dithranol).

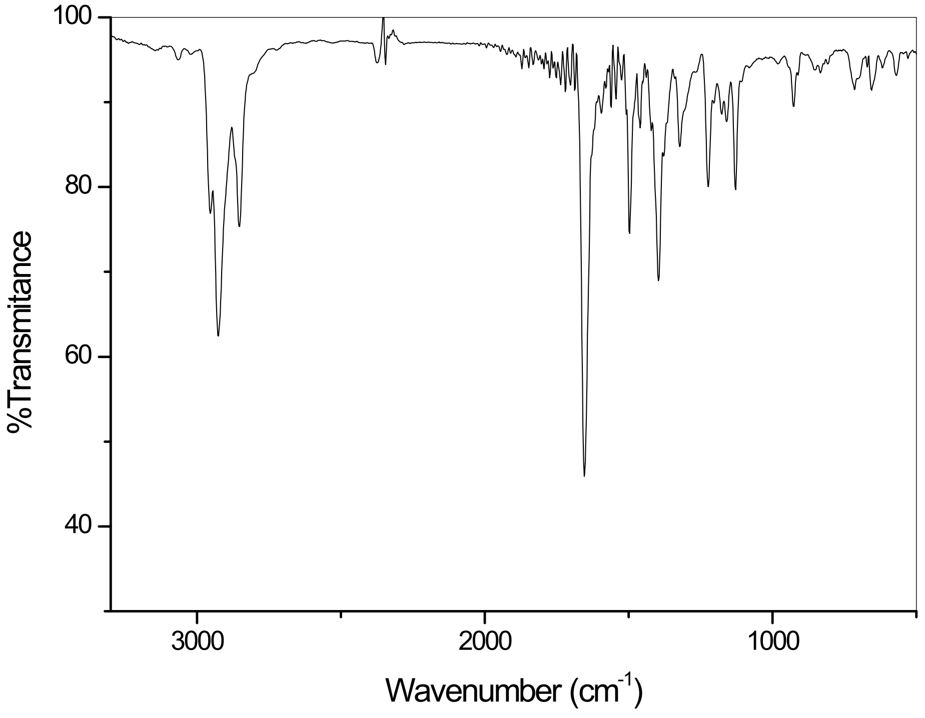

Figure S 10. FT-IR spectrum of **5**.

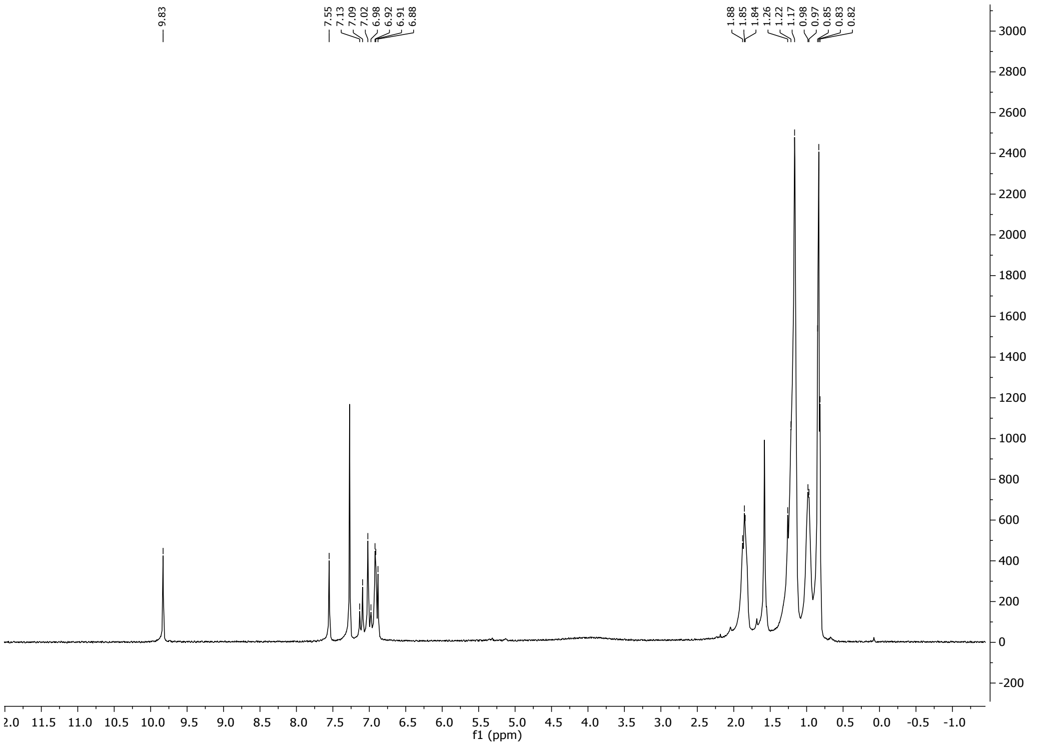

Figure S 11. ^{1}H NMR spectrum (400 MHz, $CDCl_3$) of **6**.

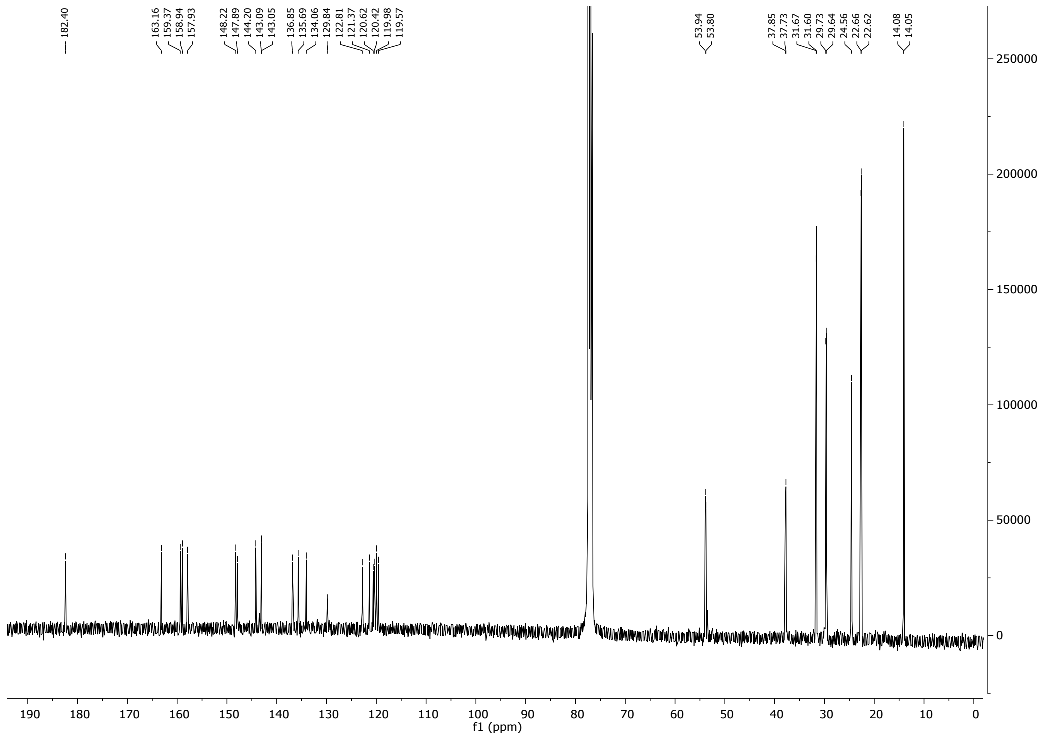

Figure S 12. ^{13}C NMR spectrum (100 MHz, $CDCl_3$) of **6.**

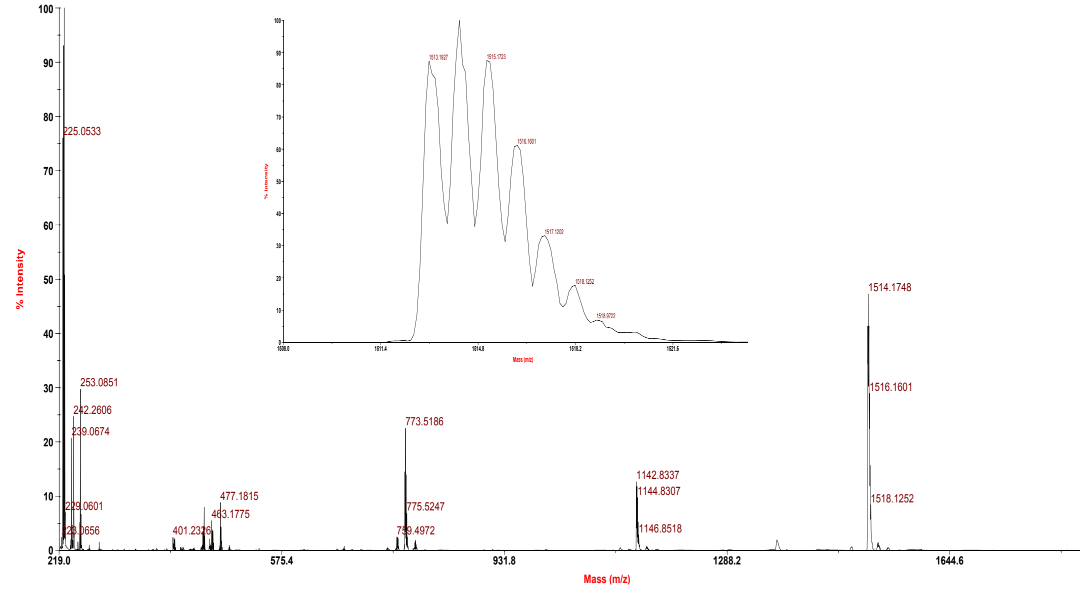

Figure S 13. MALDI-MS spectrum of **6** (Matrix: Dithranol).

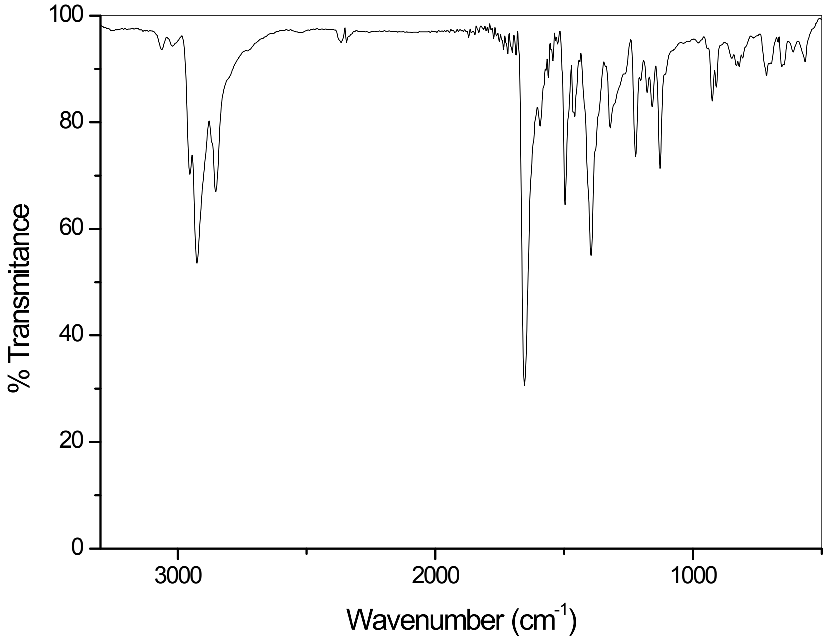

Figure S 14. FT-IR spectrum of **6**.

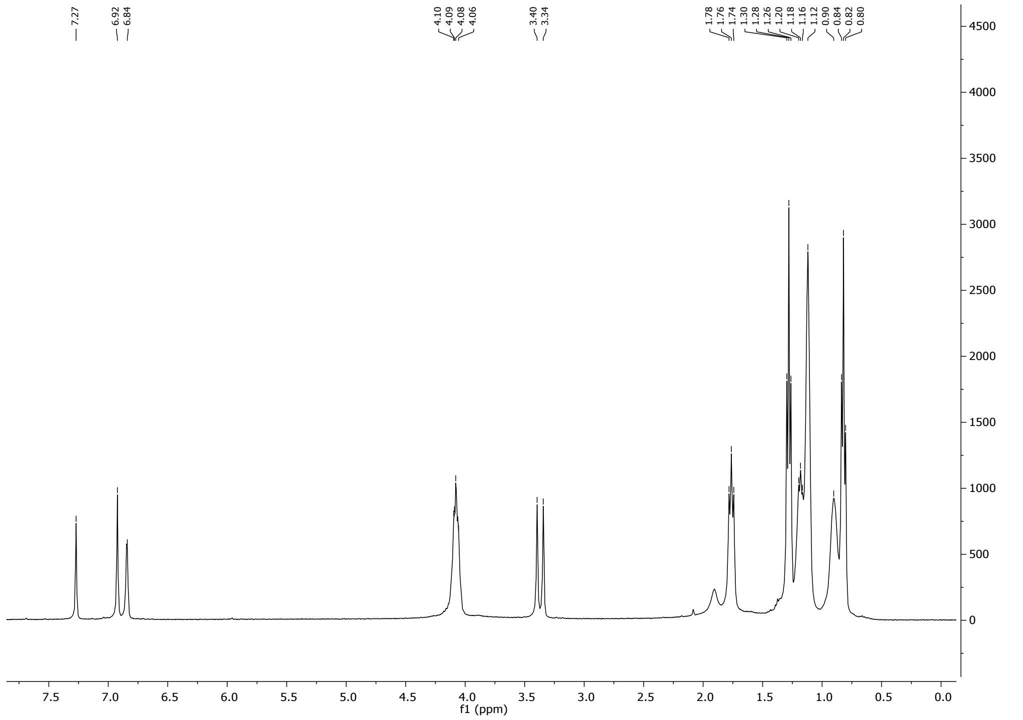

Figure S 15. ^{1}H NMR spectrum (400 MHz, $CDCl_3$) of **8**.

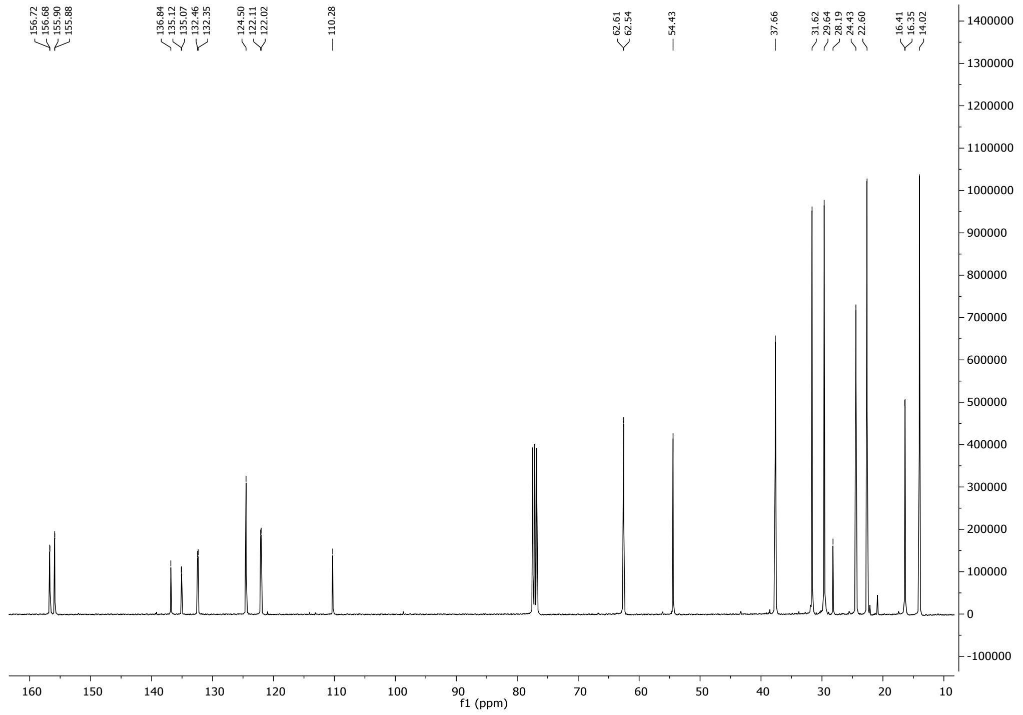

Figure S 16. ^{13}C NMR spectrum (100 MHz, $CDCl_3$) of **8.**

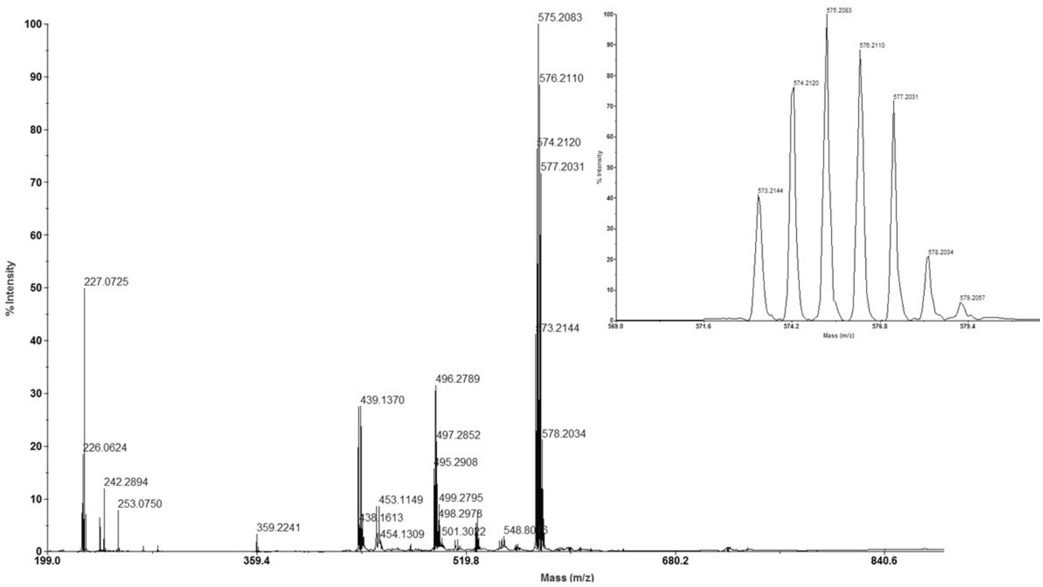

Figure S 17. MALDI-MS spectrum of **8** (Matrix: Dithranol).

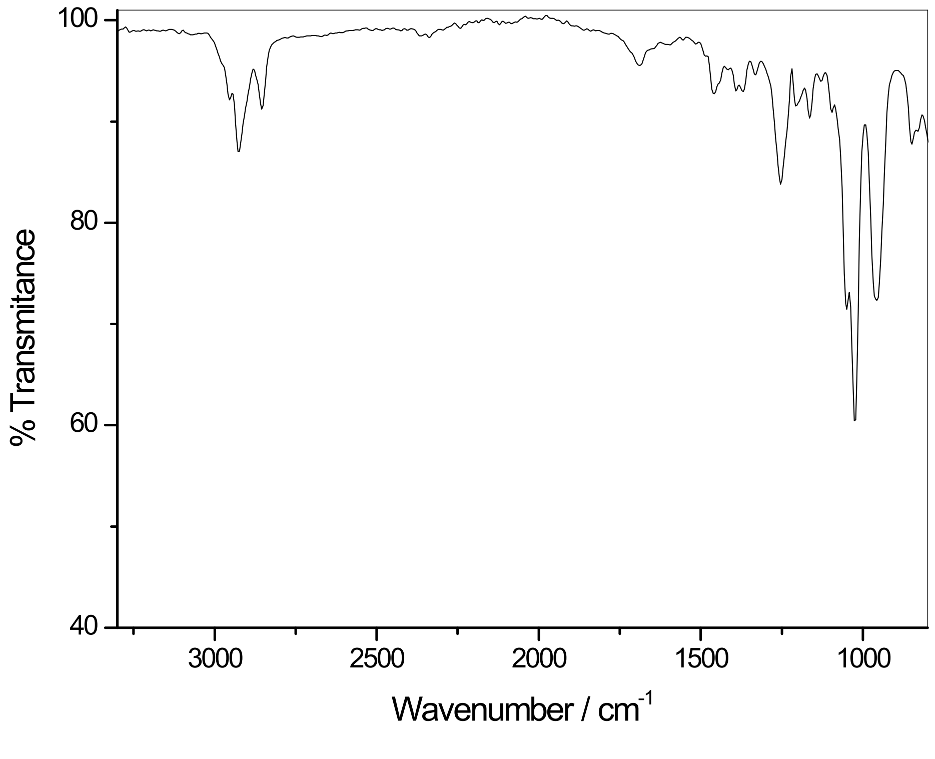

Figure S 18. FT-IR spectrum of **8**.

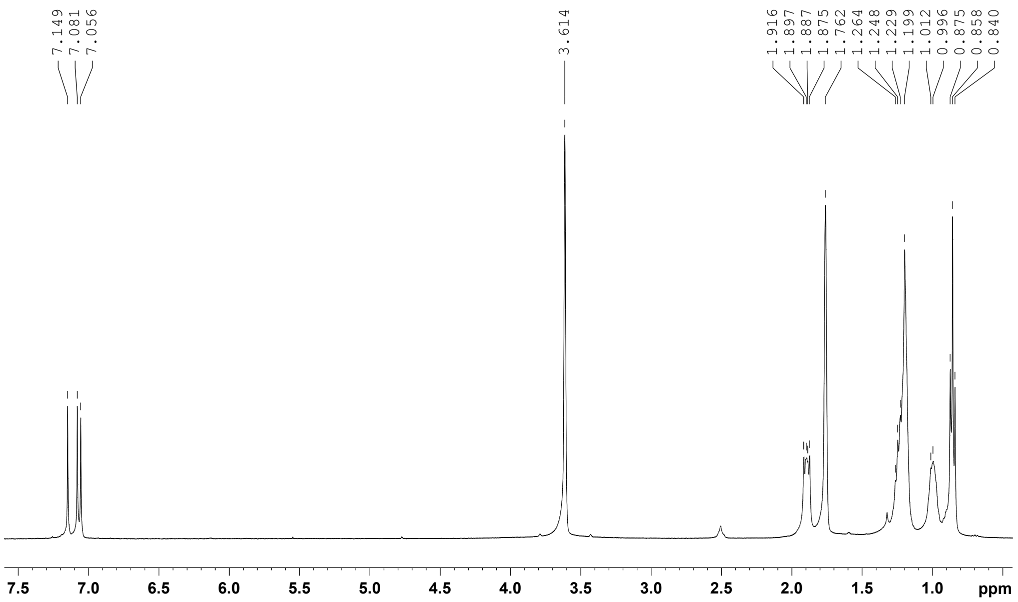

Figure S 19. ^{1}H NMR spectrum (400 MHz, THF-d_8) of derivative **9**.

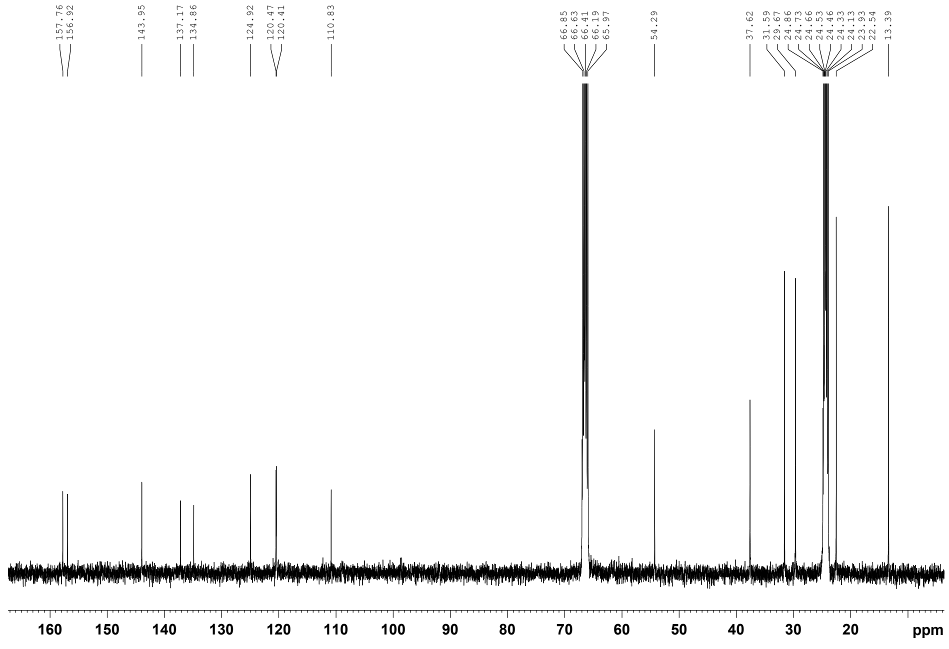

Figure S 20. ^{13}C NMR spectrum (100 MHz, THF-d_8) of derivative **9**.

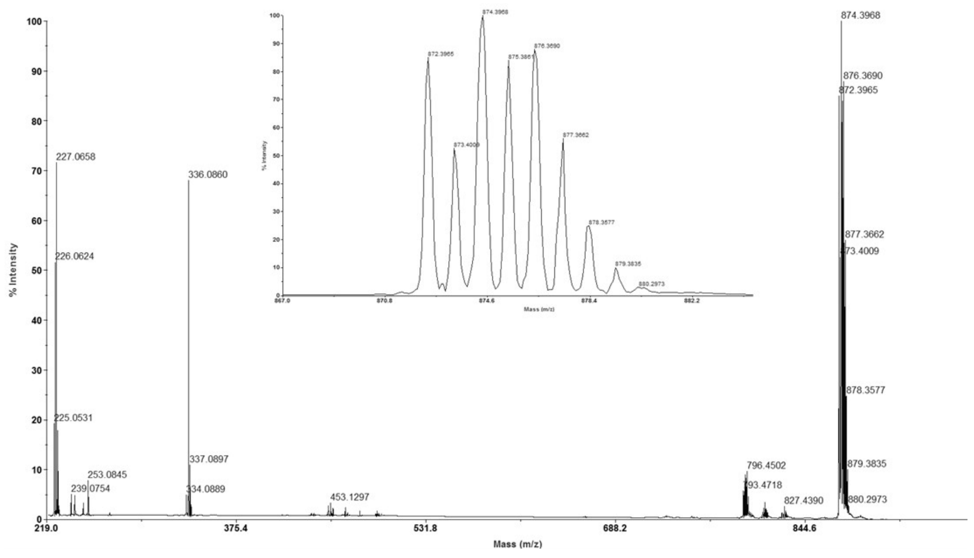

Figure S 21. MALDI-MS spectrum of derivative **9** (Matrix: Dithranol).

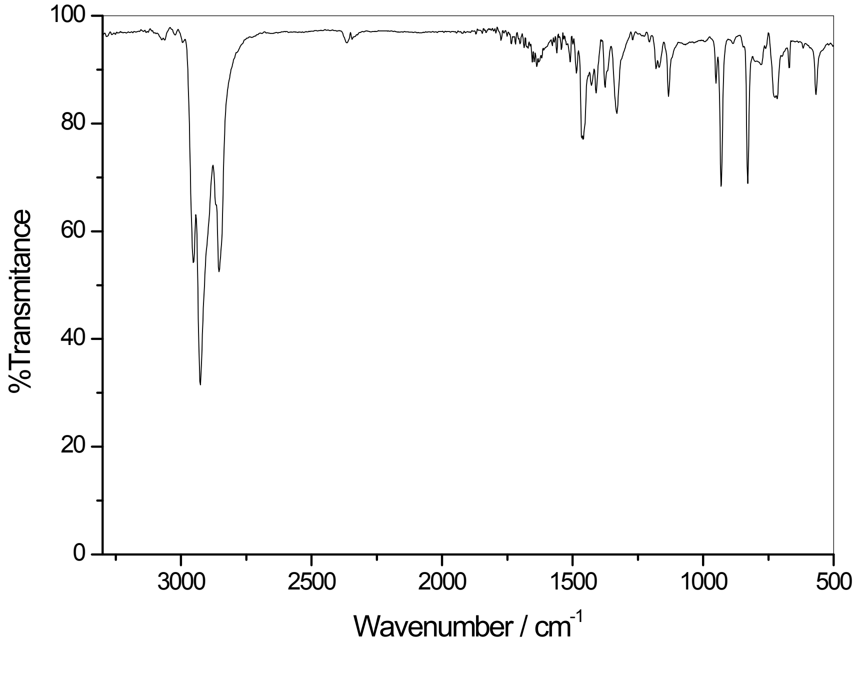

Figure S 22. FT-IR spectrum of derivative **9**.

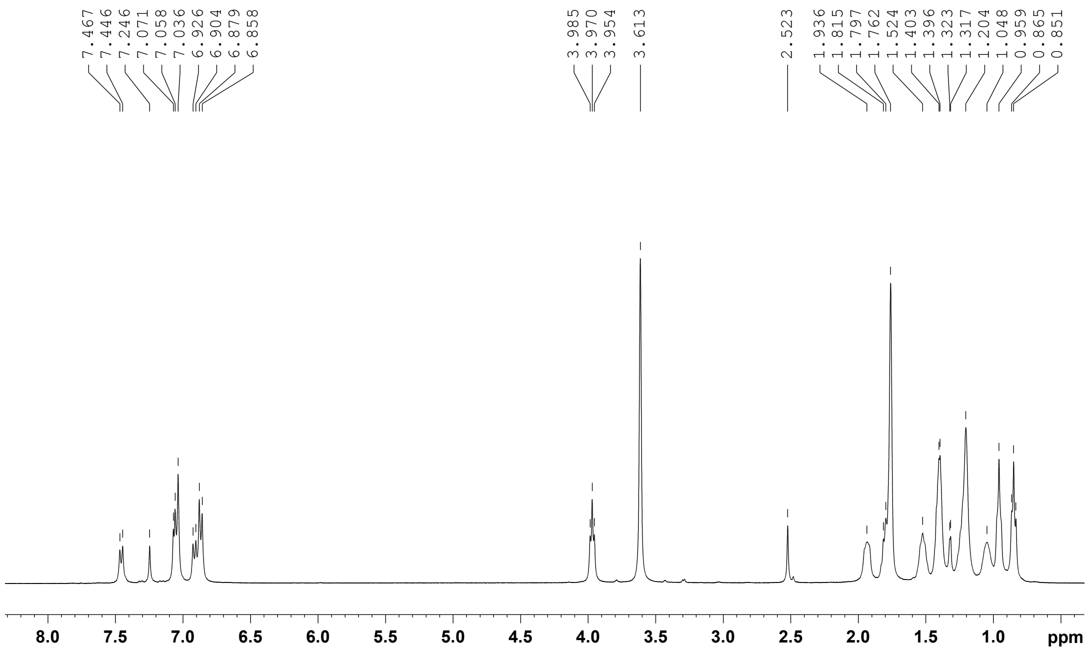

Figure S 23. ^{1}H NMR spectrum (400 MHz, THF-d_8) of derivative **11**.

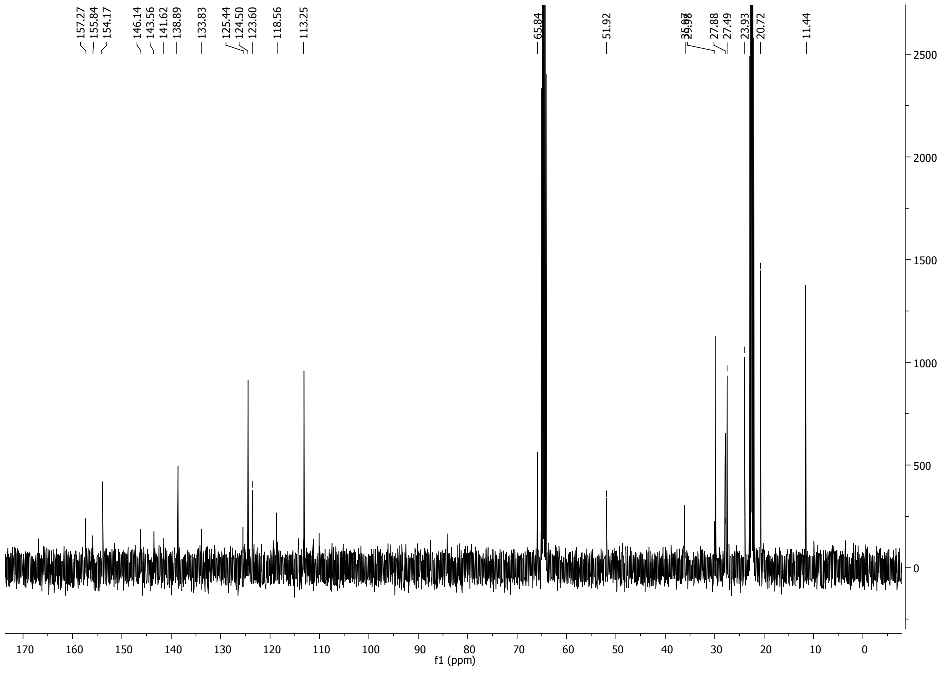

Figure S 24. ^{13}C NMR spectrum (100 MHz, THF-d_8) of derivative **11**.

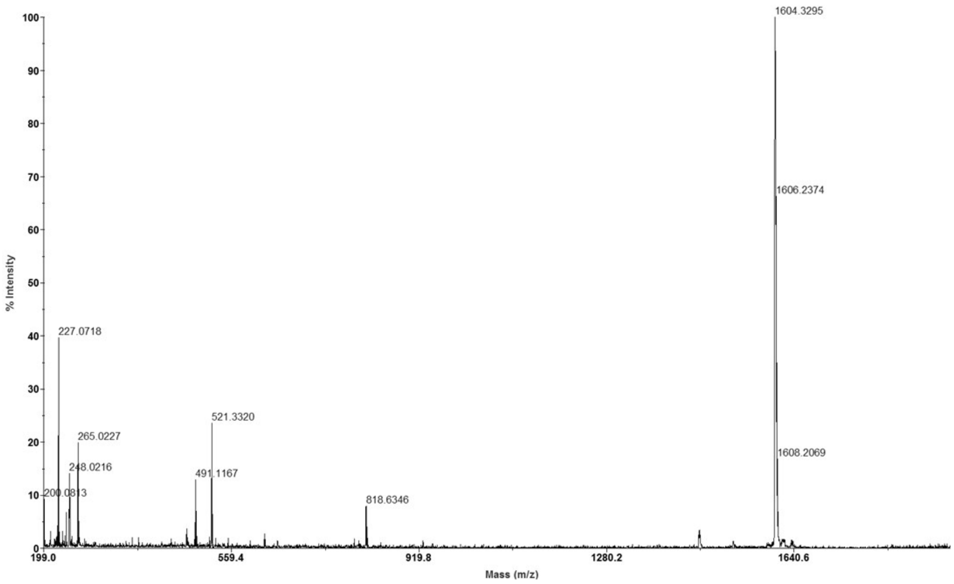

Figure S 25. MALDI-MS spectrum of derivative **11** (Matrix: Dithranol).

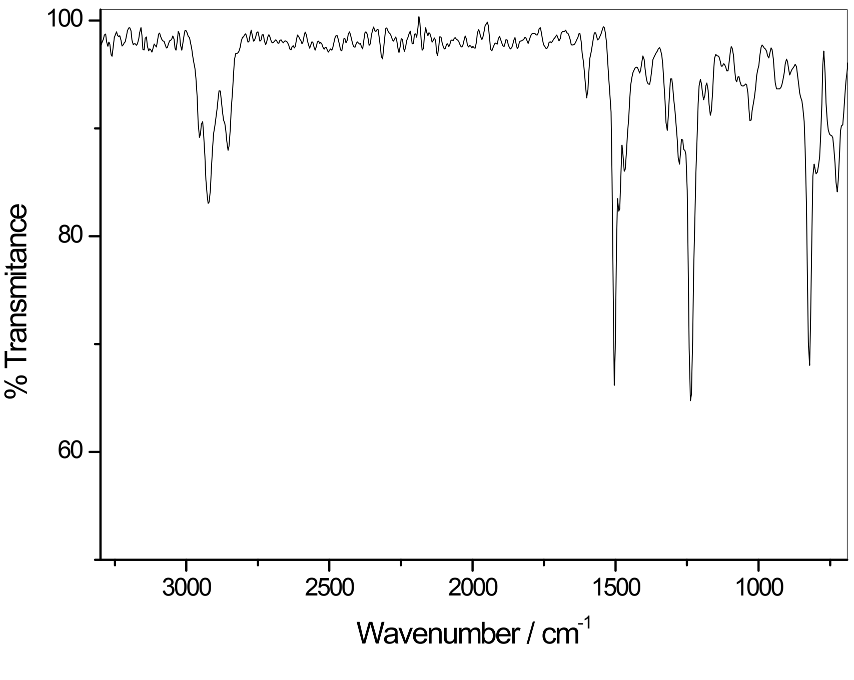

Figure S 26. FT-IR spectrum of derivative **11**.

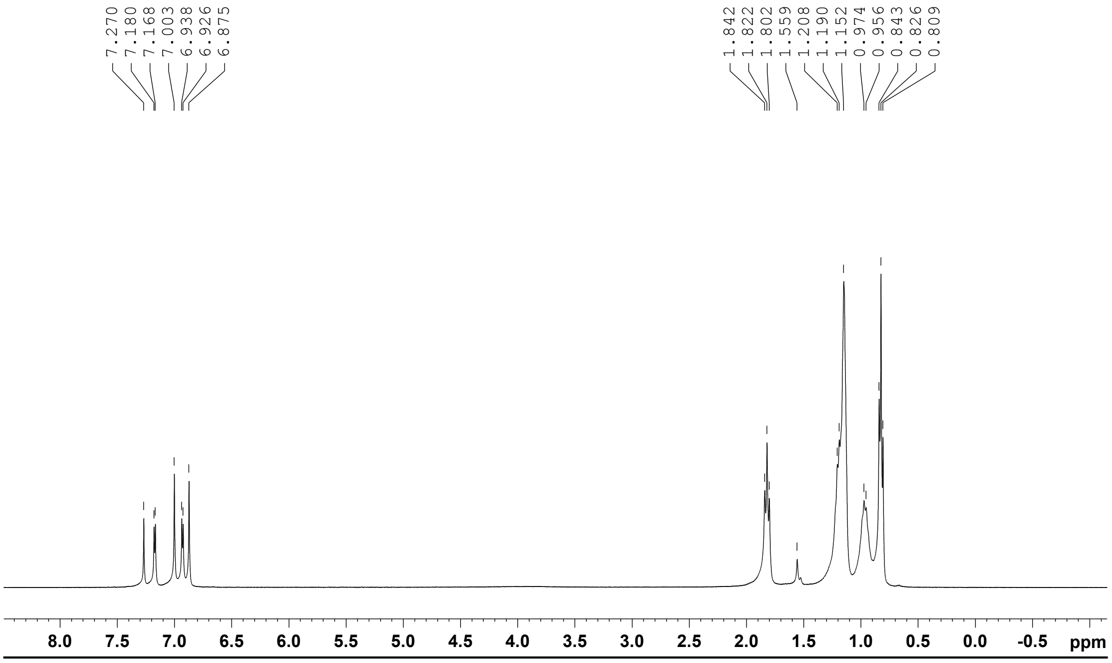

Figure S 27. ^{1}H NMR spectrum (400 MHz, $CDCl_3$) of **2CPDTV**.

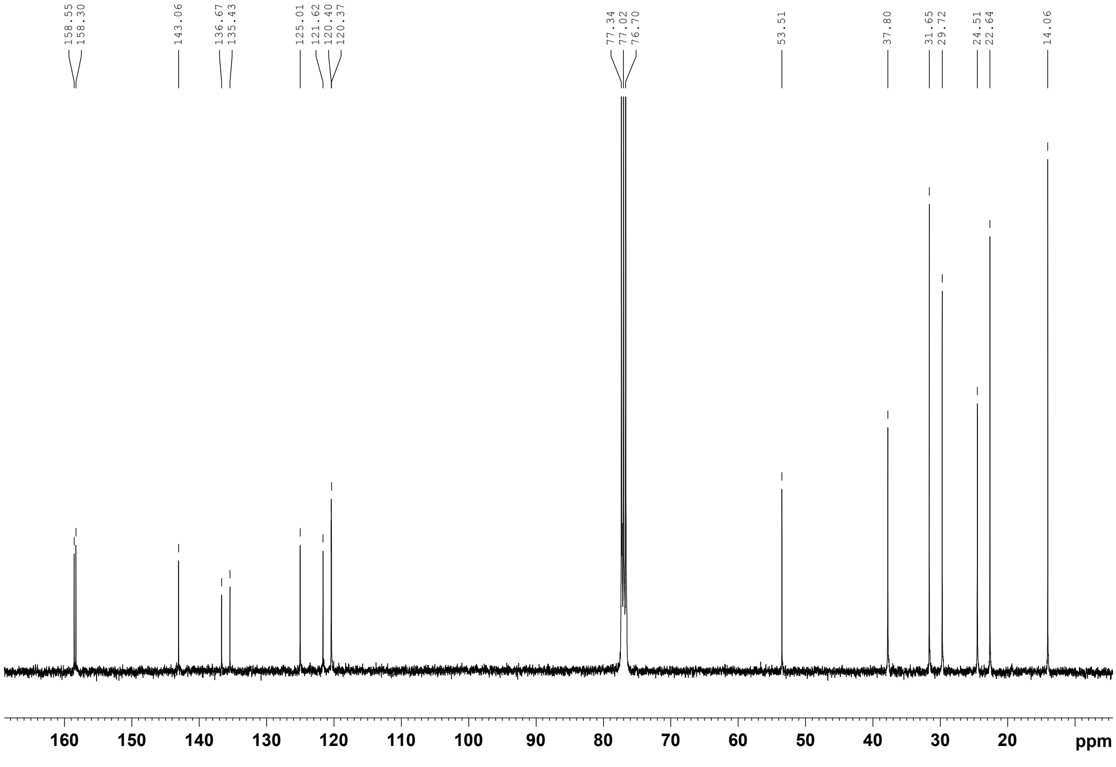

Figure S 28. ^{13}C NMR spectrum (100 MHz, $CDCl_3$) of **2CPDTV.**

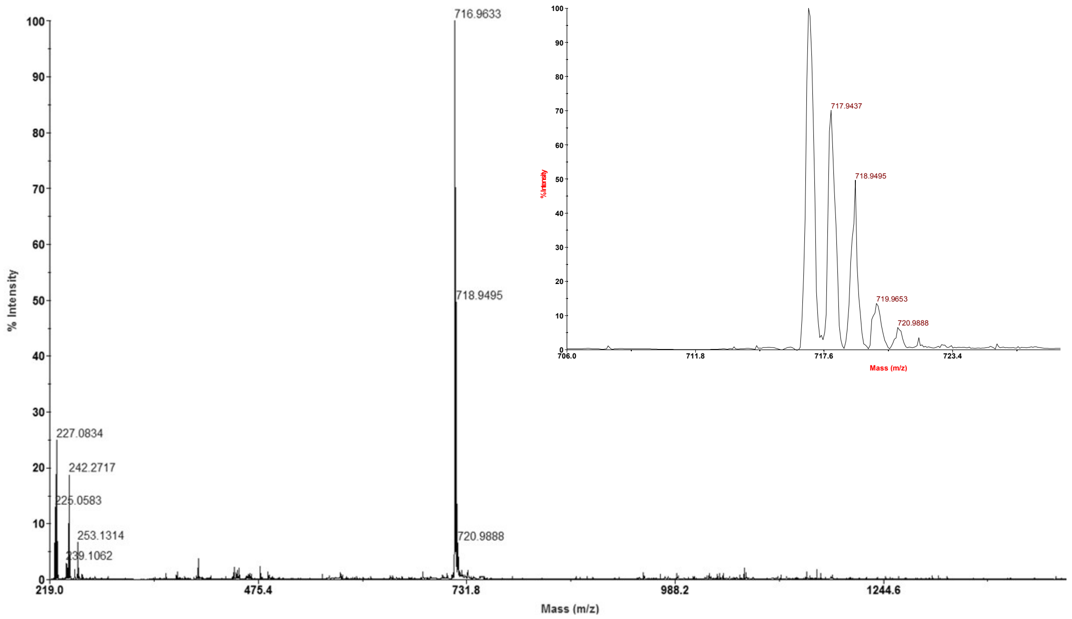

Figure S 29. MALDI-MS spectrum of **2CPDTV** (Matrix: Dithranol).

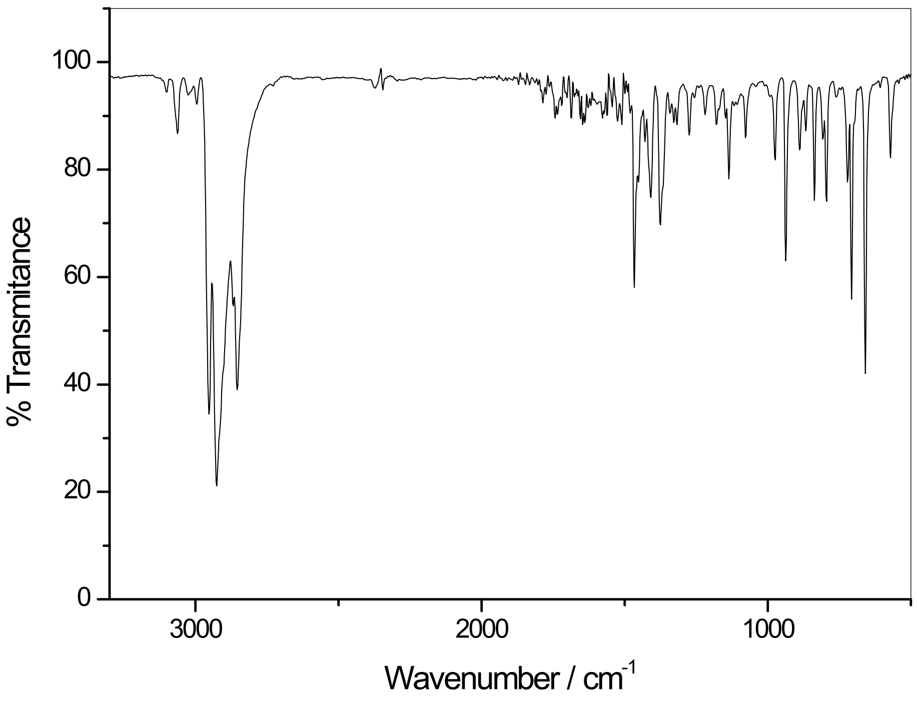

Figure S 30. FT-IR spectrum of **2CPDTV**.

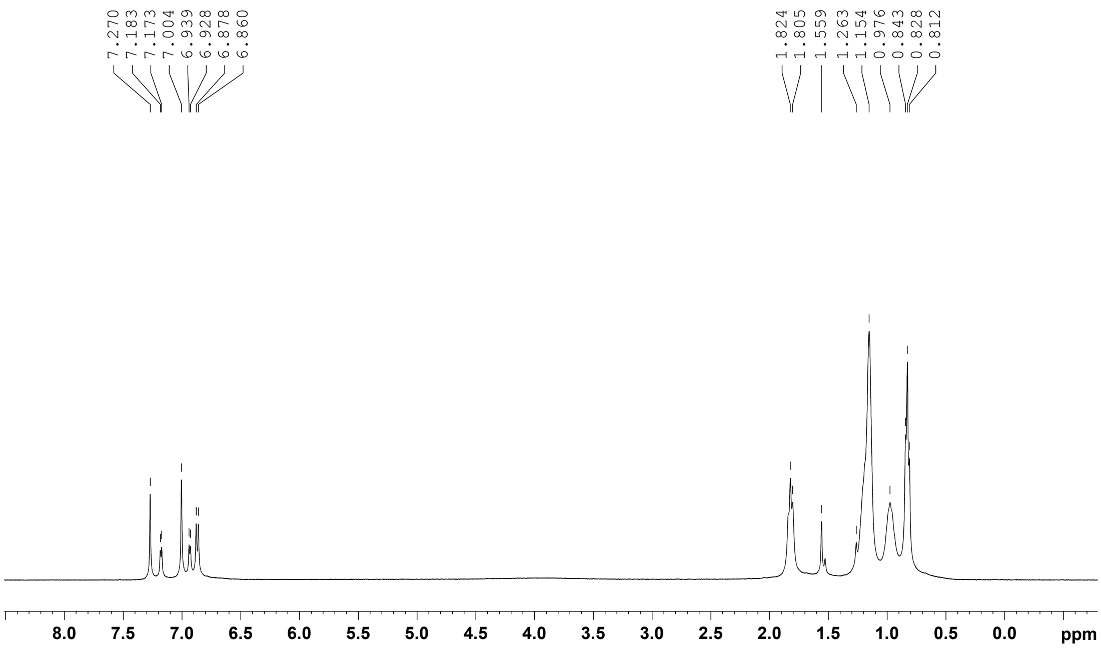

Figure S 31. ^{1}H NMR spectrum (400 MHz, $CDCl_3$) of **3CPDTV**.

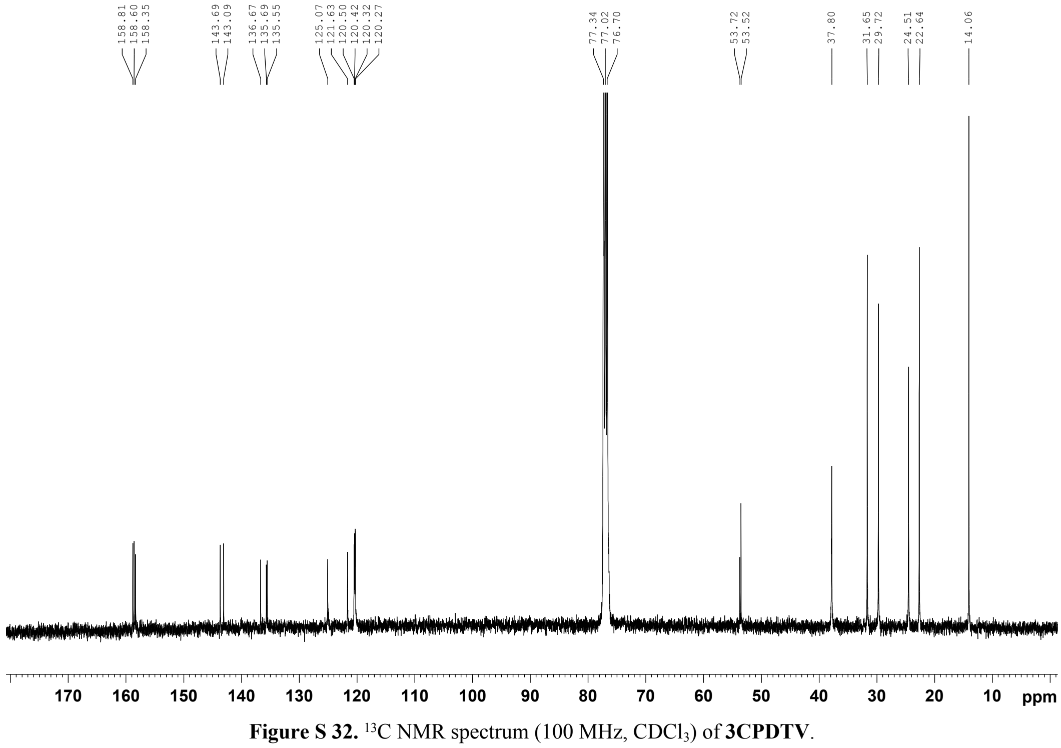

Figure S 32. ^{13}C NMR spectrum (100 MHz, $CDCl_3$) of **3CPDTV**.

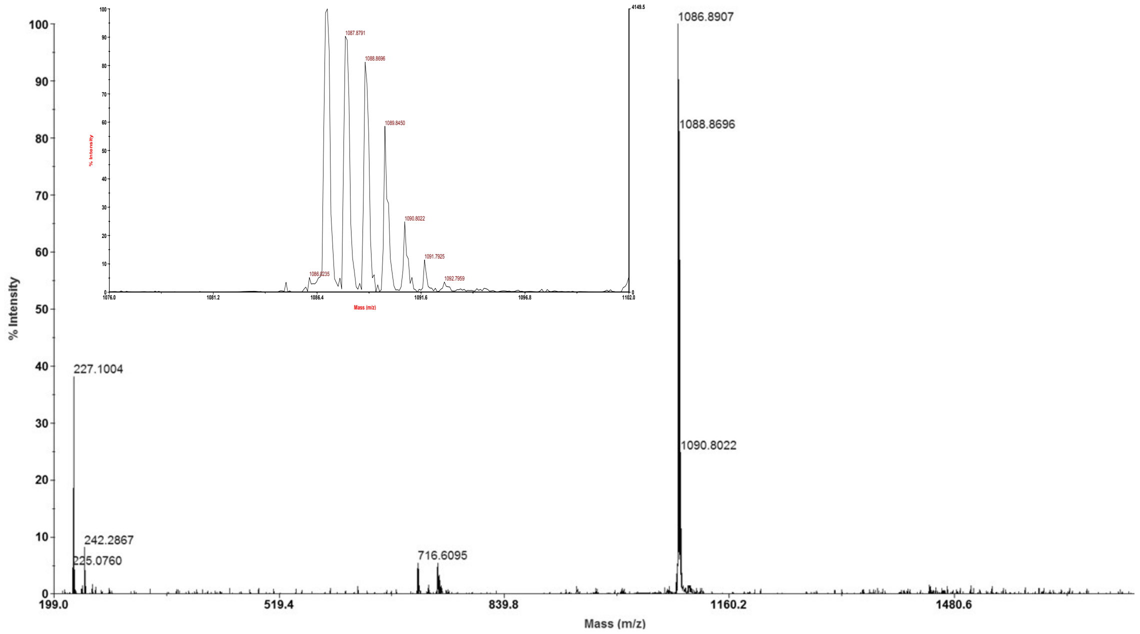

Figure S 33. MALDI-MS spectrum of **3CPDTV** (Matrix: Dithranol).

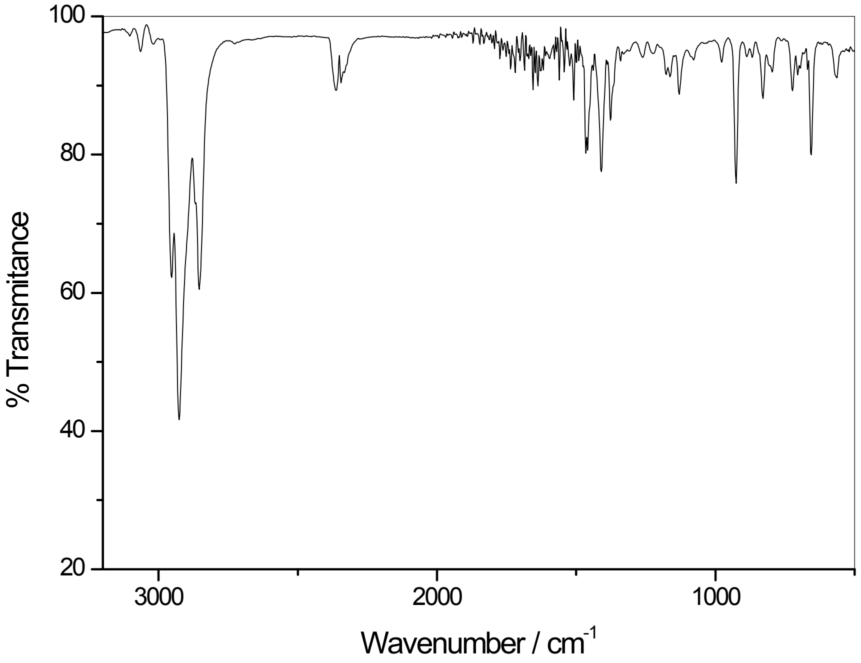

Figure S 34. FT-IR spectrum of derivative **3CPDTV.**

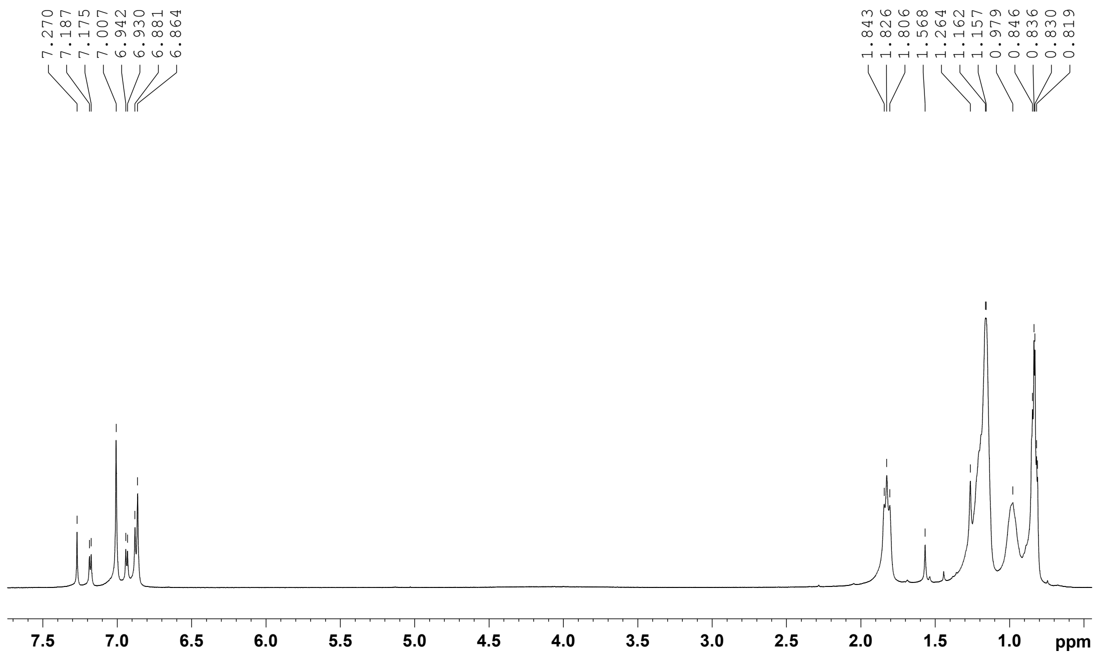

Figure S 35. ^{1}H NMR spectrum (400 MHz, $CDCl_3$) of derivative **4CPDTV.**

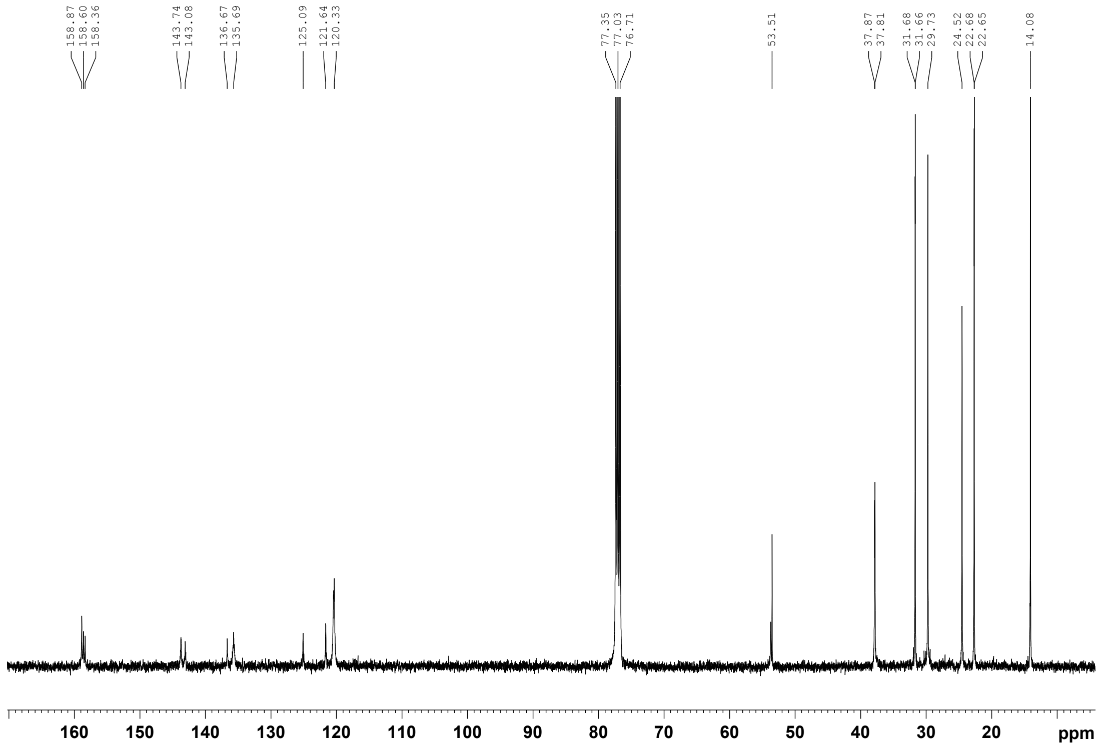

Figure S 36. ^{13}C NMR spectrum (100 MHz, $CDCl_3$) of **4CPDTV.**

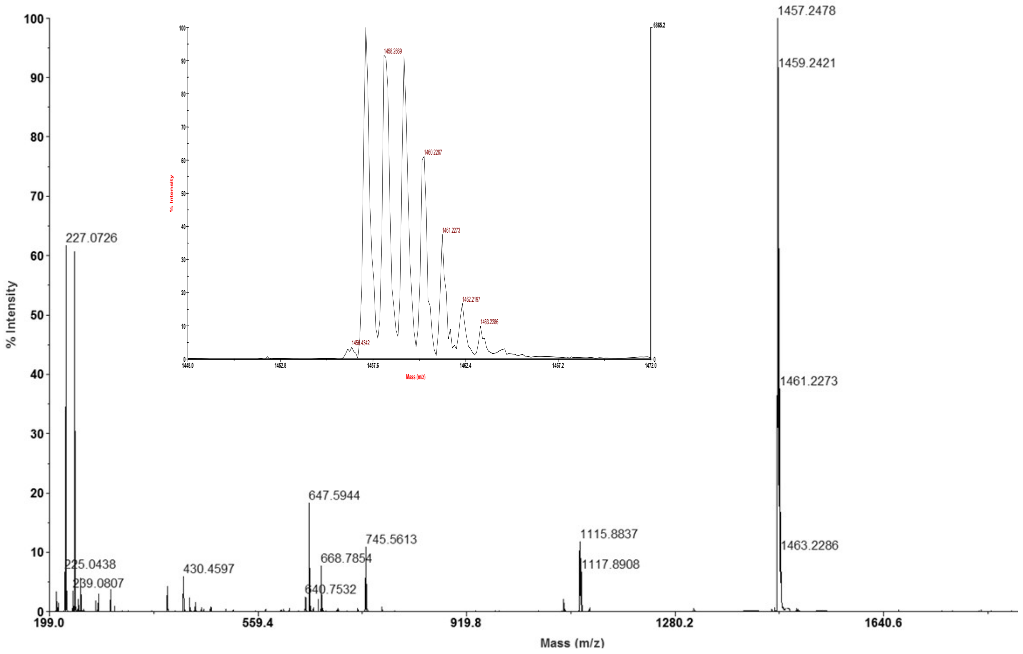

Figure S 37. MALDI-MS spectrum of **4CPDTV** (Matrix: Dithranol).

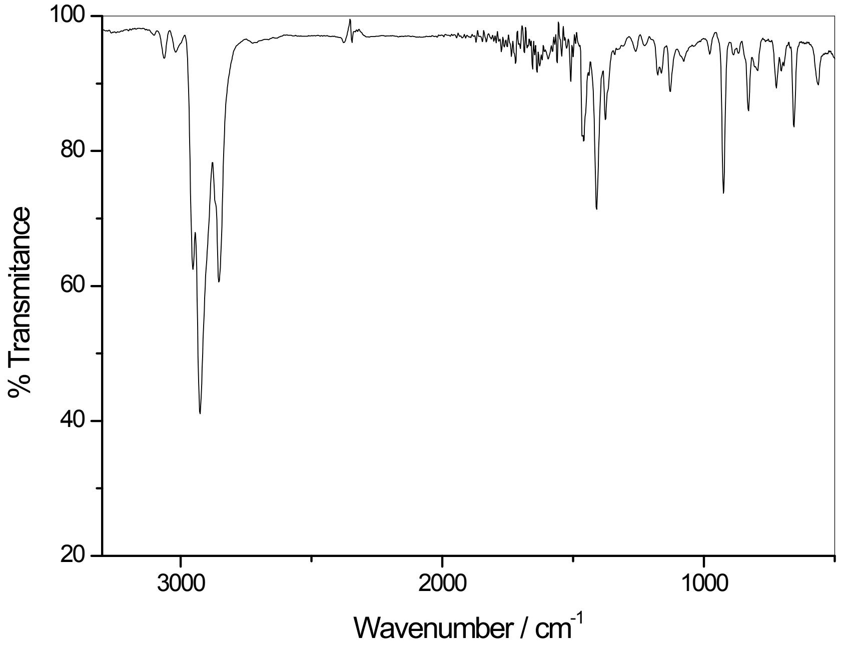

Figure S 38. FT-IR spectrum of **4CPDTV.**

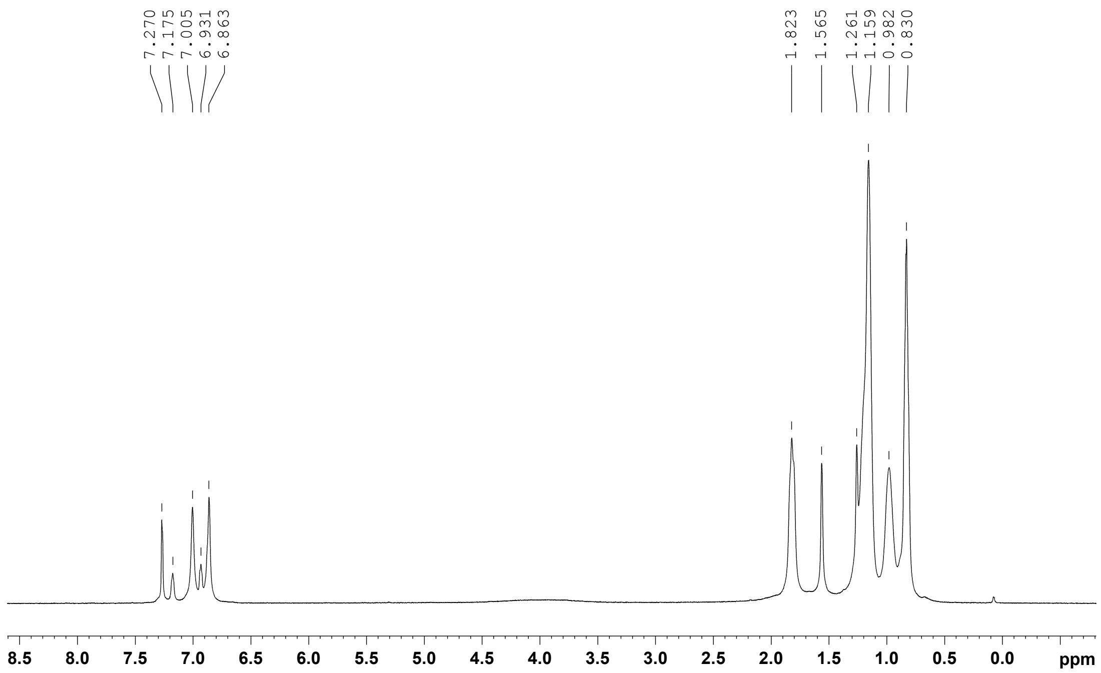

Figure S 39. ^{1}H NMR spectrum (400 MHz, $CDCl_3$) of **5CPDTV.**

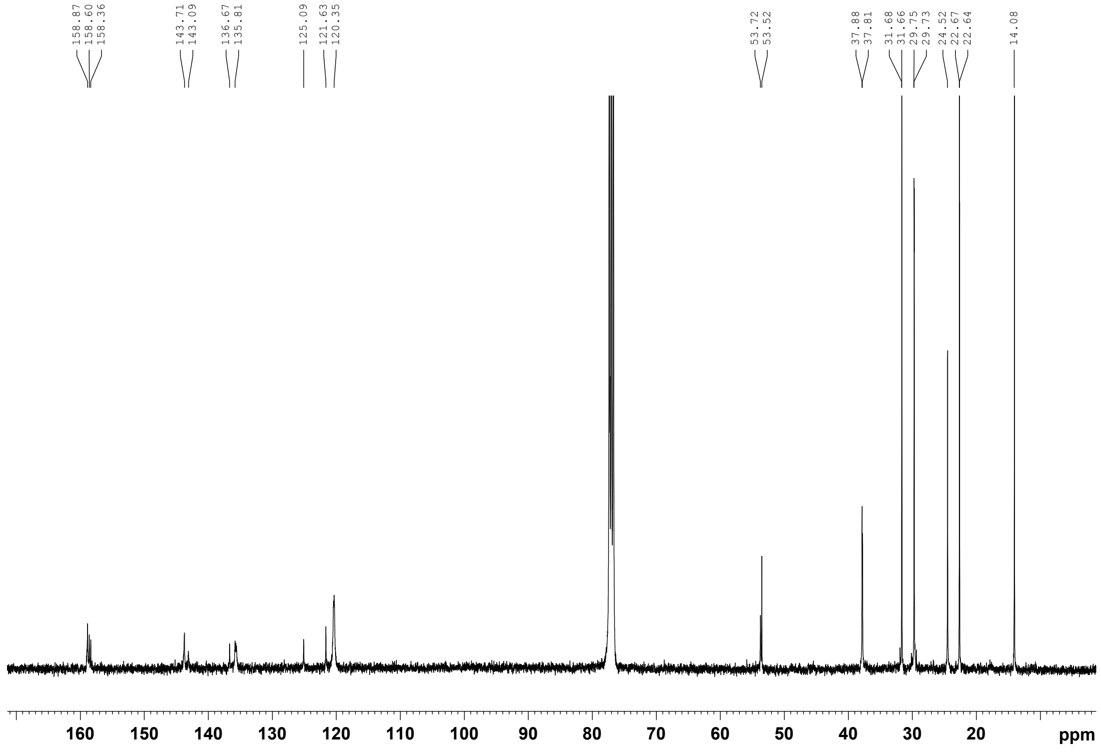

Figure S 40. ^{13}C NMR spectrum (100 MHz, $CDCl_3$) of **5CPDTV.**

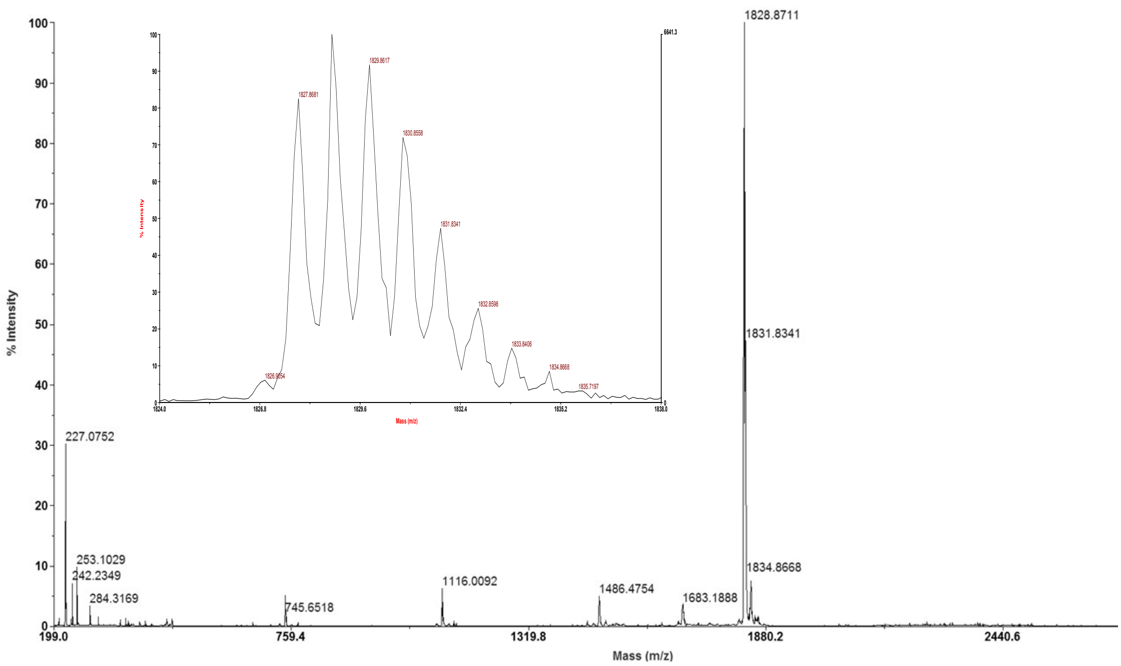

Figure S 41. MALDI-MS spectrum of **5CPDTV** (Matrix: Dithranol).

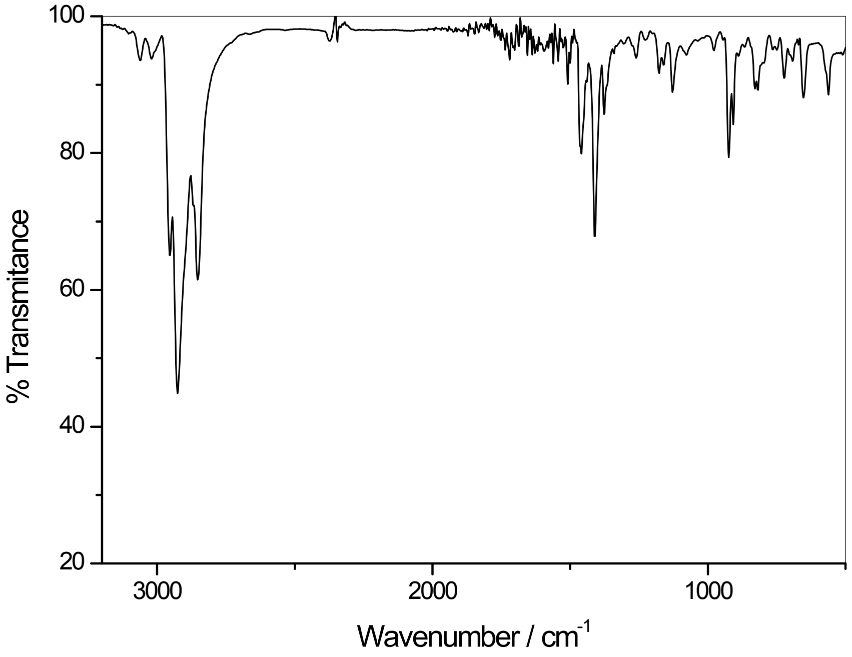

Figure S 42. FT-IR spectrum of **5CPDTV.**

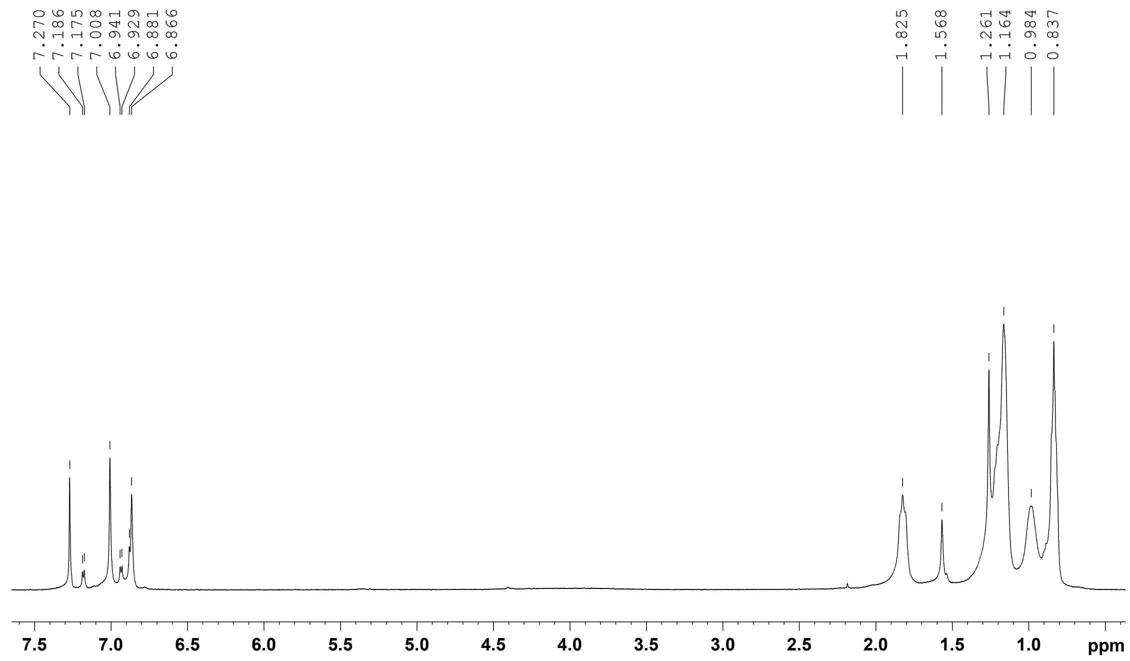

Figure S 43. ^{1}H NMR spectrum (400 MHz, $CDCl_3$) of **6CPDTV.**

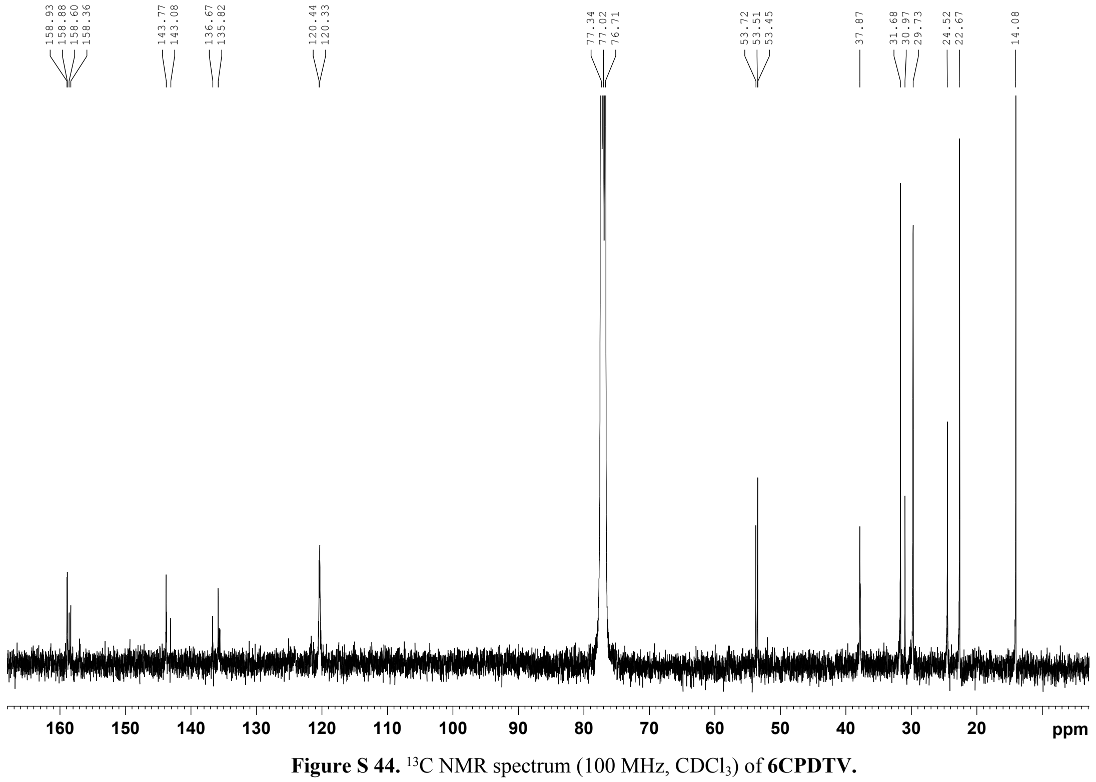

Figure S 44. ^{13}C NMR spectrum (100 MHz, $CDCl_3$) of **6CPDTV.**

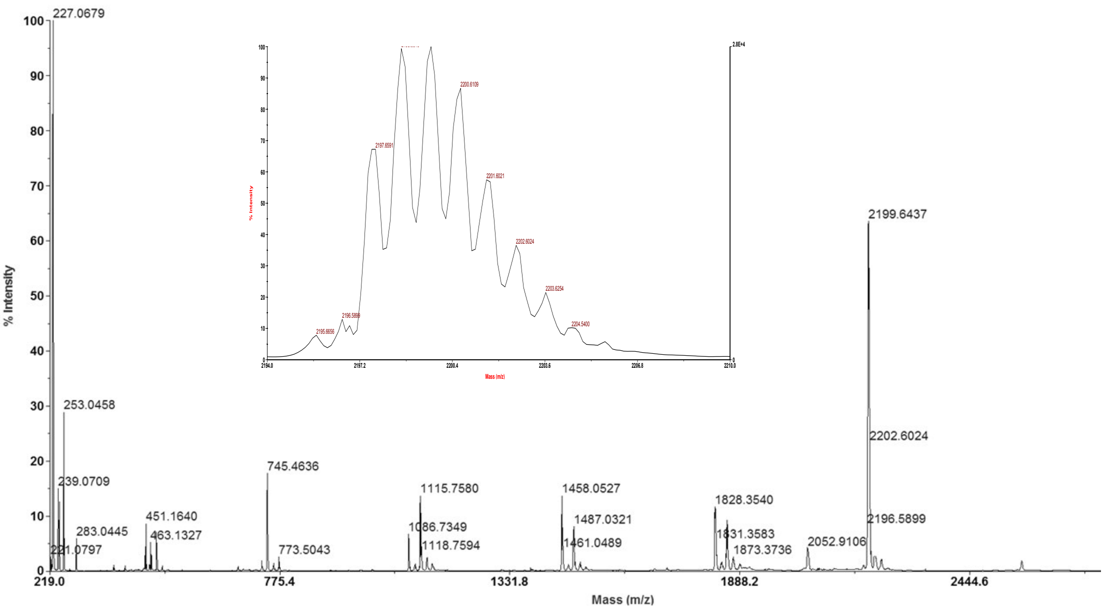

Figure S 45. MALDI-MS spectrum of **6CPDTV** (Matrix: Dithranol).

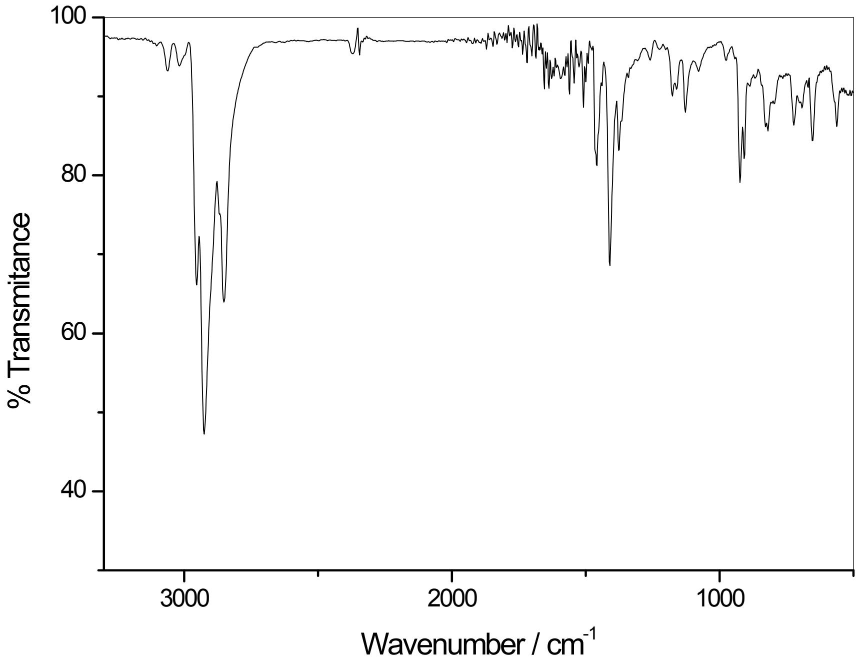

Figure S 46. FT-IR spectrum of **6CPDTV.**

4. Thermogravimetric analyses (TGA)

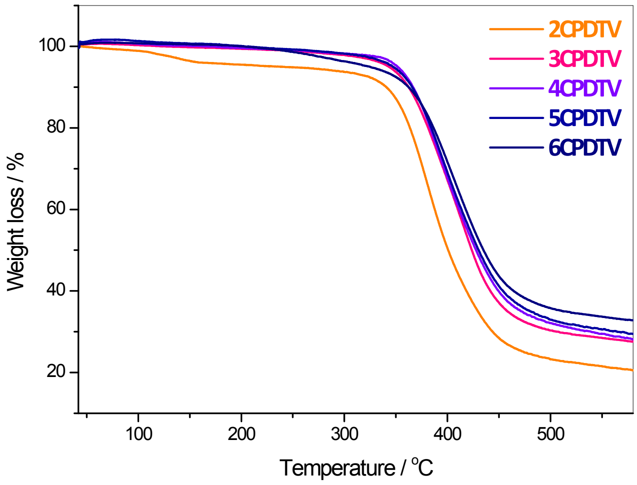

Figure S 47. TGA curves of derivatives **2-6CPDTV** at scan rate of 10 °C min^{-1}.

5. X-ray Crystallography of 9 (2CPDTV-Br)

Table S1 contains crystal data, collection parameters, and refinement criteria for the crystal structure of **9**. Crystals of **9** were grown by slow evaporation of CH2Cl2/heptane solutions. A crystal was placed on the tip of a Mitigen micromount and X-ray intensity data were measured at low temperature (173(2) K) with a graphite monochromated Mo Kα radiation (λ = 0.71073 Å) on a Rigaku XtaLAB mini diffractometer. The data were collected and processed using CrystalClear[9] and then solved and refined using SHELXL-97[10]. The structure consists of two bromo-substituted cyclopentadithiophene units linked by an vinyl bridge. The asymmetric unit is composed of two unique halves of these molecules. By inversion, the other halves of each unique molecule are generated. No solvent molecules were present in the structure and disorder was modeled in the hexyl chains of each half of the asymmetric unit. In the asymmetric unit containing the fragment with S1 and S2, the ensemble of the two hexyl chains was allowed to freely refine over two very similar sets of positions (C10 > C22, C10' > C22') with a ratio of 77/23 occupancies. In the asymmetric unit containing the fragment with S3 and S4, the ensemble of the two hexyl chains were modeled over a set of two less similar positions (C32 > C44, C32' > C44') with a fixed occupancy ratio of 50/50 as the atom position of C44 is very near a special position with inversion symmetry. An appropriate mix of restraints and constraints were used to model these disordered hexyl chain positions. EADP restraints were used to restrain thermal parameters in a pairwise fashion for the similar atom positions in the disorder of C10 > C22 and C10' > C22'. EADP restraints were used for the thermal paramaters of C32 and C32' as well. SIMU thermal parameter restraints were used for atoms C35' > C38'. Pairwise SADI distance restraints were used on the quaternary C10, C10', C32, and C32' atoms and first carbons of each hexyl chain (C11, C11', C17, C17', C33, C33', C39, C39'). SAME distance restraints were used to model the hexyl chains C11 > C16 with C11' > C16' and C17 > C22 with C17' > C22'. A DFIX restraint (1.52 Å) was placed on the atom distances C20-C21 and C20'-C21'. No disorder is present in the p-system backbone (except involvement of the quaternary C10, C10', C32, C32' positions). The π-system of each molecule is quite flat with a mean deviation from a least-squares plane of 0.018 Å (S1 part of asymmetric unit, major disorder component) and 0.056 Å (S3 part of asymmetric unit). The angle between the π-system least-squares planes of the two unique molecules is 68.7°. No close

[9] Rigaku Americas and Rigaku (2011) CrystalClear. Rigaku Americas, The Woodlands, TX.
[10] Sheldrick, G. M. *Acta Cryst.* **2008**, *A64*, 112-122.

intermolecular π–π contacts are present as the hexyl chain orientations preclude close approach. The packing can be described as neither layered nor quite herringbone. The hexyl chains adopt orientations that do not align with unit cell axes, nor the π-systems. The orientation of the alkene is trans as required by the centrosymmetric nature of these asymmetric units. Representations of the unique subunits are shown in Figures S48 and S49.

Table S 1 Crystal data and structure refinement for **9**.

Empirical Formula	$C_{44}H_{58}Br_2S_4$
Temperature (K)	173 (2)
Crystal System	triclinic
Space Group	P-1 (#2)
a (Å)	12.463(2)
b (Å)	13.493(3)
c (Å)	15.328(3)
α (°)	64.971(5)
β (°)	73.140(5)
γ (°)	82.198(6)
V (Å^3)	2234.9(7)
Z	2
D (g/cm^3)	1.300
μ (cm^{-1})	20.340
No. of Reflections Measured	21,589
Unique Reflections	9121
R1 (I>2.00□(I))	0.0543
wR2 (All reflections)	0.1434
Goodness of Fit	1.028

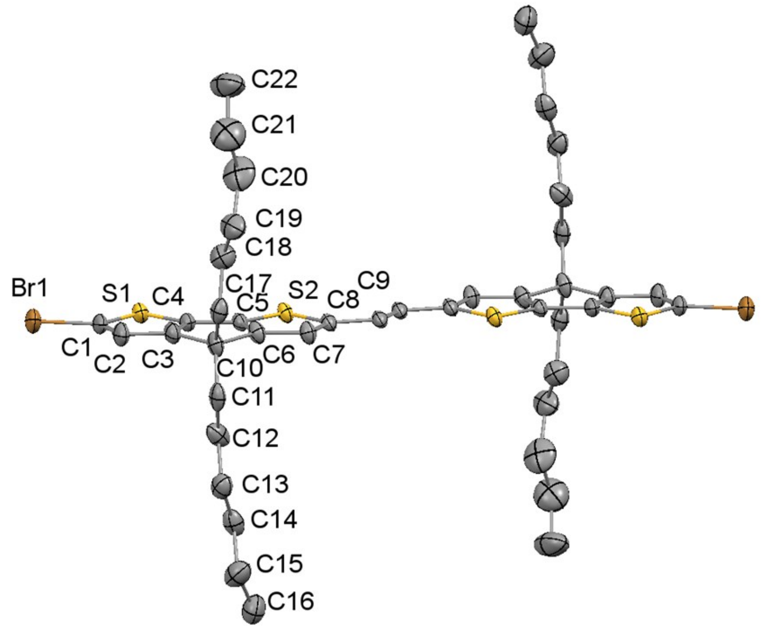

Figure S 48. One half of the asymmetric unit of **9** (remaining positions generated by inversion).

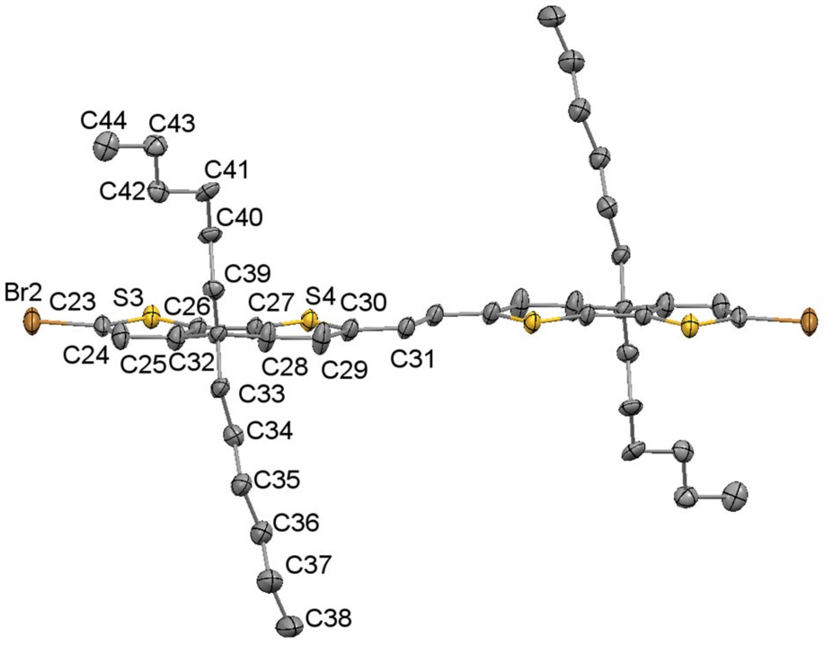

Figure S 49 One half of the asymmetric unit of **9** (remaining positions generated by inversion).

6. Theoretical analysis of Raman spectra of nCPDTV.

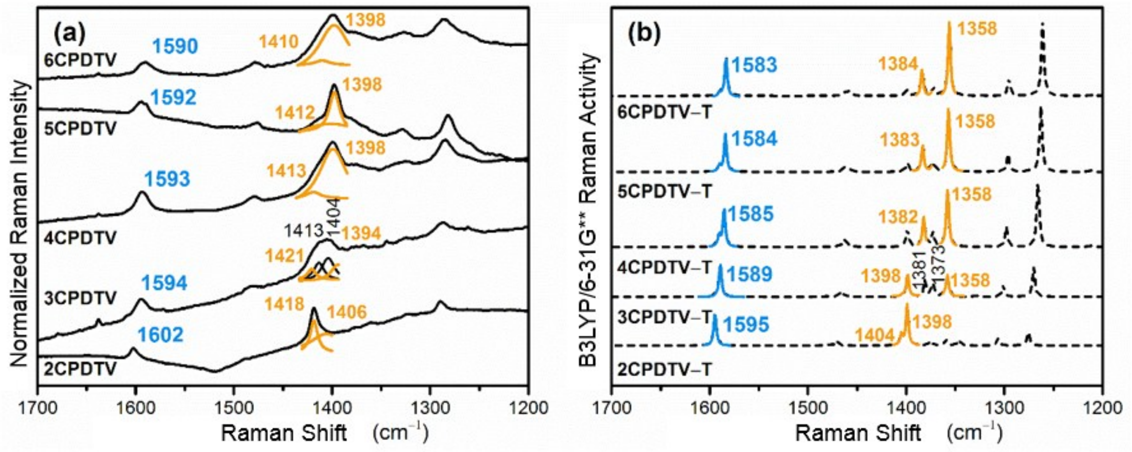

Figure S 50 (a) Solid-state Raman (λ = 785 nm) spectra of **nCPDTV** recorded at room temperature. $\nu(C{=}C)_{vinyl}$ in blue, $\nu(C{=}C)_{CPDT}$ in orange, The frequencies have been assigned by deconvolution. (b) DFT/B3LYP/6-31G** theoretical Raman spectra of **nCPDTV-T.**

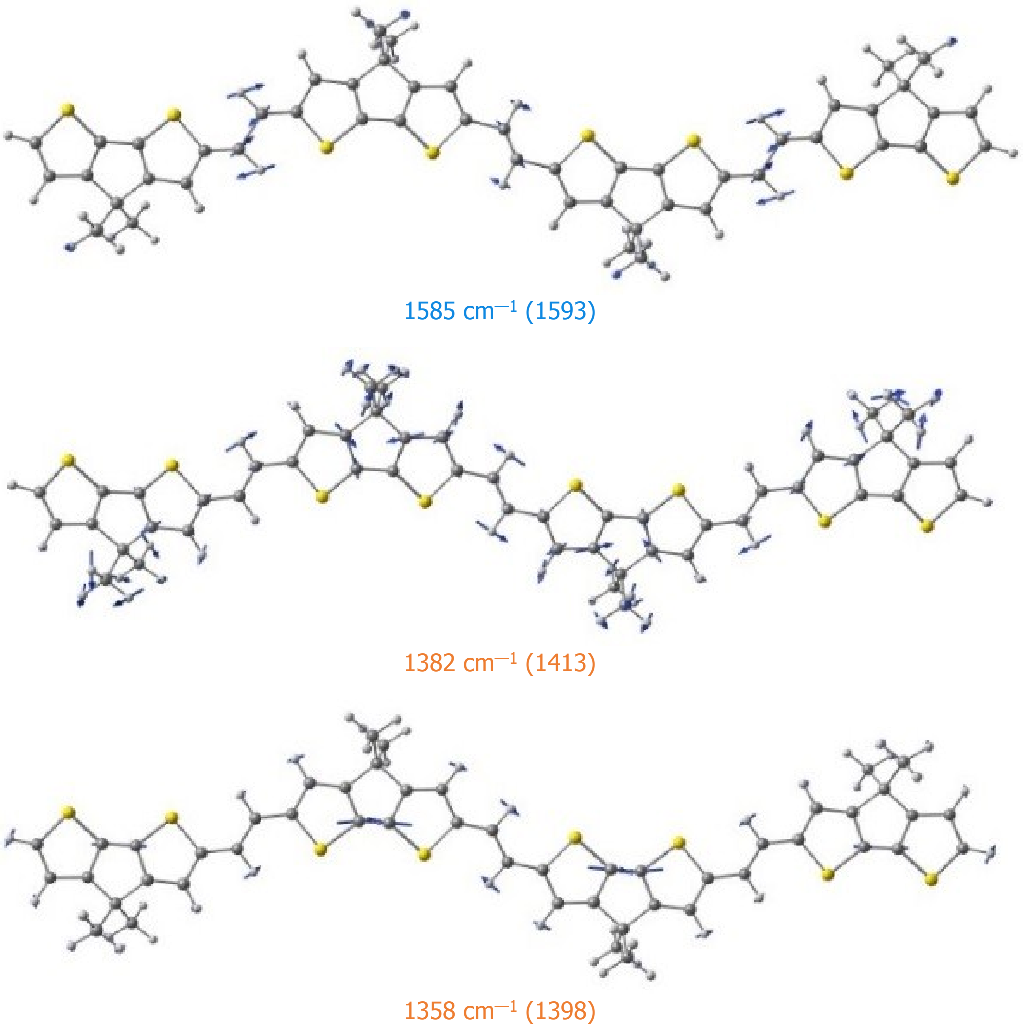

Figure S 51. Vibrational eigenvectors associated with the most relevant bands of the DFT/B3LYP/6-31G** Raman spectrum of **4CPDTV-T**. In parentheses, experimental values are shown. R- chains have been omitted for clarity. $\nu(C{=}C)_{vinyl}$ (blue) y $\nu(C{=}C)_{CPDT}$ (orange).

7. Atomic Force Microscopy Morphology

The samples investigated by AFM were prepared by drop-casting on HOPG and mica substrates of 20 µL of 10^{-6} mg/mL dichloromethane solutions of the **2-6CPTDV** oligomers. This concentration was selected after the development of an intensive study of the behavior of the **CPTDV** compounds on surface attending to the concentration and substrate nature. In all the cases, the best concentration to perform the study was 10^{-6} mg/mL. The assembly of the **nCPTDV** compounds only take place on HOPG in form of long fibers. The height and length of the fibers is heterogeneous in a same sample. It is important to note, that as it can be observed in figure S52 the length and height of the unidimensional structures seems to decrease from **2CTDPV** to **6CPTDV**.

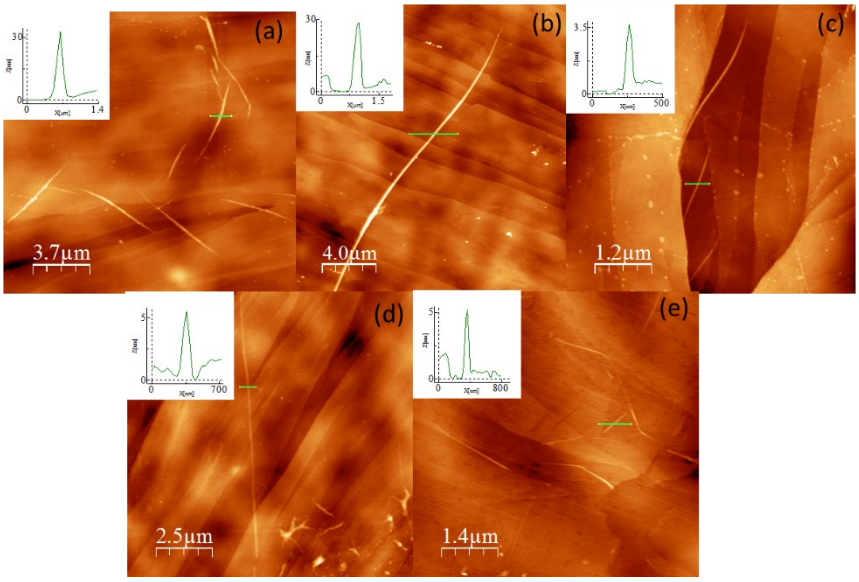

Figure S 52. AFM topography images of **2CPTDV** (a) **3CPTDV** (b) **4CPTDV** (c) **5CPTDV** (d) **6CPTDV** (e) drop casted on HOPG. An unidimensional organization of the **nCPTDV** oligomers can be observed for all the cases.

An important parameter involved in the organization on surface of the **nCPTDV** oligomers is the substrate nature. The unidimensional assembly was only observed on HOPG. Because of this reason it is considered that the fiber formation is promoted by the presence of π-π interactions. On mica, the formation of layers was observed in the case of **4CPTDV** and the organization of micrometric fibers in the case of **5CPTDV** as shown in figure S53.

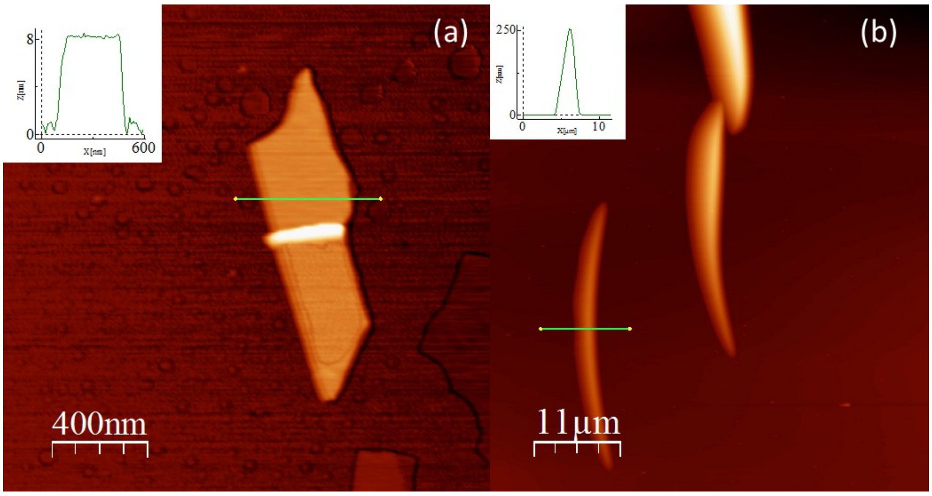

Figure S 53. **4CPTDV** layers observed on mica (a) and micrometric fibers of **5CPTDV** organized on mica surface (b).

8. Ciclic Voltammetry (CV) and Osteryoung Square Wave Voltammetry (OSWV) spectra

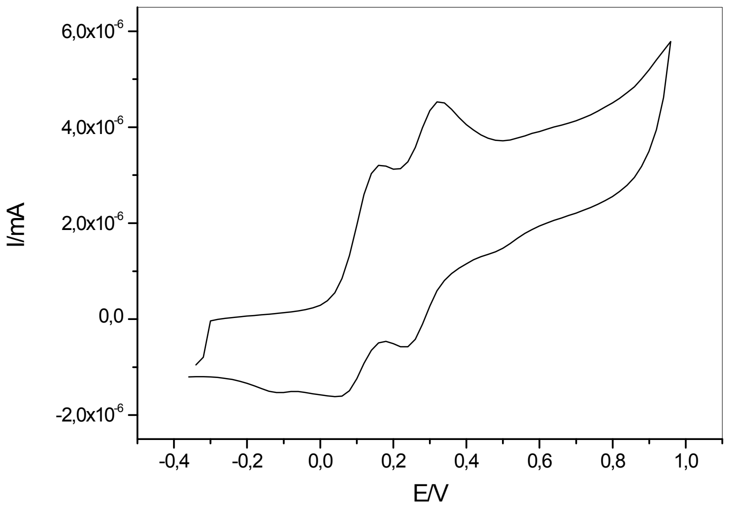

Figure S 54. CV plot of **2CPDTV** (referred to Fc/Fc+).

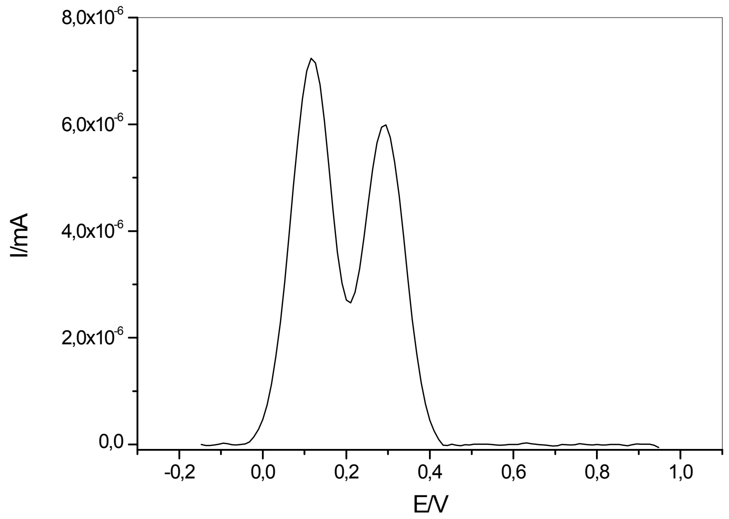

Figure S 55. OSWV plot of **2CPDTV** (referred to Fc/Fc+).

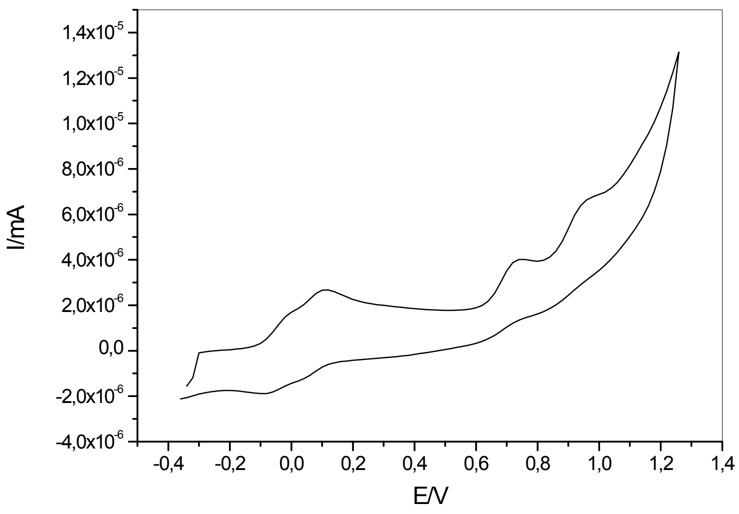

Figure S 56. CV plot of **3CPDTV** (referred to Fc/Fc^{+}).

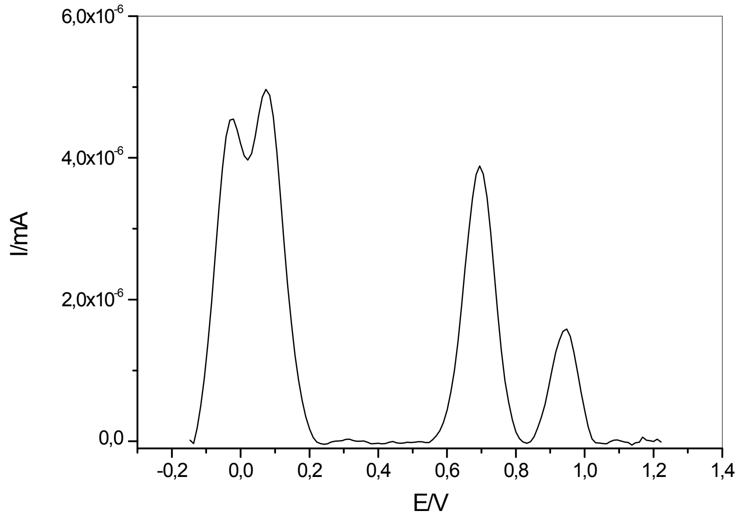

Figure S 57. OSWV plot of **3CPDTV** (referred to Fc/Fc^{+}).

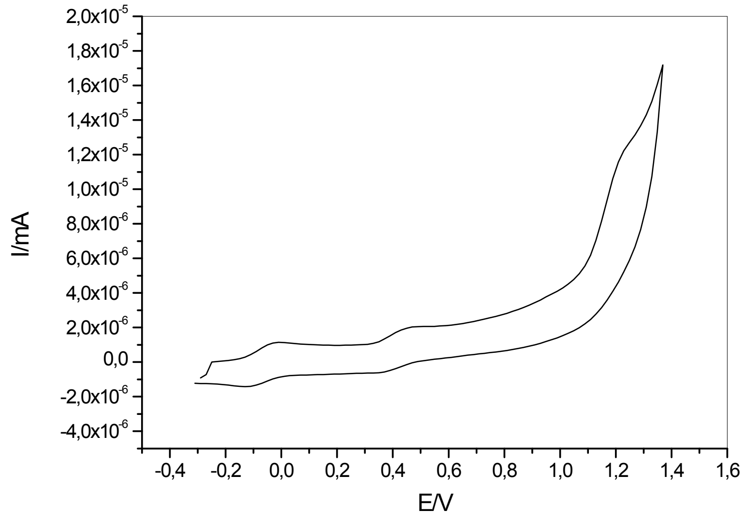

Figure S 58. CV plot of **4CPDTV** (referred to Fc/Fc+).

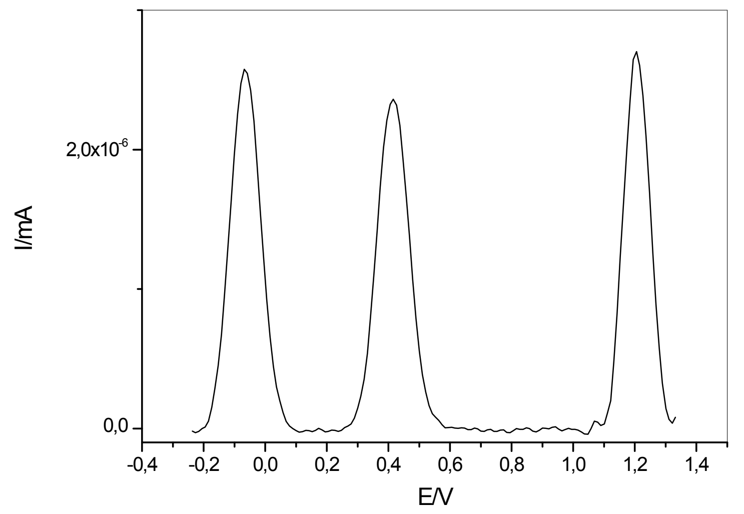

Figure S 59. OSWV plot **4CPDTV** (referred to Fc/Fc+).

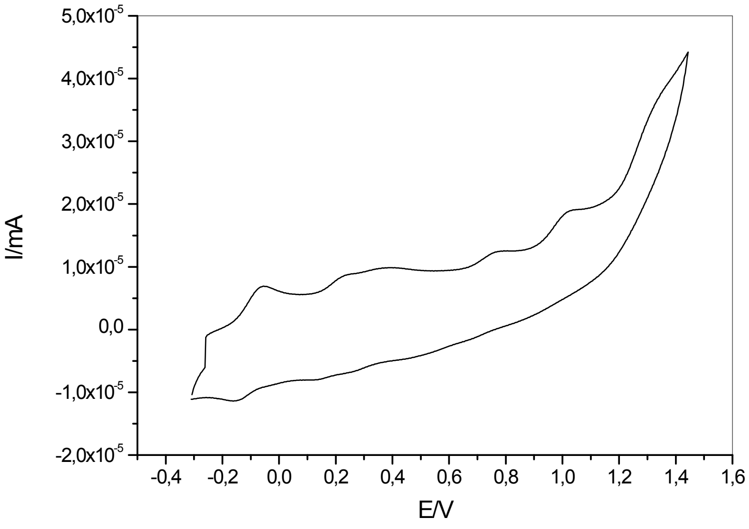

Figure S 60. CV plot of derivative **5CPDTV** (referred to Fc/Fc+).

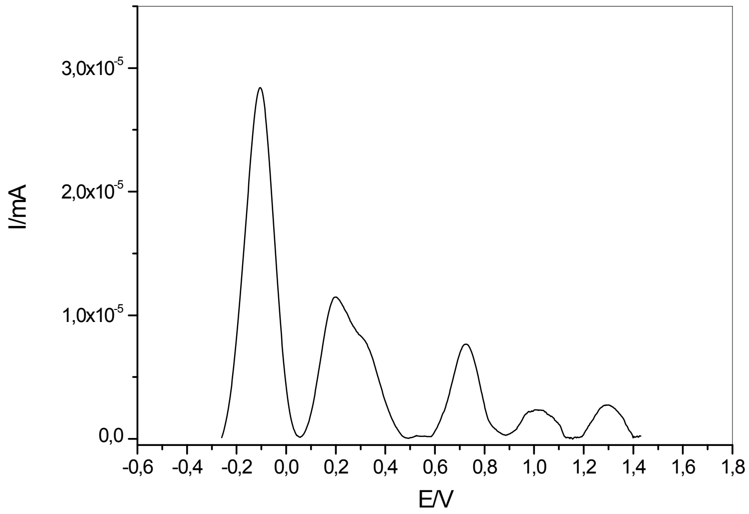

Figure S 61. OSWV plot of derivative **5CPDTV** (referred to Fc/Fc+).

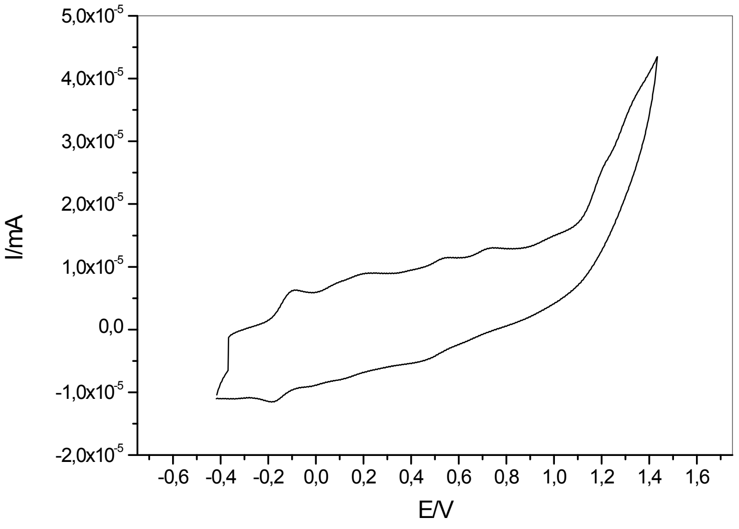

Figure S 62. CV plot of derivative **6CPDTV** (referred to Fc/Fc^{+})

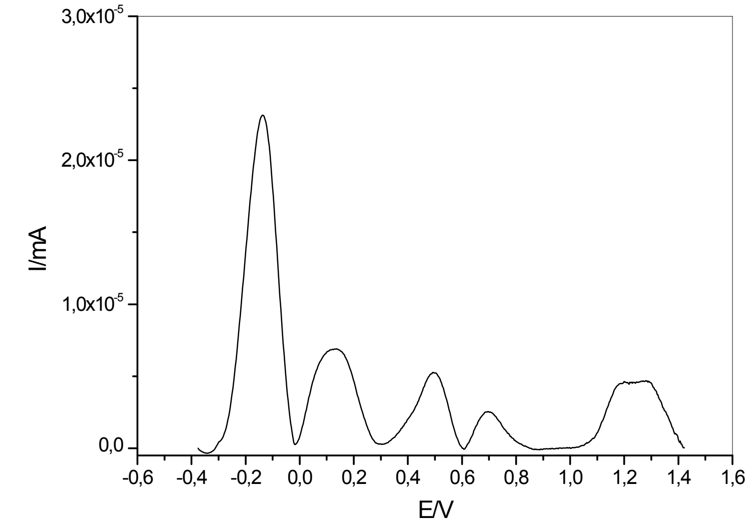

Figure S 63. OSWV plot of derivative **6CPDTV** (referred to Fc/Fc^{+}).

9. Generation of positive charged species in nCPDTV

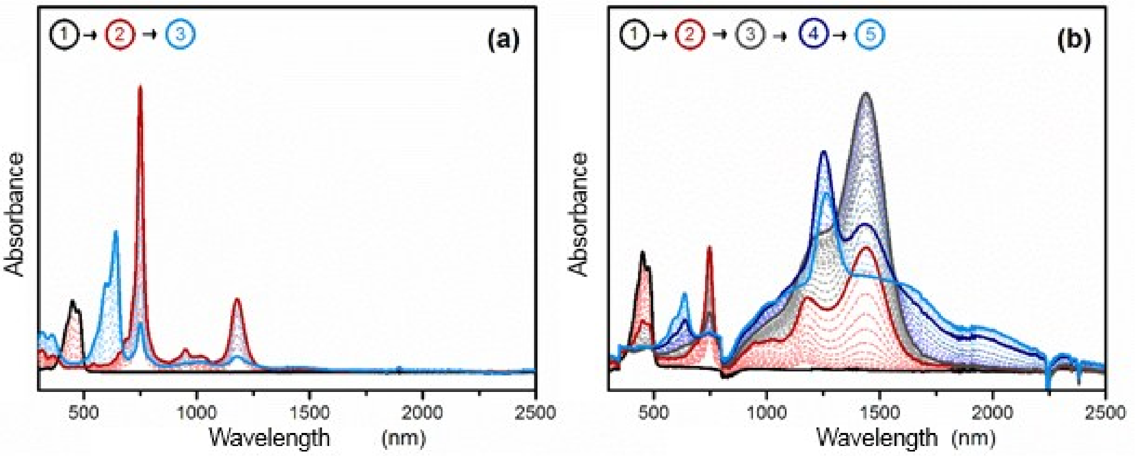

Figure S 64. (a) UV−Vis−NIR absorption spectra of the chemically oxidised species of **2CPDTV** obtained in DCM solutions at room temperature by stepwise addition of a solution of $FeCl_3$ in DCM. (b) UV−Vis−NIR absorption spectra of the electrochemically oxidised species obtained in 0.1 M TBA-PF_6/ DCM solution at room temperature for **2CPDTV**. **2CPDTV**: black, **[2CPDTV]**$^{\bullet+}$: red, **[σ—2CPDTV]**$_y^{x1+}$: light blue, **[2CPDTV]**$_{ag1}^{x2+}$: grey and **[2CPDTV]**$_{ag2}^{x3+}$: blue.

Table S 2 Absorption maxima (nm) relative to the positive charged species of Figure S 64**.**

(a)	2CPDTV	[2CPDTV]$^{\bullet+}$	[σ—2CPDTV]$_y^{x,+}$
	450, 480	750, 950, 1023, 1181	642, 1024

(b)	2CPDTV	[2CPDTV]$^{\bullet+}$	[2CPDTV]$_{ag1}^{x,+}$	[2CPDTV]$_{ag2}^{x,+}$	[σ—2CPDTV]$_y^{x,+}$
	450, 480	747, 950, 1023, 1181 (1441)	1439	(636) 1253 (1437)	636

According to Figure S 64 (a), during the first step of the chemical oxidation process, the absorption band of the neutral species, in black, progressively disappear and is replaced, through isosbestic points, by two new bands (750 and 1181nm), relative to the radical cation (**[2CPDTV]**$^{\bullet+}$, red). Moving on to the second step, chemical oxidation of the radical catión leads to the appearence of a new species (642 nm), inconsistent with the generation of a quinoidal dication. Thus, irreversible chemical reactions may generate s dimerization (**[σ—2CPDTV]**$_y^{x1,+}$, light blue).

UV−Vis−NIR absorption spectra of the electrochemically oxidised species (Figure S 63(b)), confirm the existence of irreversible chemical reactions thanks to the appearance of the same band previously assigned to **[σ—2CPDTV]**$_y^{x1+}$ (636 nm). However, in this occasion, several intermediate species, with strong absorption bands in the NIR region, are observed after the generation of the radical cation. They have been linked to possible molecular aggregates, favoured by higher concentration and electrolytic medium (**[2CPDTV]**$_{ag1}^{x2,+}$, grey and **[2CPDTV]**$_{ag2}^{x3,+}$, blue).

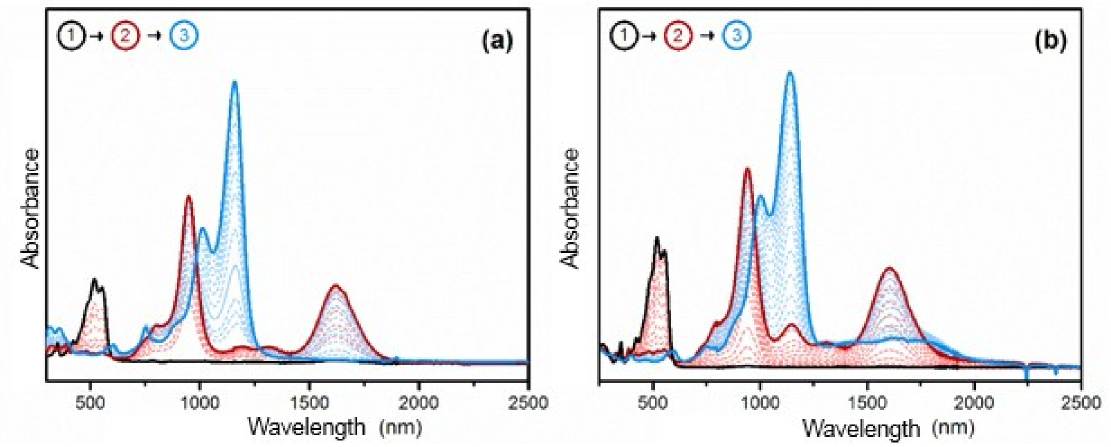

Figure S 65. 3CPDTV: (a) UV−Vis−NIR absorption spectra of the chemically oxidised species obtained in DCM solutions at room temperature by stepwise addition of a solution of $FeCl_3$ in DCM. (b) UV−Vis−NIR absorption spectra of the electrochemically oxidised species obtained in 0.1 M TBA-PF_6/ DCM solution at room temperature. **3CPDTV**: black, **[3CPDTV]**$^{\bullet+}$: red and **[3CPDTV]**$^{2+}$: blue.

Table S 3 Absorption maxima (nm) relative to the positive charged species of Figure S 65**.**

	**3CPDTV**	**[3CPDTV]**$^{\bullet+}$	**[3CPDTV]**$^{2+}$
(a)	520, 554	948 (1331, 1981), 1618	1012, 1157
(b)	520, 554	940 (1147, 1313), 1605	1002, 1138

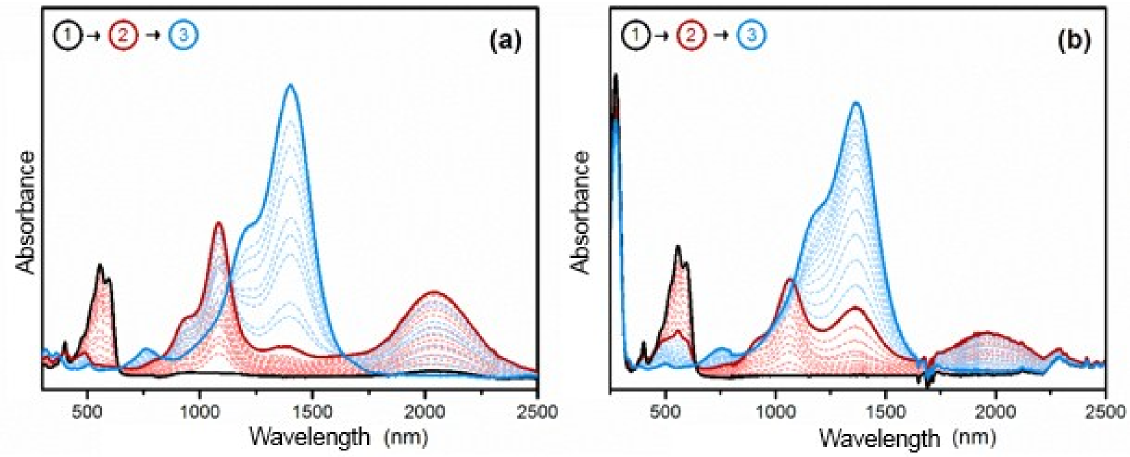

Figure S 66. 4CPDTV: (a) UV−Vis−NIR absorption spectra of the chemically oxidised species obtained in DCM solutions at room temperature by stepwise addition of a solution of $FeCl_3$ in DCM. (b) UV−Vis−NIR absorption spectra of the electrochemically oxidised species obtained in 0.1 M TBA-PF_6/ DCM solution at room temperature. **4CPDTV**: black, **[4CPDTV]**$^{\bullet+}$: red and **[4CPDTV]**$^{2+}$: blue.

Table S 4 Absorption maxima (nm) relative to the positive charged species of Figure S 66**.**

	**4CPDTV**	**[4CPDTV]**$^{\bullet+}$	**[4CPDTV]**$^{2+}$
(a)	560, 598	1082, (1378), 2041	1402
(b)	556, 595	1067, (1365), 1963	1365

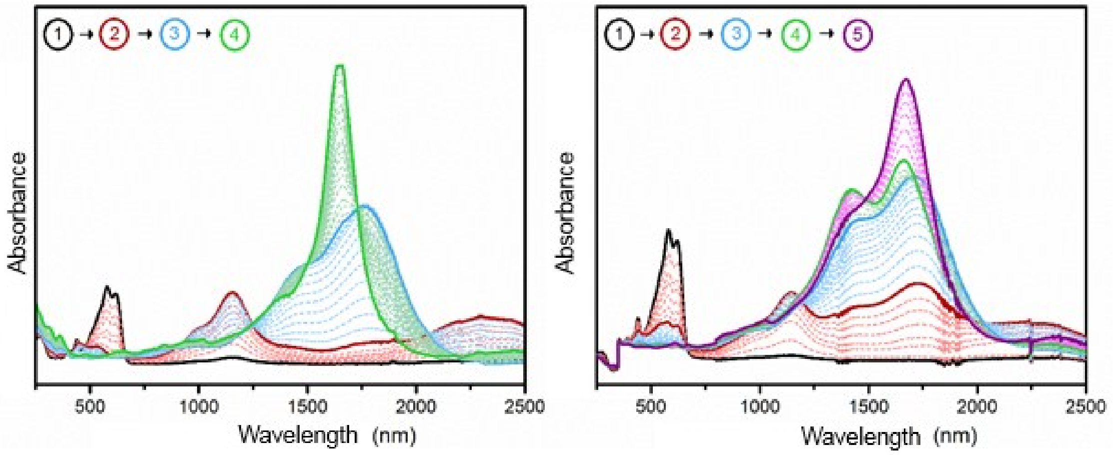

Figure S 67. 5CPDTV: (a) UV−Vis−NIR absorption spectra of the chemically oxidised species obtained in DCM solutions at room temperature by stepwise addition of a solution of $FeCl_3$ in DCM. (b) UV−Vis−NIR absorption spectra of the electrochemically oxidised species obtained in 0.1 M TBA-PF_6/ DCM solution at room temperature. **5CPDTV**: black, **[5CPDTV]**$^{\bullet+}$: red, **[5CPDTV]**$^{2+}$: blue, **[5CPDTV]**$^{\bullet 3+}$: green and **[5CPDTV]**$^{4+}$: purple.

Table S 5 Absorption maxima (nm) relative to the positive charged species of Figure S 67**.**

	5CPDTV	$[5CPDTV]^{\bullet+}$	$[5CPDTV]^{2+}$	$[5CPDTV]^{\bullet 3+}$	$[5CPDTV]^{4+}$
(a)	581,621	1160, 2301	1761	1640	—
(b)	582,622	1146, (1736), 2284	1716	1423, 1664	1674

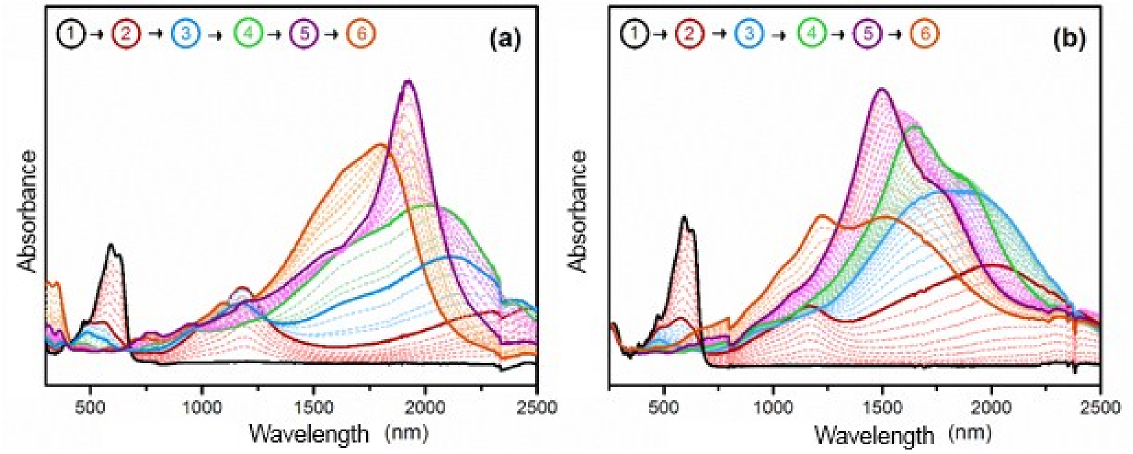

Figure S 68. 6CPDTV: (a) UV−Vis−NIR absorption spectra of the chemically oxidised species obtained in DCM solutions at room temperature by stepwise addition of a solution of $FeCl_3$ in DCM. (b) UV−Vis−NIR absorption spectra of the electrochemically oxidised species obtained in 0.1 M TBA-PF_6/ DCM solution at room temperature. **6CPDTV**: black, **[6CPDTV]**$^{\bullet+}$: red and **[6CPDTV]**$^{2+}$: blue, **[6CPDTV]**$^{\bullet 3+}$: green, **[6CPDTV]**$^{4+}$: purple and **[6CPDTV]**$^{\bullet 5+}$: orange.

Table S 6 Absorption maxima (nm) relative to the positive charged species of Figure S 68**.**

	6CPDTV	$[6CPDTV]^{\bullet+}$	$[6CPDTV]^{2+}$	$[6CPDTV]^{\bullet 3+}$	$[6CPDTV]^{4+}$	$[6CPDTV]^{\bullet 5+}$
(a)	594, 632	1184, 2382	1178, 2121	2016	1922	1800
(b)	593, 630	1170, 2000	1852	1652	1497	1227, 1527

10. Theoretical evolution of ν(C=C)vinyl in nCPDTV-T charged species.

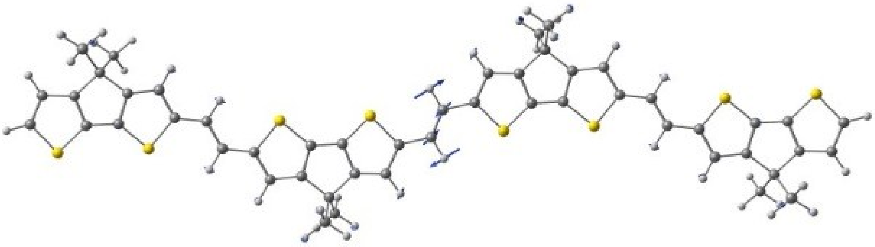

[**4CPDTV—T]**$^{\bullet+}$: 1550 cm^{-1} (1568)

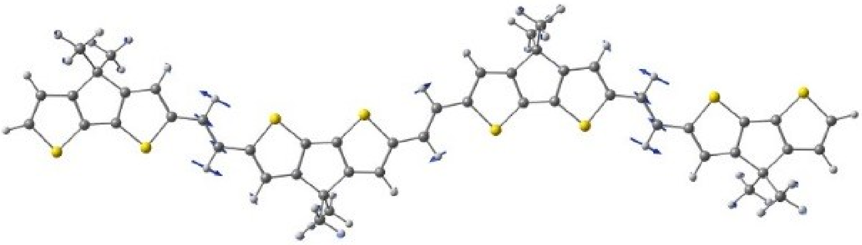

[**4CPDTV—T]**$^{2+}$: 1556 cm^{-1} (1568)

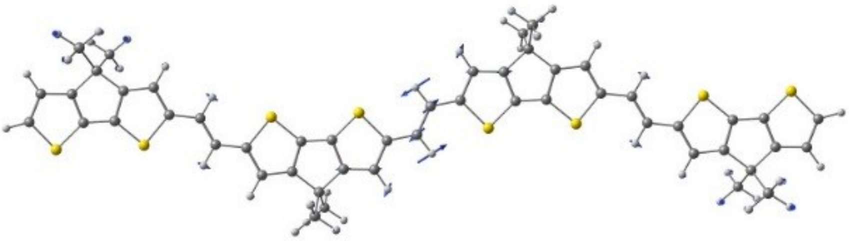

[**4CPDTV—T]**$^{2+}$: 1547 cm^{-1} (1568)

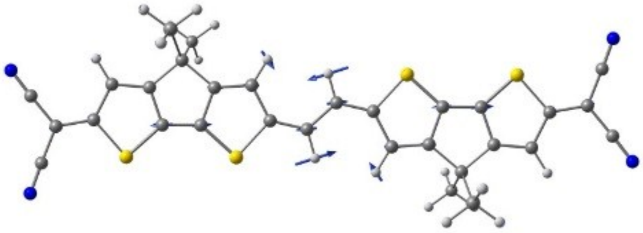

Q(2CPDTV) —T: 1519 cm^{-1} (1543)

Figure S 69. DFT/(U)B3LYP/6-31G** vibrational eigenvectors for ν(C=C)$_{vinyl,}$ of **4CPDTV-T** charged species, together with **Q(2CPDTV) -T**. In parentheses, experimental values are shown. R- chains have been omitted for clarity.
